# Genome sequence of the necrotrophic fungus *Penicillium digitatum*, the main postharvest pathogen of citrus

**DOI:** 10.1186/1471-2164-13-646

**Published:** 2012-11-21

**Authors:** Marina Marcet-Houben, Ana-Rosa Ballester, Beatriz de la Fuente, Eleonora Harries, Jose F Marcos, Luis González-Candelas, Toni Gabaldón

**Affiliations:** 1 Centre for Genomic Regulation (CRG), Dr. Aiguader 88, Barcelona, 08003, Spain; 2 Universitat Pompeu Fabra (UPF), Barcelona, 08003, Spain; 3 Instituto de Agroquímica y Tecnología de Alimentos (IATA-CSIC), Avda. Agustin Escardino 7, Paterna, Valencia, 46980, Spain

## Abstract

**Background:**

*Penicillium digitatum* is a fungal necrotroph causing a common citrus postharvest disease known as green mold. In order to gain insight into the genetic bases of its virulence mechanisms and its high degree of host-specificity, the genomes of two *P. digitatum* strains that differ in their antifungal resistance traits have been sequenced and compared with those of 28 other Pezizomycotina.

**Results:**

The two sequenced genomes are highly similar, but important differences between them include the presence of a unique gene cluster in the resistant strain, and mutations previously shown to confer fungicide resistance. The two strains, which were isolated in Spain, and another isolated in China have identical mitochondrial genome sequences suggesting a recent worldwide expansion of the species. Comparison with the closely-related but non-phytopathogenic *P. chrysogenum* reveals a much smaller gene content in *P. digitatum*, consistent with a more specialized lifestyle. We show that large regions of the *P. chrysogenum* genome, including entire supercontigs, are absent from *P. digitatum*, and that this is the result of large gene family expansions rather than acquisition through horizontal gene transfer*.* Our analysis of the *P. digitatum* genome is indicative of heterothallic sexual reproduction and reveals the molecular basis for the inability of this species to assimilate nitrate or produce the metabolites patulin and penicillin. Finally, we identify the predicted secretome, which provides a first approximation to the protein repertoire used during invasive growth.

**Conclusions:**

The complete genome of *P. digitatum*, the first of a phytopathogenic *Penicillium* species, is a valuable tool for understanding the virulence mechanisms and host-specificity of this economically important pest.

## Background

Postharvest losses can have a significant impact on crops, ranging from 15 to as high as 50% of total production [1]. Citrus is one of the most economically important fruit crops in the world and it is particularly susceptible to postharvest damage because harvested fruits are usually stored before they reach the market for fresh consumption, a period in which they are subject to both biotic and abiotic stress conditions. Fungi, which are particularly adapted to a saprophytic lifestyle, are among the main biological agents causing crop deterioration during transport and storage. In citrus, a common postharvest disease known as green mold and caused by *Penicillium digitatum* can account for up to 90% of the total losses, especially in arid and sub-tropical climates [2]. Nowadays, the control of post-harvest decay is achieved by the massive application of fungicides to marketed fruits. This however, has serious implications regarding toxicity, consumer acceptance and environmental risk. In this sense, reports of selection for fungicide resistant strains are increasing and represent a significant obstacle to postharvest conservation and commerce [3]. *P. digitatum* is a necrotrophic wound pathogen that requires pre-existing injured fruit peel to penetrate the plant tissue, and it colonizes mostly through the deployment of maceration enzymes. Remarkably, and despite this rather unspecific infection mechanism, *P. digitatum* exhibits a high degree of host specificity and has not been described as ‘naturally-occurring’ in other pathosystems outside citrus fruits [4]. Despite its considerable economic interest, the molecular bases of *P. digitatum* infection and host specificity remain largely unknown. We have previously analyzed the fruit’s transcriptomic response to *P. digitatum* infection and also to elicitors that trigger induced resistance [5,6]. Here, we report the complete genome sequence of two *P. digitatum* strains that differ in their resistance to common fungicides and a comprehensive comparison across 28 other sequenced Pezizomycotina. This resource will enable progress to be made towards understanding the physiology and virulence mechanisms of this economically important phytopathogen.

## Results and discussion

### Genome sequence

Two previously described *P. digitatum* strains isolated from infected orange (PHI26) and grapefruit (Pd1) in Valencia (Spain) [7,8] were selected for whole-genome sequencing. These strains differ in their antifungal resistance properties, Pd1 being resistant to thiabendazole and imazalil, the two fungicides most commonly used in citrus postharvest. Their genomes were sequenced with either Roche 454 pyrosequencing (Pd1) or Illumina Hiseq 2000 (PHI26), which also served to compare both sequencing strategies. Final assemblies (Table 1) resulted in similar genome size estimates of around 26 Mb with an average GC content of 48.9%. The 454-sequenced Pd1 strain showed a smaller number of scaffolds and a larger N50 than the Illumina-sequenced PHI26 strain, but PHI26 showed improved assembly parameters in terms of coverage, number of contigs and number of undetermined sites. Since this latter property was found to significantly affect gene prediction quality (see below), PHI26 was used as the reference genome in further analyses. RepeatMasker [9] identified 1.39% of the genome as being repetitive or corresponding to transposable elements (Additional file 1: Table S1). This value is similar to the 1.44% found for *P. chrysogenum*, though there were differences in the distributions of the various types of elements. Using a combined approach that included the use of RNA-Seq data, we predicted approximately 9,000 protein-coding genes in each of the two strains. Predicted genes include 100% of a set of 69 core genes found to be ubiquitous across all fungal clades [10], attesting to the high coverage of our assembly and the reliability of the protein-coding gene prediction. Protein-coding genes were functionally annotated using a combination of domain mapping and the transfer of functional annotations based on phylogenetic assignments of orthology relationships [11,12]. The complete sequences and predicted genes for these Whole Genome Shotgun projects have been deposited at DDBJ/EMBL/GenBank under the accession numbers AKCT00000000 (PHI26) and AKCU00000000 (Pd1). The versions described in this paper are the first versions, AKCT01000000 and AKCU01000000, respectively, and provide a valuable tool for the discovery of genes important for *P. digitatum* physiology and pathogenicity. In the current study we explore the newly obtained genomes to gain insight into some of the main aspects of *P. digitatum* physiology.

**Table 1:** **Summary of the main assembly and annotation features of the genomes of the two sequenced *Penicillium digitatum *strains**

***P. digitatum *strain**	**PHI26**	**Pd1**
**Sequencing technology**	Illumina (HiSeq 2000)	Roche 454
**Genome size**	26 (Mb)	26 (Mb)
**Sequencing coverage**	83x	24x
**Number of contigs**	287	544
**Number of scaffolds**	102	54
**Number of Large Scaffolds (>100 Kb)**	36	26
**N50 (base pairs)**	878,909	1,533,507
**Number of indetermined sites (Ns)**	340,936	1,168,836
**GC content (%)**	48.9	48.9
**Number of genes**	9,153	8,969
**Mean gene length (nucleotides)**	1,387	1,366
**Percentage of genes with introns**	67%	67%
**Mean number of exons per gene**	2.66	2.68

### Sexual locus, nitrate utilization and iron uptake

As in many other ascomycetous fungi, no teleomorfic form or sexual reproduction cycle has been described for *P. digitatum*. We nevertheless found a conserved alpha-box mating-type protein (MAT1) locus in both strains (Additional file 1: Figure S1) in an area of conserved synteny with other *Penicillium* and *Aspergillus* species suggesting the possibility of sexual reproduction in these species. In addition, *P. digitatum* shows the organization typical of heterothallic Pezizomycotina, with the presence of a single MAT locus [13].

**Figure 1:**
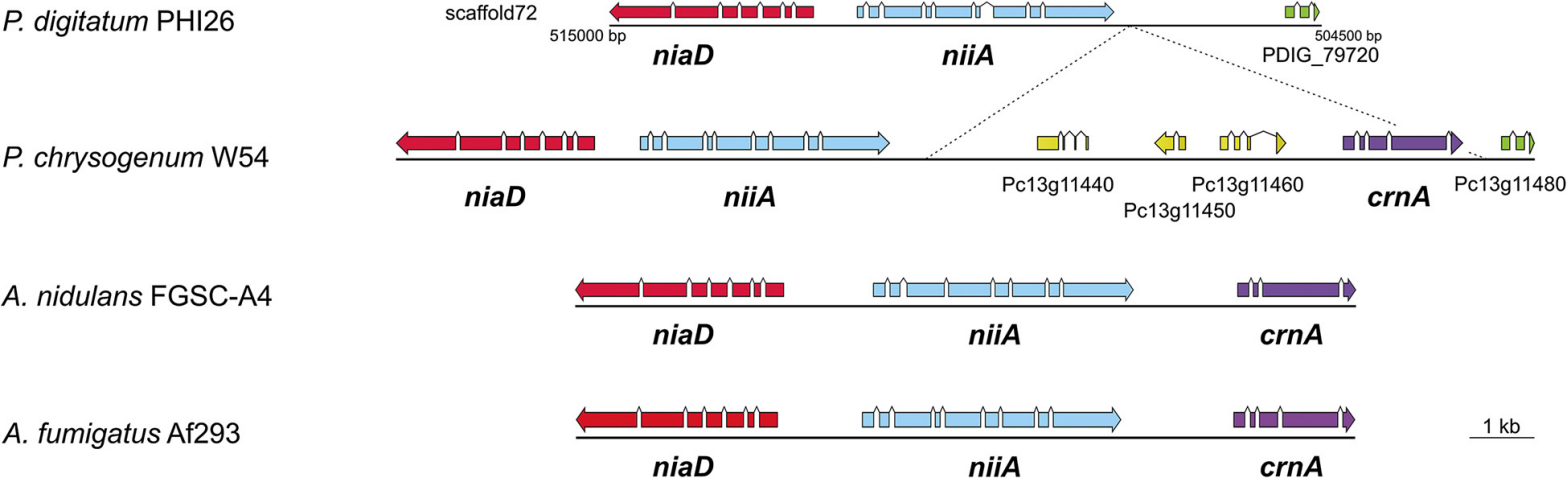
**Comparison of *cnrA-niiA-niaD *gene cluster among different fungal species.** Genomic context of the gene cluster encoding nitrate reductase (NiaD), nitrite reductase (NiiA) and nitrate permease (CnrA) in *P. digitatum* PHI26, *P. chrysogenum* Wisconsin 54, *A. nidulans* FGSC-A4 and *A. fumigatus* Af293. Dashed lines indicate the loss of *P. chrysogenum* crnA and adjacent genes in *P. digitatum* PHI26. Homologous genes are indicated with arrows of the same color.

*P. digitatum* is unique among *Penicillium* species in its inability to use nitrate as sole nitrogen source [14]. This pathway is important for other filamentous fungi that use it to cope with situations in which the main sources of nitrogen such as ammonium, glutamate or glutamine are scarce. In the model fungus *A. nidulans* the nitrate assimilation pathway comprises three components: a nitrate-specific transporter (CrnA), a nitrate reductase (NiaD) and a nitrite reductase (NiiA) [15], the encoding genes of which are clustered in the genome (Figure 1). In addition, in this species, nitrogen metabolism is controlled by both global (AreA) and pathway-specific (NirA) transcriptional regulators [16]. We searched the conceptual translation of the *P. digitatum* genome for the presence of homologs of the five above-mentioned *A. nidulans* proteins and found homologs to AreA, NirA, NiaD and NiiA, the latter two being encoded by contiguous genes. However, *P. digitatum* lacks a homolog for the nitrate transporter CrnA (Figure 1). This is not only supported by the absence of this gene in both assembled *P. digitatum* genomes but also by the complete absence of *P. digitatum* reads (assembled or unassembled) mapping to the *P. chrysogenum* crnA locus at low stringency. The cluster formed by *niaD-niiA-crnA* and the surrounding genes is highly conserved among *Aspergillus* species. However, *P. chrysogenum* contains three putative open reading frames (ORFs) between *niiA* and *crnA*. *P. digitatum* shares the same basic genome organization as *P. chrysogenum* with the exception that it lacks *crnA* and the three ORFs that interrupt the nitrate assimilation cluster. This indicates that this region has suffered important re-arrangements in the *Penicillium* lineage, including the loss of the nitrate transporter in *P. digitatum*, which would explain the inability of this species to use nitrate. A comparison of the rates of non-synonymous vs synonymous codon substitutions (dN/dS) between *P. chrysogenum* and *P. digitatum* in the nitrate gene cluster shows values compatible with purifying selection (0.36 for *niiA* and 0.48 for *niaD*), and similar to the average value for orthologous genes in these two species (0.47). This finding suggests the recent loss of *crnA* or, alternatively, that *niiA* and *niaD* may still be functional in the absence of *crnA.*

**Figure 2:**
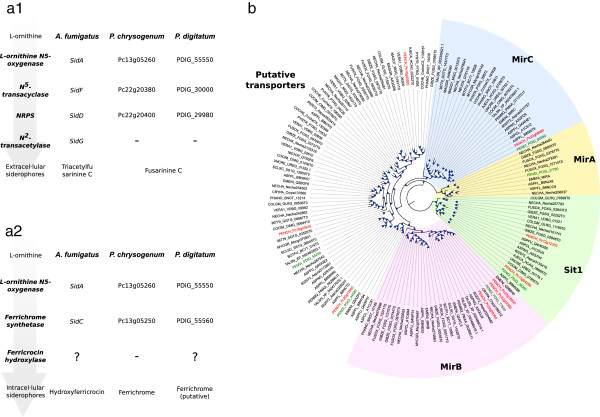
**Siderophore biosynthesis. a**) List of proteins involved in the synthesis of siderophores in the three species: *A. fumigatus, P. chrysogenum* and *P. digitatum*. **a1**) represents the synthesis of fusarinine C and **a2**) represents the synthesis of ferrichrome. **b**) Phylogenetic tree of the siderophore transporters in the 28 species used in the phylome. Colored regions represent homologs to the transporters MirC (blue), MirA (yellow), Sit1 (green) and MirB (red). Protein names colored in red represent *P. chrysogenum* proteins while the ones colored in green belong to *P. digitatum* proteins.

Iron uptake is another important aspect of the biology of this species. Like other filamentous fungi, *P. digitatum* is able to synthesize siderophores. Similar to other *Aspergillus* and *Penicillium* species it contains the genes necessary to synthesize the extracellular siderophore fusarinine C, namely the homologs to *sidA, sidF and sidD* (Figure 2a), though it lacks *sidG* which in *Aspergillus fumigatus* encodes a protein that catalizes the conversion of fusarinine C to the related siderophore triacetylfusarinine C. Like *P. chrysogenum*, *P. digitatum* is able to synthesize ferrichrome instead of ferricrocin as described in other *Aspergillus* species [17]. Additionally, *P. digitatum*’s genome encodes six putative siderophore transporters, two of which are surprisingly absent from closely related species such as *P. chrysogenum* (Figure 2b). One of these missing genes is *mirA,* which in *A. nidulans* is involved in the transport of the bacterial siderophore enterobactin [18]. In *P. digitatum mirA* is contiguous with *estA*, which in *A. nidulans* is thought to be involved in the hydrolysis of enterobactin [19]. The proximity of the two genes in *P. digitatum* supports the existence of a functional link between the proteins they encode. Conversely, four of the putative siderophore transporters that are found in *P. chrysogenum* lack homologs in *P. digitatum*. Other genomic and physiological features of *P. digitatum*, as well as their comparison with other sequenced Pezizomycotina are discussed below.

### Comparison of the two sequenced strains

The two sequenced strains are highly similar with pairs of orthologs sharing on average 99.96% identity at the protein level (99.95%, at the nucleotide level). This similarity extends to the chromosomal organization between the two strains, with 99.8% of neighboring gene pairs being conserved. We could only detect two putative chromosomal re-arrangements (Additional file 1: Figure S2). When aligning the reads of one strain to the other strain several coding regions remained unmapped suggesting that they could have been either lost or gained specifically in only one of the strains. More specifically, two genes were unique for strain Pd1 and eight for PHI26 (Additional file 1: Table S2), the two unique sequences in the resistant strain being contiguous in the assembly. The differences in the number of predicted genes for the two strains (Table 1) are actually larger than those shown in Additional file 1: Table S2. This is mostly due to genes not predicted in Pd1 as a consequence of assembly gaps, since 52% of the PHI26-unique genes mapped to regions containing Ns and 48% appeared truncated in the Pd1 assembly. The prediction of unique genes based on the mapping of reads from the other strain (Additional file 1: Table S2) is independent of differences in the assembly or gene prediction quality and thus should be considered to be more accurate.

Few single nucleotide polymorphisms (SNPs) were noted between the two strains. In total 1,441 high confidence SNPs were found, of which 553 affected coding sequences. Remarkably, there is an excess of non-synonymous (358) vs synonymous (195) SNPs, suggesting the possibility that part of the differences are the result of positive selection. Among the 22 genes with more than one non-synonymous SNPs (Additional file 1: Table S3) we found one possibly having a regulatory function (PDIG_27530) and two putative membrane transporters (PDIG_44900 and PDIG_41990) that could be involved in the extrusion of toxic compounds. In this regard it is interesting to note that *P. digitatum* Pd1 is significantly more resistant than PHI26 to several additional fungicides imazalil and thiabendazole (see below).

Among the detected SNPs we confirmed the presence of the Phe-to-Tyr mutation at codon 200 in the β-tubulin gene which has been associated with thiabendazole resistance in *P. digitatum *[8]. However, a second mutation affecting this gene, and related to benomyl resistance, was not found in either strain [20]. Of particular note, we found two novel mutations in Pd1 both of which cause coding sequence truncations, one in a gene (PDIP_45840) encoding a putative member of a phosphotransferase family (APH, PFAM: PF01636) implicated in antibiotic resistance in bacteria [21], and another in a gene (PDIG_06580) predicted to encode a putative hydrolase. The possible involvement in fungicide resistance of these mutations and some of the remaining differences between the two strains will require experimental validation. Going one step in this direction, we specifically tested for this in the two abovementioned Pd1-specific genes by constructing the corresponding knock-out strains and exposing them to a battery of six widely-used fungicides (See Materials and Methods and Additional file 1). Neither of the two deleted strains showed significant differences in either fungicide resistance or their pathogenicity towards citrus fruits with respect to the wild-type Pd1 control or an ectopic (non-deleted) transformant (Additional file 1: Figures S3 to S6, and Additional file 1: Table S4). We thus discount a direct involvement of these two genes in resistance to the six fungicides tested.

In addition to the differences already commented, our analyses also confirm the presence of a 199 bp insertion in the promoter of the *cyp51B* gene in Pd1 that has been shown to confer resistance to imazalil by increasing gene expression [22]. No other variations were found in any of the three *cyp51* genes described that could account for the differential susceptibility to imazalil observed between isolates Pd1 and PHI26. Notably, this 199 bp motif is represented 9 times in the Pd1 genome and 12 times in PHI26. The locations of eight of these motifs are common between the strains but all except the resistance-associated insertion fall distant from protein-coding genes. Comparison between the two strains revealed that the motifs absent in Pd1 correspond to genomic regions that are either unassembled or unresolved for this strain. Remarkably, the insertion site of the 199 bp motif in the *cyp51B* promoter is identical to that found in two other imazalil-resistant isolates from China [22]. Given the few differences found between the two sequenced Spanish strains and the observation that only a fraction of them are directly related to the resistance phenotype indicates that the appearance of fungicide resistance in *P. digitatum* is likely to involve few genomic mutations.

The high degree of genomic conservation also extends to the mitochondrial genomes of Pd1 and PHI26. They exhibit very few differences and all involve small variations in the lengths of polynucleotide tracks, a phenomenon attributable to known sequencing error biases in the 454 technology [23]. It is thus highly likely that Pd1 and PHI26 have identical mitochondrial DNA sequences. We next compared this sequence to the recently reported mitochondrial sequence from the Chinese isolate pd01 [24], and this also turned out to be identical. This indicates a remarkable conservation of mitochondrial DNA sequences in distant isolates, and is suggestive of a recent expansion of *P. digitatum* around the globe. The cultivation of citrus has its origin in Southeast Asia, and, while its presence in Southern Europe can be dated back to the times of the Roman Empire, intensive production and massive worldwide trade only developed as recently as the 1940s [25]. Our results indicate that the *P. digitatum* strains isolated in Spain diverged from the Chinese strain very recently and argue in favor of a recent expansion of this pathogenic species, probably coinciding with the initiation of intensive agricultural practices and worldwide trade.

### Comparison across species

**Figure 3:**
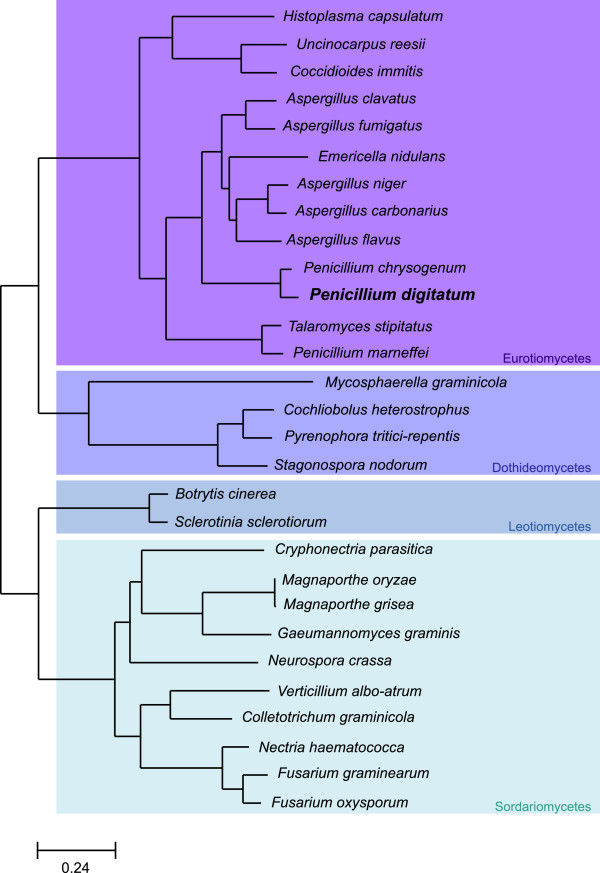
**Species tree showing the phylogenetic position of *Penicillium digitatum *across other 28 Pezizomycotina species.** The four Pezizomycotina groups for which organisms have been sequenced are represented in different colors. The tree was reconstructed using a concatenation method based on 592 genes. All nodes displayed a bootstrap support of 100%, based on 100 alignment replicates.

**Figure 4:**
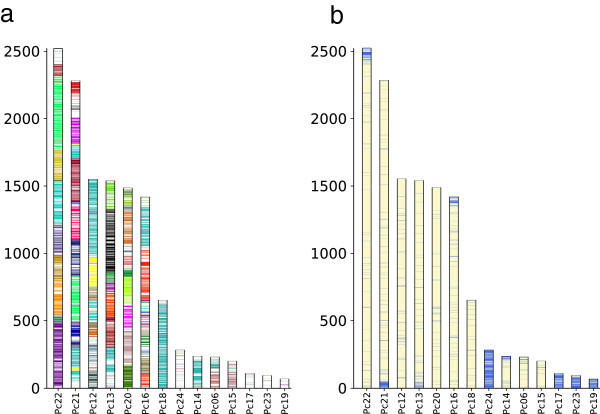
**Shared and unique genomic regions in *Penicillium chrysogenum. *a**) Supercontigs defined in *P. chrysogenum* colored according to whether genes have correspondent orthologs in *P. digitatum*. Supercontigs are represented by the sum of their genes, with one gene being one strip. Regions of the same color match to the same scaffold in *P. digitatum*. White regions represent lack of orthologs in *P. digitatum***b**) *P. chrysogenum* supercontigs as in **a**) but now blue colored lines representing location of paralogs of the genes located in the four missing supercontigs (Pc24, Pc23, Pc17 and Pc19).

We next compared the *P. digitatum* genome to those of 28 other sequenced Pezizomycotina genomes including 12 other Eurotiomycetes, 10 Sordariomycetes, 4 Dothideomycetes, and 2 Leotiomycetes (Additional file 1: Table S5). We did so by sequence comparisons and by reconstructing the complete collection of phylogenetic trees of each *P. digitatum* protein and their homologs in other species (i.e. a phylome). This enabled us to derive a complete catalogue of orthology and paralogy relationships among the species considered based on phylogenetic evidence [11]. All the alignments and gene trees are publicly available in phylomeDB (http://www.phylomedb.org, [26]). In addition we reconstructed a species phylogeny based on the concatenation of 592 conserved orthologs (Figure 3). This phylogeny is highly supported and generally congruent, for the shared leaves, with previously published phylogenies of Pezizomycotina [10]. In addition, this topology is the most parsimonious in terms of inferred duplications in the 7,920 gene trees present in the *P. digitatum* phylome. The closest species related to *P. digitatum* with a completely sequenced genome is *P. chrysogenum *[17]. Differences in gene content are noticeable, with the latter genome containing over 3,500 genes more (12,789 vs 9,153). Orthology predictions revealed that only 7,123 (55%) genes in *P. chrysogenum* had an ortholog in *P. digitatum.* Conversely, 7,107 (86%) *P. digitatum* genes have an ortholog in *P. chrysogenum*. Interestingly, and despite the large differences in the total number of genes between *P. digitatum* and *P. chrysogenum*, both genomes have similar representations of the different functional classes. Indeed, only small differences in the representation of COG functional categories were found (see Additional file 1: Figure S7). A comparison of Gene Ontology (GO) terms [27] however, showed two biological processes to be enriched among those genes present in *P. chrysogenum* but absent from *P. digitatum*: “transmembrane transport”, and “DNA-dependent regulation of transcription”. This indicates that the streamlining of the *P. digitatum* genome has similarly affected all functional classes except two, which are likely related to the broader niche distribution and physiological flexibility of *P. chrysogenum*. Genes lacking orthologs in *P. digitatum* are not distributed uniformly across the *P. chrysogenum* assembly (Figure 4a), and are confined to supercontigs Pc24, Pc23, Pc19 or Pc17. Remarkably our data also show that the 543 genes in these supercontigs do not seem to have homologs in *Aspergillus* species either, suggesting that they were gained specifically in *P. chrysogenum*. Exploration of the chromosomal locations of blast hits and phylogenetic trees for the genes in these supercontigs reveals that a large percentage (75%) of the protein-coding genes of the latter seem to have emerged from expansions of gene families that are present in other chromosomes (Figure 4b). Thus, recruitment from larger chromosomes and gene expansion, rather than direct acquisition through horizontal gene transfer seems to be the mechanism behind the origin of these chromosomal regions in *P. chrysogenum*. Notably, we found that 50–60% of the proteins in *P. chrysogenum* annotated as having an “integrase core” or “reverse transcriptase” domains (see Additional file 1: Table S6) were present in these unique contigs pointing to a possible retrotransposon-mediated mechanism of gene expansion. Other domains specifically enriched in these contigs include the HAT dimerization domain (10 out of 13 domain-containing proteins are encoded in the unique contigs), BED Zink fingers (2 out of 3) and the CP2 transcription factor (2 out of 4).

Based on the phylome analysis we found that 843 *P. digitatum* proteins lack homologs in the closely-related but non-pathogenic species *P. chrysogenum*. Of these, 97 have homologs exclusively in other plant pathogenic species thus highlighting them as good candidates for possible roles in virulence (Additional file 1: Table S7). Other genes in *P. digitatum* have homologs in a limited number of fungal species while having widespread bacterial homologs, a pattern that could be indicative of Horizontal Gene Transfer (HGT). Previous analyses [28] have found that genomes from *Pezizomycotina* species are particularly enriched for genes acquired from prokaryotes. We have applied the same strategy to find genes of putatively prokaryotic origin in *P. digitatum*. Four genes fulfilled our criteria (Additional file 1: Table S8). Notably, one of these (PDIG_81140) was identified as having homologs in plant pathogenic fungi but not in the non-pathogenic *Penicillium*, and also a homolog in a bacterial family (DEC1) that has been associated with virulence in maize infections and the synthesis of T-toxin in *Cochliobolus heterostrophus *[29]. None of the putatively transferred genes identified here in *P. digitatum* correspond to genes that have been described as HGT in *P. chrysogenum*, among which the penicillin gene cluster is included (see below). Another relevant HGT described in some fungi [28], but absent in *P. digitatum*, is arsenate reductase (arsC) which in *P. chrysogenum *[17] forms part of an arsenate resistance cluster. Remarkably, we also identified the absence of homologs to the alternative arsenate reductases present in *Saccharomyces cerevisiae* (Acr2p) or in other distantly-related fungi [28]. This would suggest that *P. digitatum* may be vulnerable to arsenic, or alternatively, that this species may be using another detoxification system not previously described in fungi. We next focused on five particular aspects of *P. digitatum* genomic content that are likely to provide hints for understanding this organism’s pathogenic lifestyle and constitute interesting targets for future research: namely i) secondary metabolism gene clusters, ii) the prediction of putative small cysteine-rich peptides (CRPs), iii) homologs of known effector proteins iv) carbohydrate active enzymes (CAZy), and finally iv) the repertoire of secreted proteins among which we expect to find those that potentially interact with the host and are involved in invasive growth of the fungus.

### Secondary metabolism

**Figure 5:**
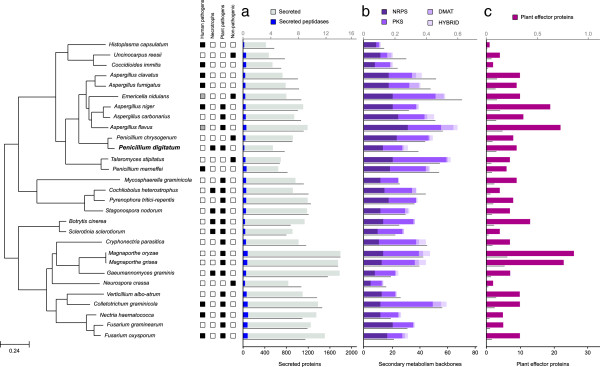
**Summary of several genome characteristics discussed in the paper.** The tree on the left represents the evolution of the 29 fungal species included in the study. The squares to the right of the tree represent the life-style of the different organisms. Black colored boxes mark the life-style of a given organism as described in the literature. Grey colored boxes indicate that the life-style has been seen in the species, but is uncommon. **a**) Number of predicted secreted proteins. The darker blue squares represent those secreted proteins that have also been predicted as peptidases. Bars are referred to the scale shown at the bottom of the graph. The thin, grey line represents the percentage of genes in each given genome coding for secreted proteins as referred to the upper-scale. **b**) Number of backbone genes involved in the synthesis of secondary metabolism clusters, referred to the scale shown at the bottom of the graph. They are divided in four groups belonging to polyketide synthases (PKS), nonribosomal peptide synthases (NRPS), dimethyl allyl tryptophan synthases (DMATs) and PKS-NRPS hybrids. The structure of the graph is the same as in A. **c**) Number of genes that have homologs of effector proteins involved in plant pathogenesis. The structure of the graph is the same as in graph A.

Many important characteristics of fungi are the result of secondary metabolic activities, the genes of which tend to be organized in clusters. The discovery of these genes and the study of their evolution is a topic of increasing interest [30]. Several *Penicillium* species are sources of compounds or enzymatic activities that have important clinical or biotechnological applications [31]. These include the well-known antibiotic penicillin produced by *P. chrysogenum* and the antifungal and potential cancer chemotherapeutic agent griseofulvin produced by *P. griseofulvum*, as well as the capacity shown by diverse *Penicillium* species for the degradation of several xenobiotics [32]. We detected 24 clusters in *P. digitatum* PHI26 containing, among others, 31 backbone genes*,* of which 13 were nonribosomal peptide synthases (NRPSs), 14 polyketide synthases (PKS), one prenyltransferase (DMATSs) and three NRPS/PKS hybrids (Additional file 1: Table S9). While fewer secondary metabolic clusters were found compared to *P. chrysogenum* (41), the difference is less when the relative sizes of the two genomes are taken into consideration (Figure 5). Notably, the penicillin biosynthetic cluster is absent in *P. digitatum*, indicating the inability of this species to produce this potent antibiotic and suggesting that it may possess the ability to synthesize alternative antibiotics. Similarly, while other *Penicillium* species are able to synthesize cyclopiazonic acid [31], *P. digitatum* is apparently unable to do so. Indeed, a search of the *P. digitatum* genome failed to identify a cyclopiazonic acid gene cluster (described in *Aspergillus flavus *[33]) or the presence of genes encoding additional enzymes such as MoxA or Ord1 required for the production of this metabolite. We were also unable to detect the presence of a putative cluster for the biosynthesis of patulin (described in *Aspergillus clavatus *[34]) which is known to be a potent mycotoxin in *Penicillium expansum*[35], another postharvest pathogen closely related to *P. digitatum*. Orthologs were nevertheless identified for seven of the fifteen patulin cluster members (*patB, patC, patD, patF, patG, patJ, and patL*), six of which are clustered (Additional file 1: Figure S8). Interestingly, a putative truncated homolog of *patI* appears in the intergenic region between *patJ* and *patL*. Two additional genes (*patH* and *patK*) are located in a different scaffold separated by three genes. The presence of a truncated *patI* pseudogene, the loss of the other components and the absence of backbone genes around the cluster, suggest that these genes do not function in the patulin biosynthetic pathway. Consistent with this interpretation, we found ratios of non-synonymous versus synonymous substitutions (dN/dS) close to 1 in most of the genes in the cluster, which is consistent with relaxation of the selective pressure (Additional file 1: Table S10). Other clusters present in other fungal species but apparently absent in *P. digitatum* include, but are not limited to, griseofulvin, viridicatumtoxin, fumonisin, clavines, aflatoxins, sterigmatocystin, citrinin, ergot alkaloid, lovastatin and paxilline. We have however identified the melanine and the tryptoquialanine synthesis clusters, the latter previously described in *Penicillium aethiopicum *[36] and absent in *P. chrysogenum*.

### Small, cysteine-rich peptides and effector proteins

Several fungi are known to secrete small cysteine-rich proteins (CRPs) that exhibit diverse biological functions including adherence [37], virulence [38,39] or antimicrobiosis [40,41]. Some CRPs are virulence effectors that show carbohydrate binding activity that interferes with host recognition of the pathogen [42]. It has been suggested that antimicrobial proteins could become extremely useful in medicine and agriculture to treat pathogenic infections in the near future [41,43]. Several antifungal CRPs exhibiting antimicrobial activity have been characterized from ascomycetous fungal species from the genera *Aspergillus* and *Penicillium*[40]. These cationic proteins share the previously described γ-motif common to small antimicrobial proteins and peptides [44]. Two distinct antifungal CRPs have been isolated and characterized from cultures of *P. chrysogenum *[45,46]. Among the predicted proteins in *P. digitatum* we found a CRP (PDIG_68840) showing 91% amino acid identity with the Pc12g08290 gene from *P. chrysogenum* which corresponds to one of the previously reported proteins [46]. Remarkably, the ortholog to Pc24g00380 (the most studied antifungal protein from this fungus) is absent in *P. digitatum* as it is located in the Pc24 supercontig which is virtually devoid of *P. digitatum* orthologs. We identified two additional putative CRPs in *P. digitatum* (PDIG_31210 and PDIG_70730) by performing profile-searches with CRP motifs identified in plants [47]. Due to their short length, CRPs are likely to be under-predicted in the annotation of sequenced genomes. This prompted us to extend the genome annotation with a specific search (see Materials and Methods) for additional small CRPs that contain at least four cysteine residues, display similarities to known CRPs, harbor the γ-motif and/or have a high content of cationic residues typical of antimicrobial proteins, under the assumption that these might have been under-predicted in the initial annotation (see Materials and Methods). We found 73 additional ORFs that potentially would code for CRPs, ranging in size from 23 to 146 residues (Additional file 1: Table S11). Two of these putative CRPs (Pdigorf_18868 and Pdigorf_93050) share the γ-motif, making them good candidates for having antifungal activity.

We next searched the pathogen-host interaction database [48] for *P. digitatum* proteins homologous to effector proteins from other pathogenic fungi [38]. These include not only CRPs but also other types of proteins that can modulate host physiology. Again, *P. digitatum* encodes a smaller number of homologs of known effector proteins compared to *P. chrysogenum*, but abundances relative to the total proteome are comparable (Figure 5). We found eight *P. digitatum* homologs to Ace1 of *Magnaporte grisea*, six to HopI1 (*Pseudomonas syringae*), and single homologs to Npp1 (*Hyaloperonospora parasitica*), Gip1 and Gip2 (*Phytophtora infestans*). Additionally, five proteins in *P. digitatum* contain the LysM domain (Additional file 1: Table S12) of which two have no orthologs in *P. chrysogenum* and are closely related to *A. nidulans* and *Aspergillus carbonarius* instead. Of note, the fraction of proteins with homologs in the known effector proteins dataset is roughly similar (0.01–0.2%) across all the pezyzomycotina genomes analyzed regardless of their phytopathogenicity. This result illustrates the limitations of using mere homology to predict the functional roles of proteins.

### Carbohydrate active enzymes (CAZymes)

**Figure 6:**
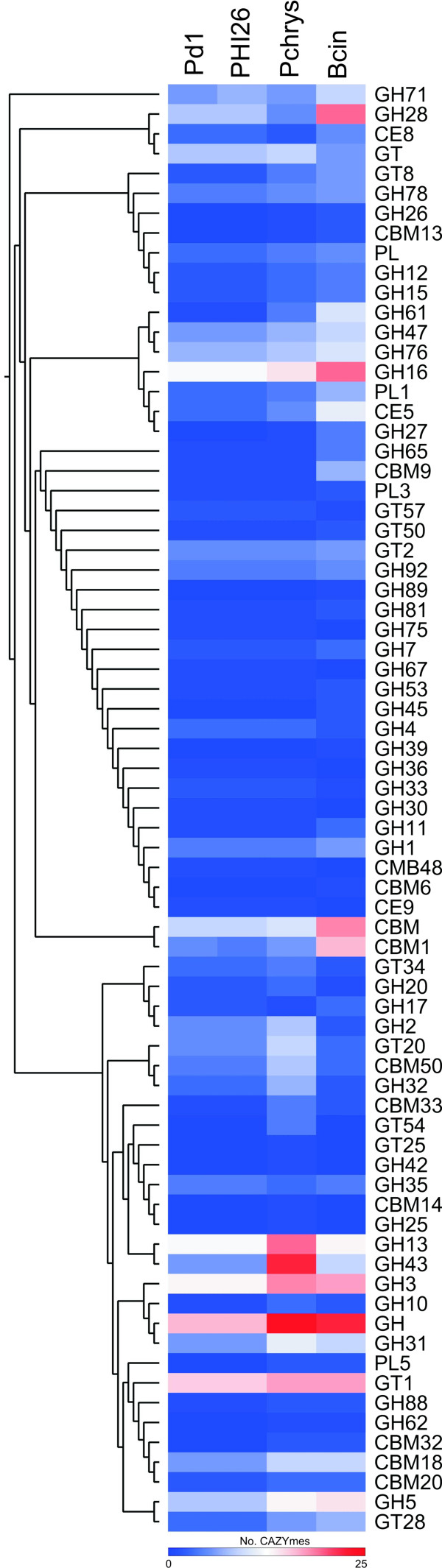
**Comparison of the CAZyme repertories identified in the genome of selected fungi using hierarchical clustering.** Fungal genomes analyzed: *P. digitatum* Pd1, *P. digitatum* PHI26, *P. chrysogenum* and *Botrytis cinerea* B05.10. Enzyme families are represented by their classes (GH: glycoside hydrolases, GT: glycosyltransferases, PL: polysaccharide lyases, CE: carbohydrate esterases, and CBM: chitin binding modules) and family number according to the carbohydrate-active enzyme database. Abundance of the different enzymes within a family is represented by a color scale from 0 (dark blue) to 25 occurrences (dark red) per species.

Plant cell wall carbohydrate components form a complex network of cellulose, hemicellulose and pectin. These polysaccharides, together with others not derived from plants, are targets of the carbohydrate-active enzymes (also known as CAZymes) that degrade them into simple monomers that can be used as nutrients [49]. A search for InterPro signatures present in CAZyme families identified 275 putative CAZyme genes (Figure 6 and Additional file 1: Table S13). This number is one of the smallest reported for ascomycetes [50]. However, in terms of relative abundance this number is comparable to the 383 CAZymes present in *P. chrysogenum*. Among *P. digitatum* CAZymes, 49 have clear assignments to either glycoside hydrolases (GH), carbohydrate esterases (CE) or polysaccharide lyases (PL) involved in fungal cell wall degradation, a number close to the 52 found in *P. chrysogenum*. In contrast, a larger difference was observed in the number of enzymes involved in plant cell wall degradation: 49 in *P. digitatum* as compared to 72 in *P. chrysogenum*. Most of this difference can be attributed to GH families 10, 31, 43 and 61, which account for 40 enzymes in *P. chrysogenum* and only 14 in *P. digitatum*. Enzymes in these families are mostly related to the degradation of celullose (endoglucanases) and hemicellulose (xylanases, xylosidades, arabinofuranosidases). Interestingly, two CAZy families involved in pectin degradation are enriched in *P. digitatum* compared to *P. chrysogenum. P. digitatum* contains eight polygalacturonases belonging to familiy GH28, and three pectinesterases included in family CE8, whereas *P. chrysogenum* has five and two proteins in these two families, respectively. Given that *P. digitatum* is a phytopathogen, whereas *P. chrysogenum* is not, these differences could provide hints to help identify the CAZymes relevant for citrus fruit infection in *P. digitatum*.

### Secretome

Fungi use secreted enzymes to break-down biopolymers the resulting products of which are then transported inside cells for further metabolic processing, and in phytopathogenic fungi secreted proteins constitute the first line of interaction with the host and play important roles in virulence [51-53]. Since secreted fungal enzymes have found a wide range of applications in the food, feed, pulp and paper, bioethanol and textile industries there is a growing interest in deciphering the complement of secreted proteins in sequenced genomes (i.e. the secretome). Using an *in silico* pipeline (Methods) we predicted 552 secreted proteins, of which 133 (24%) code for various hydrolases such as proteases, lipases or nucleases (Additional file 1: Table S14) Among the pezizomycotina species considered, *P. digitatum* has the second smallest predicted proteome after *Uncinocarpus reesii* (Additional file 1: Table S5). Similarly, *P. digitatum* also has one of the smallest secretomes comprising 552 proteins (6% of the proteome), which in relative terms is somewhat smaller than the secretome of *P. chrysogenum* (914 proteins, 7%) and the average 8% found in *Aspergillus* species, and much lower than the 14% of secreted proteins found in the *Magnaporthe* species (Figure 5c). That *P. digitatum* is a necrotroph and contains one of the smallest secretomes among analyzed fungi suggests that the absolute number of secreted proteins has no direct relationship with the lifestyle of a pathogen.

## Conclusion

Previous analyses comparing the genomic features of necrotrophic fungal pathogens [50] lacked the inclusion of a necrotrophic eurotiomycete. Our comparison of two *P. digitatum* genomes from strains that differ in their fungicide resistance traits with those of other sequenced pezizomycotina thus constitutes the first comprehensive genomic analysis of a necrotrophic organism within this important fungal clade that includes other plant pathogens such as those in the *Aspergillu*s genus. Our data suggest a recent global expansion of *P. digitatum*, consistent with the relatively modern origin of intensive cultivation and worldwide commercialization of citrus. In addition, our comparative analysis suggests the action of positive selection and that important differences in fungicide resistance seem to have been achieved through a small number of genomic changes. Our analyses also uncovered other strain-specific differences, the functional roles of which will be worth exploring. We show a striking reduction in gene content in *P. digitatum* compared to the close relative *P. chrysogenum*. Large regions in the *P. chrysogenum* genome, including entire supercontigs, are completely absent from *P. digitatum* and originated via large gene family expansions rather than through horizontal gene transfer. Although we could only detect enrichment of two functional classes among the genes lost in the *P. digitatum* lineage, detailed analysis revealed important physiological implications such as the loss of the ability to use nitrate as a nitrogen source. Other differences extend to secreted proteins and CAZyme-encoding genes, and may serve as a basis to understand the host-specificity of *P. digitatum*. With respect to pathogenicity determinants, and consistent with analysis of other phytopathogenic genomes [50], there seem to be no global differences that distinguish these pathogens from other pathogenic and non-pathogenic fungi. These findings reinforce the view that necrotrophic pathogenicity is a very complex trait that involves many genes that may vary in different lineages. Our data pave the way for a more systematic functional and experimental analysis of candidate genes that will eventually reveal the details of the complex interaction between *P. digitatum* and its host.

## Methods

### Fungal strains

*Penicillium digitatum* (Pers.:Fr.) Sacc. PHI26 and Pd1 strains have been used in this study. PHI26 was isolated from an infected orange [7] and Pd1 from an infected grapefruit [8], both in Valencia (Spain). Both strains have been deposited with the Spanish Type Culture Collection (CECT) with accession codes CECT20796 and CECT20795, respectively.

### RNA-Seq analysis

RNA-Seq data were generated from four different libraries: a cDNA library synthesized from *P. digitatum* spores collected after 7 days of growth in potato dextrose agar, and three libraries from *P. digitatum*-infected oranges at 12, 24, and 48 h post inoculation. Fruit inoculation and RNA extraction were conducted following procedures previously described [54]. cDNA libraries were generated using the MINT-Universal cDNA synthesis kit (Evrogen) following the manufacturer’s protocol. Sequencing was carried out using the Roche 454 GS FLX Titanium system. The raw data files are available in the Sequence read archive at the National Center for Biotechnology Information (NCBI), GenBank ID: SRA059533.

### Genomic DNA extraction

*P. digitatum* PHI26 and Pd1 (5 × 10^5^ conidia/mL) were grown in 500 mL Potato Dextrose Broth media (PDB, Difco) on a shaker at 180 rpm at 24°C for 4 days. Fungal mass was collected, dried and frozen in liquid nitrogen. DNA was extracted from 1 g of frozen mycelium essentially as previously described [55]. The final DNA preparation was incubated overnight at room temperature in 490 μL of TE buffer and 10 μL of DNase-free RNase (10 μg/mL), followed by phenol-chloroform extraction and isopropanol precipitation. Finally, DNA was resuspended in TE buffer. DNA quality was assessed through spectrophotometry and CHEF electrophoresis (data not shown).

### Genomic sequencing and assembly

Genome sequencing was performed at the CRG ultrasequencing facility (PHI26) and at LifeSquencing (Pd1) using Illumina HiSeq 2000 and Roche 454 GS FLX Titanium technologies, respectively. For the Illumina sequencing (PHI26) DNA was fragmented by nebulization to a size ~400-600 bp. After shearing, the ends of DNA fragments were blunted with T4 DNA polymerase and Klenow fragment (New England Biolabs). DNA was purified with a QIAquick PCR purification kit (Qiagen). Thereafter, 3^′^-adenylation was performed by incubation with dATP and 3^′^-5^′^-exo^−^ Klenow fragment (New England Biolabs). DNA was purified using MinElute spin columns (Qiagen) and double-stranded Illumina paired-end adapters were ligated to the DNA using rapid T4 DNA ligase (New England Biolabs). After another purification step, adapter-ligated fragments were enriched, and adapters were extended by selective amplification in an 18-cycle PCR reaction using Phusion DNA polymerase (Finnzymes). Libraries were quantified and loaded into Illumina flow-cells at concentrations of 7–20 pM (Genome Analyzer IIx; GA), or 1.4–1.75 pM (HiSeq 2000; HS). Cluster generation was performed in an Illumina cluster station (GA) or in a cBOT (HS). Sequence runs of 2 × 50 cycles were performed on the sequencing instruments. Base calling was performed using Illumina pipeline software. In case of multiplexed libraries, we used 4 bp internal indices (5^′^ indexed sequences). De-convolution was performed using the CASAVA software (Illumina). A total of 3.4 Gb of pair-end (350 insert size) and 2.6 Gb of mate-pairs (5 kb inserts), were produced. Reads were trimmed at the base with a Phred quality score lower than 10, or at the first undetermined base. Reads shorter than 31 bases (and their corresponding pairs) were removed. Reads were assembled using SOAPdenovo [56]. The best Kmer size was chosen based on optimization of the N50, assembly length, largest contig length, and number of contigs. Two suboptimal assemblies were consistent with the main results presented here. Gaps were then filled in using Gapcloser [57]. We exploited the similarity of the two sequenced strains and used OSLay [58] to create scaffolds. Length of gaps were controlled and contigs joined through large stretches (>10Kb) of Ns were separated.

For the Roche 454 sequencing (Pd1), a random “shotgun” genomic library was generated via fragmentation of 10 μg of *P. digitatum* Pd1 genomic DNA employing the GS FLX Titanium General Library Preparation Kit following the manufacturer’s recommendations (454 Life Sciences, Branford, CT). Briefly, DNA was randomly sheared via nebulization and double stranded DNA adaptors were blunt-ligated to fragment ends following post-electrophoresis agarose gel excision of the 500–800 bp fraction. The final single-stranded DNA library was isolated via streptavidin bead binding to biotinylated adaptors followed by alkaline treatment. The library was quantitated via fluorometry using Quant-iT RiboGreen reagent (Invitrogen, Carlsbad, CA) prior to emulsion PCR amplification. Subsequently, a paired end library was constructed. Briefly, 30 μg of double stranded genomic DNA was randomly fragmented via hydrodynamic shearing to an average size of 15,000 or 20,000 bp using the HydroShear apparatus (Genomic Solutions, Ann Arbor, MI). Fragment ends were blunt ended via T4 DNA polymerase and T4 polynucleotide kinase (Roche Applied Science, Indianapolis, IN) treatment, and a double-stranded DNA adaptor (containing a loxP recognition site and internal biotin moiety) was blunt-ligated to DNA ends. Library fragments in the desired size range were isolated from excised gel fragments via electroelution following overnight 0.5% agarose gel electrophoresis. Nicks present at the 3^′^-junctions of each of the adaptors and the library fragment were filled in by the strand-displacement activity of the Bst DNA polymerase (New England BioLabs, Ipswich, MA). Linear library molecules were circularized via Cre recombinase (New England BioLabs, Ipswich, MA) excision. These molecules along with 1 μg carrier DNA were randomly fragmented via nebulization to an average size of approximately 450–500 bp. Fragment ends were blunted and desired library fragments (linker flanked by original DNA fragment ends) were selected via the biotinylated linker, using 1 Dynal M-270 magnetic streptavidin beads (Invitrogen, Carlsbad, CA). Double-stranded DNA adaptors were ligated to blunt fragment ends. The resulting library was amplified using 20 cycles of PCR and purified with AMPure beads (Agencourt Bioscience, Beverly, MA, USA) to remove small DNA fragments and amplification primers. Single stranded DNA library was isolated via Dynal M-270 bead binding followed by alkaline treatment and quantitation using Quant-iT RiboGreen (Invitrogen, Carlsbad, CA) prior to emulsion PCR amplification. Genomic shotgun library molecules were clonally amplified via emulsion PCR following manufacturer’s recommendations employing the GS FLX Titanium LV emPCR Kit (454 Life Sciences, Branford, CT). Paired end library was handled similarly, with the exception that 30 μL of amplification primer was used per Large Volume Emulsion cup. Following amplification, emPCR reactions were collected, and emulsions broken according to the manufacturer’s protocols. Beads containing sufficient copies of clonally amplified library fragments were selected via the specified enrichment procedure and counted with a Multisizer 3 Coulter Counter (Beckman Coulter, Fullerton, CA) prior to sequencing. Following emulsion PCR enrichment, beads were deposited into the wells of a Titanium Series PicoTiterPlate device and 454 Sequencing was performed using the GS FLX instrument according to the manufacturer’s recommendations (454 Life Sciences, Branford, CT). Image analysis, signal processing and base calling were performed using supplied system software. Standard Flowgram Format (sff) files output from base calling were employed in subsequent genome assembly. The assembly of 454 reads was performed with Newbler (454-Roche).

### Genome annotation

We used a genome annotation pipeline similar to that used for the closely-related *P. chrysogenum* genome [17]. In brief, we combined predictions from three alternative methods (Additional file 1: Figure S9): i) a first annotation was obtained using Augustus trained with *A. nidulans* files. RNA-Seq data from *P. digitatum* Pd1 in different growth conditions were incorporated in the predictions as hints. ii) a second prediction was done using JIGSAW [59] in order to unify the predictions of several predicting programs and methods which included a) Augustus [60] (without RNA-Seq data), b) GeneID [61], and c) SNAP [62], which were run using training files based on either *P. chrysogenum* or *A. nidulans*, depending on availability; d) a custom python script was used to detect Open Reading Frames (ORFs) using the universal genetic code, e) homology data obtained from Blast searches for each protein encoded in the three sequenced *Penicillium* species (*P. chrysogenum, Penicillium marneffei* and *Talaromyces stipitatus*) and several other Pezizomycotina species (*A. nidulans, A. flavus, Neurospora crassa, Histoplasma capsulatum* and *Gibberella zeae*) run against the *P. digitatum* genomes; f) GMAP [63] was used to map the genes encoded in *P. chrysogenum* to the newly sequenced *P. digitatum* genomes. iii) A third prediction was run using GeneWise [64] to search for all the proteins encoded in several fungal genomes (*P. chrysogenum, P. marneffei, T. stipitatus, A. nidulans, Fusarium oxysporum, A. flavus, N. crassa, G. zeae, H. capsulatum*) and the set of revised proteins found in Uniprot (as of June 2011). An initial Blast was performed to locate the contig in which a protein was potentially present, then GeneWise was run to establish its most likely position within the contig. Hits with a bit score over 50 were revised and extended in case of lack of starting or stop codon. If no internal stop codons were found in the final gene, this was added to the annotation. The final annotation was build using the first prediction as a starting point. Proteins detected in the second and third annotations where added when there was no overlapping with the initial set. The final predicted gene set included 9,153 and 8,969 genes for PHI26 and Pd1, respectively. Pfam domains [65] were searched using HMMER v3 with default values. GO terms and E.C. codes and mapping to KEGG [66] pathways were transferred from several of the fungal genomes when a one-to-one orthology relationship existed. A total of 6403 (70%) proteins contained at least some kind of annotation. Tansposons and repetitive elements were predicted with RepeatMasker [9].

### Secretome prediction

The subcellular location of each protein in the 29 genomes used in this study was predicted using PSORT [67]. Additionally, all proteomes were scanned to look for proteins containing a signal peptide using SignalP [68]. These proteins were then scanned for the presence of transmembrane domains using TMHMM [69]. 552 proteins in PHI26 and 547 proteins in Pd1 were considered as secreted proteins.

### Prediction of small cystein - rich proteins (CRP)

Prediction of CRPs was based according to their expected sequence characteristics [70]. Known proteins and predicted open reading frames (ORFs) of small (20 to 150 amino acids) size, which presented a predicted signal peptide from the secretory pathway and which contained at least four cysteins were pre-selected. This resulted in 6,961 short proteins in the seven genomes analyzed: *P. digitatum* (both strains), *P. chrysogenum, A. clavatus, A. fumigatus, A. flavus, Aspergillus niger.* Subsequently, HMM-derived profiles of 516 plant CRP motives described [47] were used to search the above mentioned collection of proteins using HMMER3 [71]. Hits were considered if they had an e-value below 0.1. Peptides hit by the same CRP motif and showing a level of identity greater than 20% were grouped and aligned to derive a new HMM profile, which was then used as bait in a novel search. This process was repeated iteratively until no further peptides were added. This procedure yielded 73 putative CRPs.

### Comparative analyses

#### Phylome reconstruction

The phylome, meaning the complete collection of phylogenetic trees for each gene in a genome, was reconstructed for the genomes of *P. digitatum* (strain PHI26) and of *P. chrysogenum*. The phylome was reconstructed using 28 other Pezizomycotina species. Considering the high degree of similarity between the two *P. digitatum* strains only the PHI26 strain was used in the phylomes. The phylome was reconstructed using an automated pipeline previously described in [26]. Briefly, for each protein in a genome a Smith-Waterman search was performed against the fungal proteome database (Additional file 1: Table S5). Results were filtered using an e-value < 1e-05 and a continuous overlapping region of 0.5. At most 150 homologous sequences for each protein were accepted. Homologous sequences were then aligned using three different programs: MUSCLE v3.7 [72], MAFFT v6.712b [73], and DIALIGN-TX [74]. Alignments were performed in forward and reverse direction (i.e. using the Head or Tail approach [75]), and the six resulting alignments were combined with M-Coffee [76]. This combined alignment was trimmed with trimAl v1.3 [77], (consistency-score cutoff 0.1667, gap-score cutoff 0.9). Trees were reconstructed using the best-fitting evolutionary model. The selection of the model best fitting each alignment was performed as follows: a Neighbour Joining (NJ) tree was reconstructed as implemented in BioNJ [78]; the likelihood of this topology was computed, allowing branch-length optimization, using seven different models (JTT, LG, WAG, Blosum62, MtREV, VT and Dayhoff), as implemented in PhyML v3.0 [79]; the two models best fitting the data, as determined by the AIC criterion [80], were used to derive ML trees. Four rate categories were used and invariant positions were inferred from the data. Branch support was computed using an aLRT (approximate likelihood ratio test) based on a chi-square distribution. Resulting trees and alignments are stored in phylomeDB [26] (http://phylomedb.org), with the phylome IDs 150 (*P. digitatum*) and 179 (*P. chrysogenum*). Trees were scanned automatically using ETE v2 [81].

#### Orthology prediction

Orthologs between the two *P. digitatum* strains were established using a best bidirectional hit approach. Orthologs between *P. digitatum* and the other species were based on phylogenies included in the phylome. A species-overlap algorithm, as implemented in ETE v2 [81] was used to infer orthology and paralogy relationships. Briefly the algorithm decides whether a node in a tree is a speciation of a duplication node depending on the overlap of the species branching from the node [11]. Overlap between those species will indicate a duplication node. Otherwise a speciation node will be considered.

#### Detection of horizontal gene transfers

In order to detect genes putatively acquired from prokaryotes we used the procedure described in [28], using a database of 102 completely-sequenced fungi, 95 non-fungal eukaryotes, and 1395 eukaryotes as downloaded from KEGG as of June 2011 [66]. Blast results were filtered using the same parameters as in the phylome reconstruction. Only proteins present in less than 10 fungal species, no other non-fungal eukaryotes and that were over-represented in bacterial species were considered to have a putative prokaryotic origin. These families were further analyzed phylogenetically.

#### Species tree reconstruction

The species tree was build using a concatenation method. 592 widespread, single copy genes were selected. After concatenation, the alignment was trimmed using trimAl [77]. Columns with more than 50% of gaps were removed. A conservation score of 50% of the alignment was used. The final alignment had 371,529 positions. The tree was reconstructed using RAxML version:7.2.6 [82]. LG model [83] was selected and a 4-categories GAMMA distribution was used. Bootstrap was obtained by creating 100 random sequences using SeqBoot from the Phylip package [84]. A tree was then reconstructed for each sequence and the consensus tree was inferred using Phylip. Additionally, we reconstructed a species tree based on the super-tree reconstruction program DupTree [85], which took as input the 7,723 trees obtained during phylome reconstruction. Both trees resulted in the same final topology.

#### Percentage of identity

Pairs of orthologous proteins were established from the phylogeny-based orthology predictions. Between *P. digitatum* and the other fungal species only one-to-one orthologs were considered. A pair-wise alignment between the orthologous pair was reconstructed using MUSCLE v3.7 [72]. The percentage of identity was calculated using the sident option of trimAl [77]. As the two strains of *P. digitatum* are highly similar, proteins that had a length difference superior to the average difference of all protein lengths were not considered.

#### Gene order conservation

The degree of gene order conservation between two species was calculated as the pairs of orthologous genes that were found consecutively in both genomes and were found in the same direction. Up to five genes without orthologs were allowed between a pair of genes.

#### Detection of single nucleotide polymorphisms (SNPs) and assessment of evolutionary rates

SNPs were detected by aligning the short reads produced during the sequencing of *P. digitatum* PHI26 to the genome of *P. digitatum* Pd1. Only reads mapping unambiguosly were considered. SAMtools [86] was then used to call variants, considering that the organism is haploid. Only SNPs in regions covered at least by 10 reads and with an agreement in the base call of >90% were considered.

Ratios of non-synonymous over synonymous substitutions (dN/dS) were calculated as follows. Alignments of the relevant one-to-one orthologs (*P. digitatum* vs *P. chrysogenum* for the nitrogen cluster and *P. digitatum* vs *A. clavatus* for patulin) were computed on the protein sequences using MUSCLE v3.7 [72]. The amino acid alignment was then back translated to nucleotides using the backtrans option implemented in trimAl v1.3 [77]. dN/dS was then calculated as the number of codons that codified for different amino acids in the two species versus the number of codons that contained synonymous mutations. As a control, the same analysis was performed against a set of proteins involved in the glycolysis pathway and for all one-to-one orthologs detected between these species.

### Experimental assessment of a possible role in fungicide resistance of selected genes

To investigate the possible role in the differential susceptibility to fungicides of the two contiguous genes that were only present in the Pd1 strain, we transformed this strain via *Agrobacterium tumefaciens*-mediated transformation with plasmid pRFD519. This plasmid derives from plasmid pRFHU2 [87] and contains the flanking regions of the two genes surrounding the hygromycin resistant cassette in the T-DNA region of the plasmid. Primers PD519O1 (5^′^- GGTCTTAAUGGGGAGAGCATAGGTGGAAT-3^′^) and PD519O2 (5^′^-GGCATTAAUCTTGGATGGAGAGGGAACAA-3^′^) were used to amplify a fragment of 1.76 kb region upstream of the genes and primers PD519A3 (5^′^- GGACTTAAUCGTGACATAAGGGCCAAACT-3^′^) and PD519A4 (5^′^- GGGTTTAAUCCCCTTTGGTTACCCTCATT-3^′^) were used to amplify a 1.85 kb DNA fragment downstream of the genes. The two flanking fragments were introduced into pRFHU2 following the USER protocol described by [87] and the resulting plasmid was introduced into *E. coli* DH5α chemical competent cells. Proper fusions were checked by DNA sequencing and then the plasmid was transferred to *A. tumefaciens* AGL1 electrocompetent cells. *Agrobacterium*-mediated transformation of *P. digitatum* Pd1 was conducted basically as described in [88] with minor modifications. Briefly, equal volumes of a conidial suspension adjusted at 10^5^ con/mL was mixed with *A. tumefaciens* AGL1 cells that had been grown in liquid induction medium (IM) supplemented with 200 μM acetosyringone until they reached an A_600_ of ~0.75. Aliquots of the mixture were spread onto 0.45 μm nitrocellulose membranes (Abet) that were layered on solid IM plates. After three days of incubation at 24°C the membranes were transferred to Petri plates containing PDA medium supplemented with 100 μg/mL hygromycin B (Invitrogen), for selection of fungal transformants, and 200 μg/mL cefotaxim (Calbiochem), for killing the bacterial cells. Putative transformants appeared after four days of incubation at 24°C. They were transferred to fresh plates containing PDA supplemented with hygromycin B and monosporic isolates were obtained. Conidia from monosporic isolates were inoculated in PDB medium containing 100 μg/mL hygromicin B and incubated at 24°C for two days with shaking. DNA was extracted from fungal mycelia following the procedure described in [89] and resuspended in 100 μL of TE. Transformants were analyzed by PCR with primers PD519F (5^′^-GCTTTCCCGCTTTAGTGTGG-3^′^) and PD519R (5^′^-ACAGCCGCAGCCTGTTATCT-3^′^). In the wild type strain these two primers amplify a fragment of 2.3 kb, whereas in gene knockout transformants this fragment is replaced by a 3.0 kb band. Ectopic transformants contain both bands.

Fungicide resistance assays were conducted in 96-well microtiter plates as described in [8]. Wells contained 10^5^ con/mL in PDB medium supplemented with fungicide. The fungicides azoxystrobin (Quadris; Syngenta Crop Protection), imazalil (Textar I; Tecnidex), myclobutanil (Thiocur 12; Rohmand Haas Italia SRL), prochloraz (Ascurit; Tecnidex) and trifloxystrobin (Flint; Bayer CropScience) were assayed at final concentrations of active ingredient of 0, 0.16, 0.31, 0.63, 1.25, 2.5, 5.0, and 10.0 μg/mL. Thiabendazole (Textar 60T; Tecnidex) was assayed at concentrations of 0, 0.63, 1.25, 2.5, 5.0, 10.0, 20.0 and 40.0 μg/mL. There were three replicate wells for each fungicide concentration. Plates were incubated at 24°C and growth, measured as A_600_, was followed for up to seven days. Minimum inhibitory concentration (MIC) values were considered as those in which no fungal growth was observed by seven days of incubation.

## Competing interests

The authors declare that they have no competing interests.

## Authors’ contributions

ARB, BF, EH, and LGC performed experiments. MMH and ARB conducted bioinformatic analyses. JFM, TG and LGC conceived the study. TG and LGC coordinated the work. TG, LGC, MMH, ARB and JFM analyzed the data and drafted the manuscript. All authors read and approved the final manuscript.

## Supplementary Material

Additional file 1 Supplementary materials. Click here for file
