# Supplementary material for: Genome sequence of the necrotrophic fungus Penicillium digitatum, the main postharvest pathogen of citrus
Source: BMC Genomics. 2012 Nov 21;13:646. doi: 10.1186/1471-2164-13-646 (PMC3532085; doi:10.1186/1471-2164-13-646)
Supplement: Additional file 1 — Supplementary materials. [file 1471-2164-13-646-S1.pdf]

Supplementary material for:

Genome sequence of the necrotrophic fungus *Penicillium digitatum*, the main postharvest pathogen of citrus.

Marina Marcet-Houben[1†], Ana-Rosa Ballester[2†], Beatriz de la Fuente[2], Eleonora Harries[2], Jose F. Marcos[2], Luis González-Candelas[2,*] and Toni Gabaldón[1,*] .

1.- Experimental assessment of the involvement of two Pd1 specific genes in fungicide resistance.

2.- Supplementary figure legends

3.- Supplementary table legends

4.- References

5.- Supplementary figures

6.- Supplementary tables

1.- Experimental assessment of the involvement of two Pd1 specific genes in fungicide resistance.

Isolate Pd1 contains two specific clustered genes, encoding proteins PDIP_31800 and PDIP_31810, that are absent in isolate PHI26. As the only known differences between these two strains are in their differential susceptibility to fungicides, we decided to generate a Pd1 mutant lacking both genes through gene disruption. Flanking regions, 5' upstream of PDIP_31800 and 3' downstream of PDIP_31810, were PCR-amplified and cloned into plasmid pRFHU2 (Supplementary figure 3). This plasmid was introduced into *A. tumefaciens* AGL1 cells, which were subsequently used to transform *P. digitatum* Pd1 conidia. Transformants were selected into PDA medium containing hygromycin B and monosporic isolates were obtained. Transformants were analyzed by PCR using two primers that flank both genes to detect deletion mutants. The amplicons generated in the parental strain and in a deletion mutant differ in size (Supplementary figure 4a). Out of sixty analyzed transformants we obtained three independent deletion mutants, named Δ30, Δ 59 and Δ60 (Supplementary figure 4b), which did not differ from the parental strain in growth rate and sporulation in PDA plates.

Susceptibility to six fungicides was analyzed in two of these deletion mutants (Δ30 and Δ60), an ectopic transformant (E12), the parental strain *P. digitatum* Pd1, and also *P. digitatum* PHI26 as a control. Both *P. digitatum* Pd1 and PHI26 showed the same susceptibility to azoxystrobin and trifloxystrobin. However, PHI26 was more susceptible to imazalil, myclobutanil, prochloraz and thiabendazole than Pd1 (Supplementary table 4 and Supplemental figure 5). For each fungicide Pd1 and the three mutants strains showed the same susceptibility. The highest MIC value (>40 µg/mL) was observed with thiabendazole, a benzimidazole fungicide. Resistance to the two strobilurin fungicides analyzed, azoxystrobin and trifloxistrobin was also high in the four strains, with a MIC value >10 µg/mL. Sensitivity of the strains is different to the two imidazole fungicides assayed, imazalil and prochloraz. All strains are more resistant to imazalil, MIC value of 5 µg/mL, than to prochloraz, which is the most active among the tested fungicides. Finally, myclobutanyl, a triazole fungicide, shows low activity against all the strains, with a MIC value of 10 µg/mL.

We further analyzed whether deletion mutants were affected in their pathogenicity towards citrus fruits. Statistical analysis of two independent experiments indicate that Δ30 and Δ60 deletion mutants showed the same virulence towards citrus fruits as the parental Pd1 strain and the ectopic E12 transformant. The results of both the experiments are shown in Supplementary figure 6.

2.- Supplementary figure legends

Supplementary figure 1.- Representation of the conserved MAT locus found in *Penicillium* and *Aspergillus* species. For each species a row of syntenic genes is shown, the colored square marked as MAT1 or MAT2 represents the mating locus, the remaining squares represent surrounding genes. U1 and U2 stand for conserved uncharacterized proteins. For *P. digitatum* the locus tags for each gene is added.

Supplementary figure 2.- Representation of the two inversions found when comparing the two newly sequenced strains of *P. digitatum*. Each square represents a gene and they are ordered as found in a given scaffold. Genes with the same coloring and number are orthologs between the two strains. The locus tag of each gene is included.

Supplementary figure 3.- Physical map of plasmid pRFD519. This plasmid is a derivative of plasmid pRFHU2 in which 5' and 3' flanking regions of *P. digitatum* Pd1 PDIP_31800 and PDIP_31810 genes were cloned at both sites of the hygromycin (hph) resistant marker. P*trpC*: *Aspergillus nidulans trpC* gene promoter. T*trpC*: *A. nidulans trpC* gene terminator. kanR: kanamycin resistant gene. TrfA: replication origin gene for *A. tumefaciens*. OriV: replication origin for *E. coli*. LB: T-DNA left border. RB: T-DNA right border.

Supplementary figure 4.- Analysis of *P digitatum* Pd1 transformants. a) Schematic representation of the locus containing the genes PDIP_31800 and PDIP_31810 in the wild type (Pd1) strain and in a deletion mutant. Homologous recombination leads to the replacement of the two genes by the hygromycin resistant cassette. Location of primers used for analysis of transformants as well as the excepted size of the amplicons are also indicated. b) Agarose gel electrophoresis of DNA fragments obtained by PCR amplification with primers PD519F and PD519R and DNA derived from Pd1 transformants. "E" and "Δ" transformants correspond to ectopic and deletion transformants, respectively. No template control as well as PCR amplification of the parental Pd1 strain are also included.

Supplementary figure 5.- Dose response curves of *P. digitatum* PHI26, *P. digitatum* Pd1, Pd1 deletion mutants (Δ30 and Δ60) and an Pd1 ectopic transformant (E12) to six fungicides. Fungal conidia ($10e^5$ conidia/mL) were cultured in PDB in the presence of increasing concentrations of the fungicides. Samples were prepared in triplicate. Plates were incubated at 24 ºC and growth, measured as A_{600}, was followed for up to seven days. Data show the mean values ± SD at each fungicide concentration. Dose

is given as µg of active ingredient per mL.

Supplementary figure 6.- Pathogenicity assays of *P digitatum* Pd1 parental strain, Δ30 and Δ60 deletion transformants, and E12 ectopic transformant towards citrus fruits. Navel (Experiment 1) or Lanelate (Experiment 2) oranges were wounded and inoculated with 10 µL of spore suspensions of the different fungi. Fruits were incubated at 20 ºC and 90 % relative humidity for five days. There were three replicates of five fruits. Bars show the mean values of the percentage of infected wounds and their standard deviation at three, four and five days post-inoculation (dpi). Bars labeled with the same letter do not differ at the 95% confidence level (least significant difference test). Statistical analysis was conducted independently for four and five dpi.

Supplementary figure 7.- Pie charts comparing the COG functional classes in *P. digitatum* and *P. chrysogenum*.

Supplementary figure 8.- Genomic organization of the patulin gene cluster in *P. digitatum* PHI26 and *A. clavatus*. Homologous genes are indicated with arrows of the same color. Genes homologous to black colored *A. clavatus* pat genes are found dispersed in other scaffolds in the genome of *P. digitatum* PHI26. Asterisk in patI* indicates that this gene is a putative truncated homolog of the *A. clavatus* PatI gene. Patulin cluster of *A. clavatus* adapted from Artigot et al [1].

Supplementary figure 9.- Flow chart showing the three steps used during the gene annotation process. For each step the number of additionally annotated genes in *P. digitatum* PHI26 is shown.

3.- Supplementary table legends

Supplementary table 1.- List of repetitive regions detected in *P. digitatum* and *P. chrysogenum*.

Supplementary table 2.- List of strain-specific genes in each *P. digitatum* strain. Strain specific genes are only considered as such when no reads of the opposite strain map to it.

Supplementary table 3.- List of genes that contain more than one non-synonymous mutation when comparing the two *P. digitatum* strains. First and second column represent the codes of the orthologous

gene (NO BRH means no best reciprocal hit was found in the PHI26 strain). Third and fourth column show the synonymous and non-synonymous mutations respectively and the last column shows the annotation of the genes.

Supplementary table 4.- Minimum inhibitory concentration (MIC) values of different fungicides against *P digitatum* PHI26, *P digitatum* Pd1, Pd1 deletion transformants Δ30 and Δ60 and Pd1 ectopic transformant E12. Values are expressed in µg/mL.

Supplementary table 5.- List of proteomes used during phylome reconstruction and at different stages of this analysis. The first column shows the NCBI taxonomy ID, second column contains the species name, third column is the source of the proteome and the last column shows the number of proteins in each species.

Supplementary table 6.- List of over-represented Pfam domains found in the scaffolds of *P. chrysogenum* that do not have homologs in *P. digitatum* PHI26.

Supplementary table 7.- List of *P. digitatum* proteins that only contain homologs in the other plant-pathogenic species included in the phylome. Columns represent gene name in *P. digitatum* PHI26, its ortholog in *P. digitatum* Pd1 and the annotation.

Supplementary table 8.- List of candidate proteins that may have resulted from a horizontal gene transfer from prokaryotes. Columns represent gene name in *P. digitatum* PHI26, its ortholog in *P. digitatum* Pd1 and the annotation.

Supplementary table 9.- List of secondary metabolism backbone genes as predicted by SMURF [2]

Supplementary table 10.- dN / dS analysis of patulin orthologs between *Aspergillus clavatus* and *P. digitatum* PHI26.

Supplementary table 11.- List of putative small cysteine- rich proteins (CRPs) predicted in *P. digitatum* PHI26. Columns represent assigned name, scaffold, position and protein sequence.

Supplementary table 12.- List of proteins that either contain a LysM domain or have been detected as

orthologs to a LysM protein in another species used in the phylome. Columns represent gene name in *P. digitatum* PHI26, its ortholog in *P. digitatum* Pd1 and the annotation.

Supplementary table 13.- Summary of CAZy enzymes found in four fungal species including the two *P. digitatum* strains.

Supplementary table 14.- List of secreted proteins predicted in the two *P. digitatum* strains. Columns represent gene name in *P. digitatum* PHI26, its ortholog in *P. digitatum* Pd1 and the annotation.

4.- References:

1. Artigot MP, Loiseau N, Laffitte J, Mas-Reguieg L, Tadrist S, Oswald IP, Puel O: **Molecular cloning and functional characterization of two CYP619 cytochrome P450s involved in biosynthesis of patulin in Aspergillus clavatus.** *Microbiology* 2009, **155(Pt 5)**:1738-1747.

2. Khaldi N, Seifuddin FT, Turner G, Haft D, Nierman WC, Wolfe KH, Fedorova ND: **SMURF: Genomic mapping of fungal secondary metabolite clusters.** *Fungal genetics and biology : FG & B* 2010, **47**:736-41.

5.- Supplementary figures

Supplementary figure 1

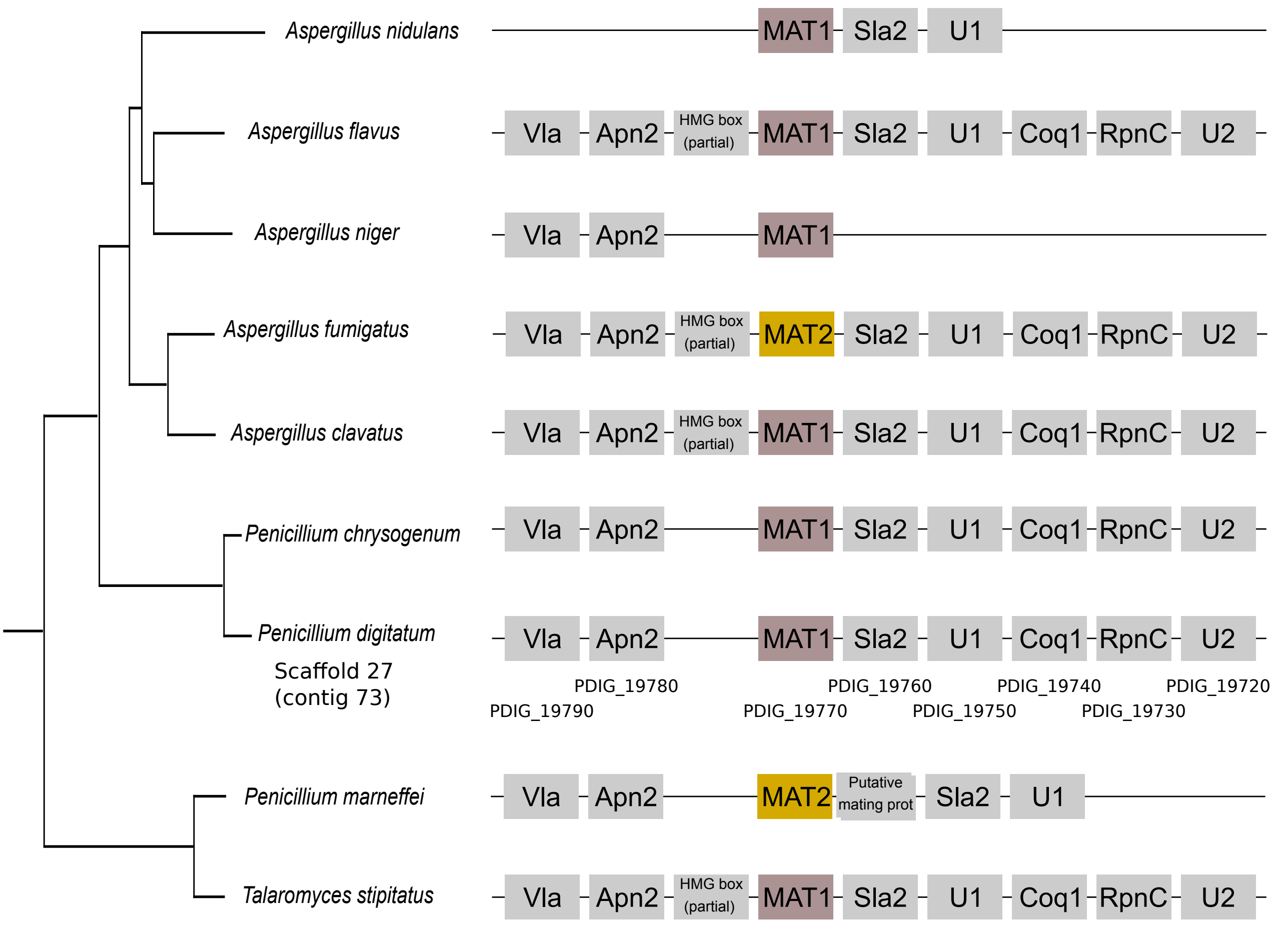

Supplementary figure 2

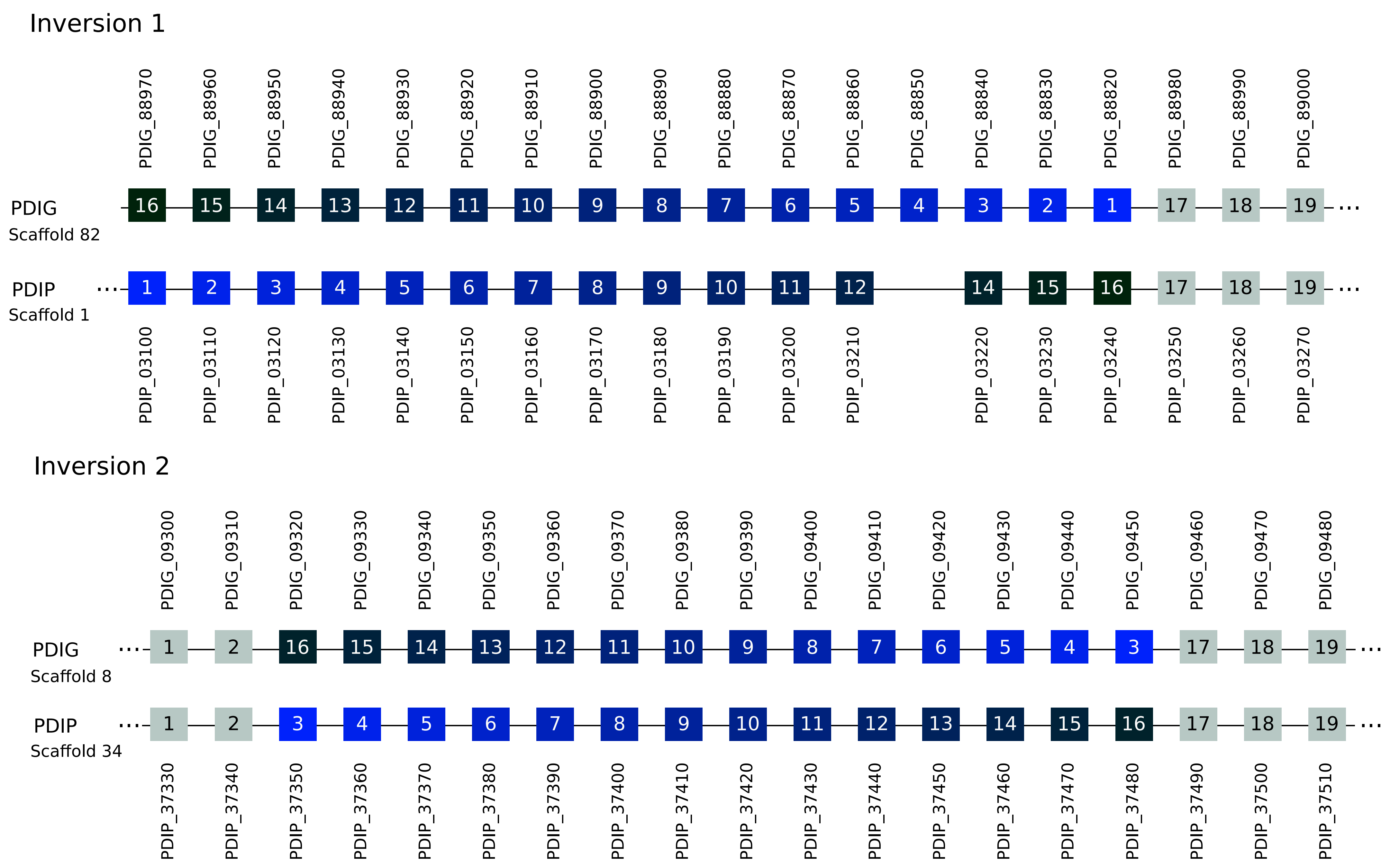

Supplementary figure 3

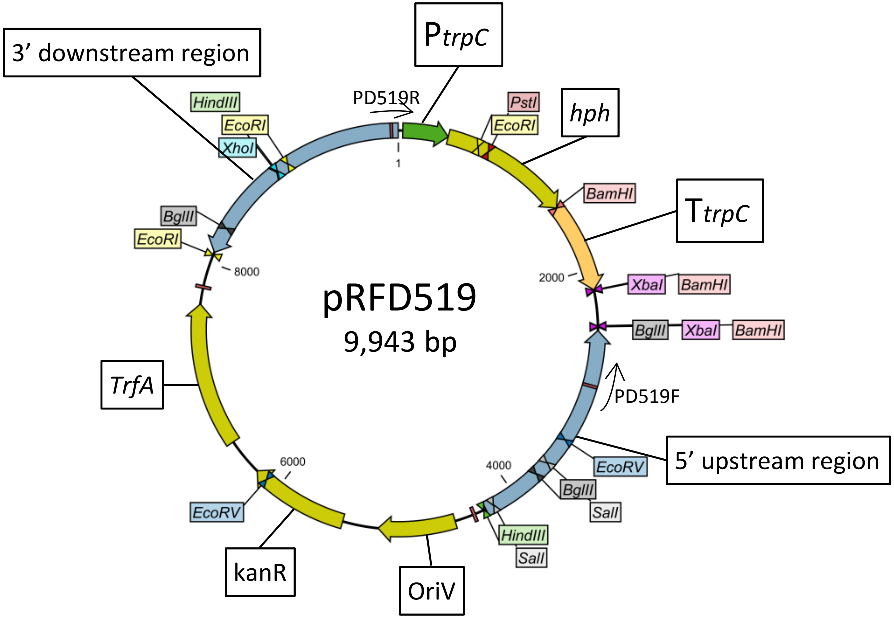

Supplementary figure 4

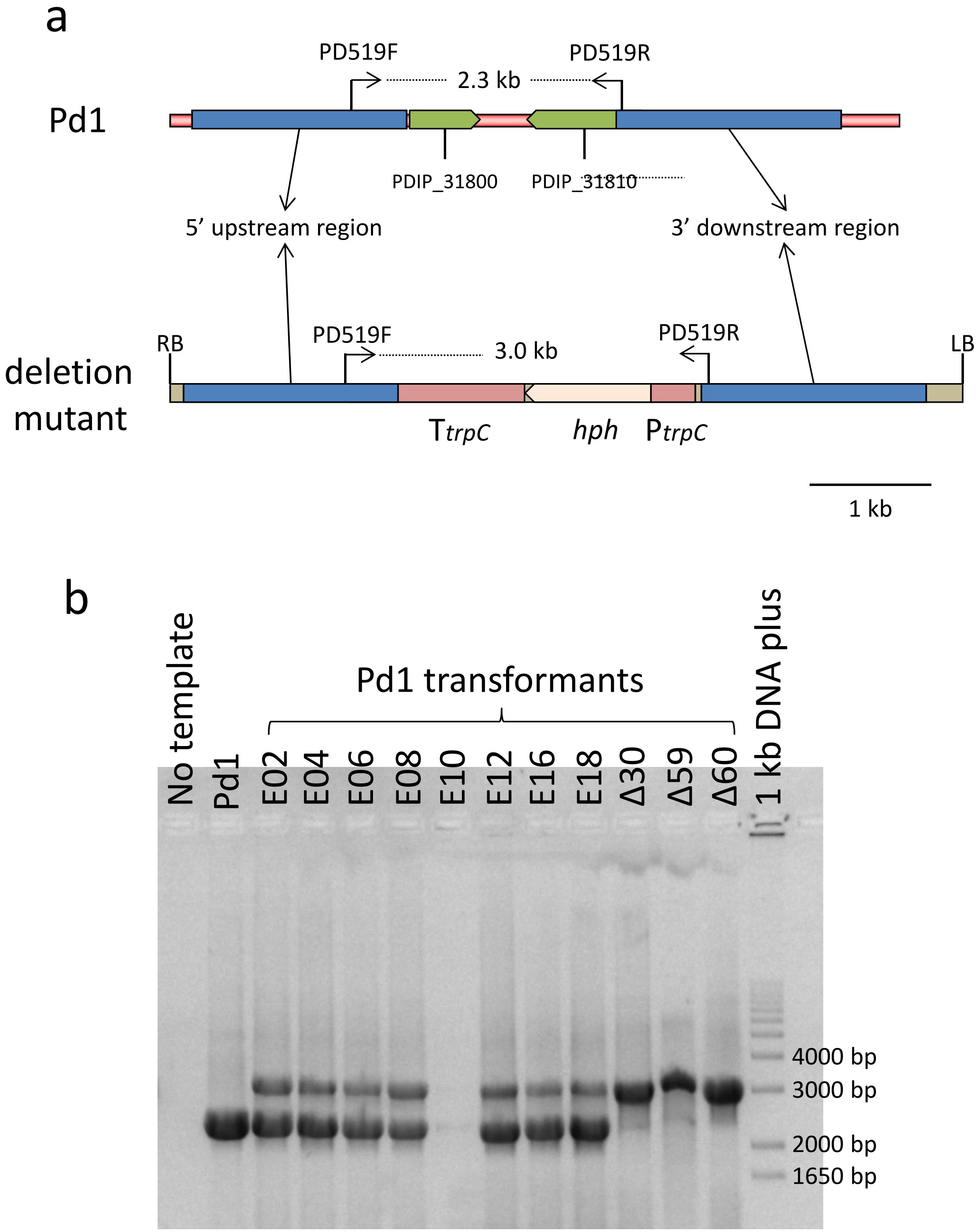

Supplementary figure 5

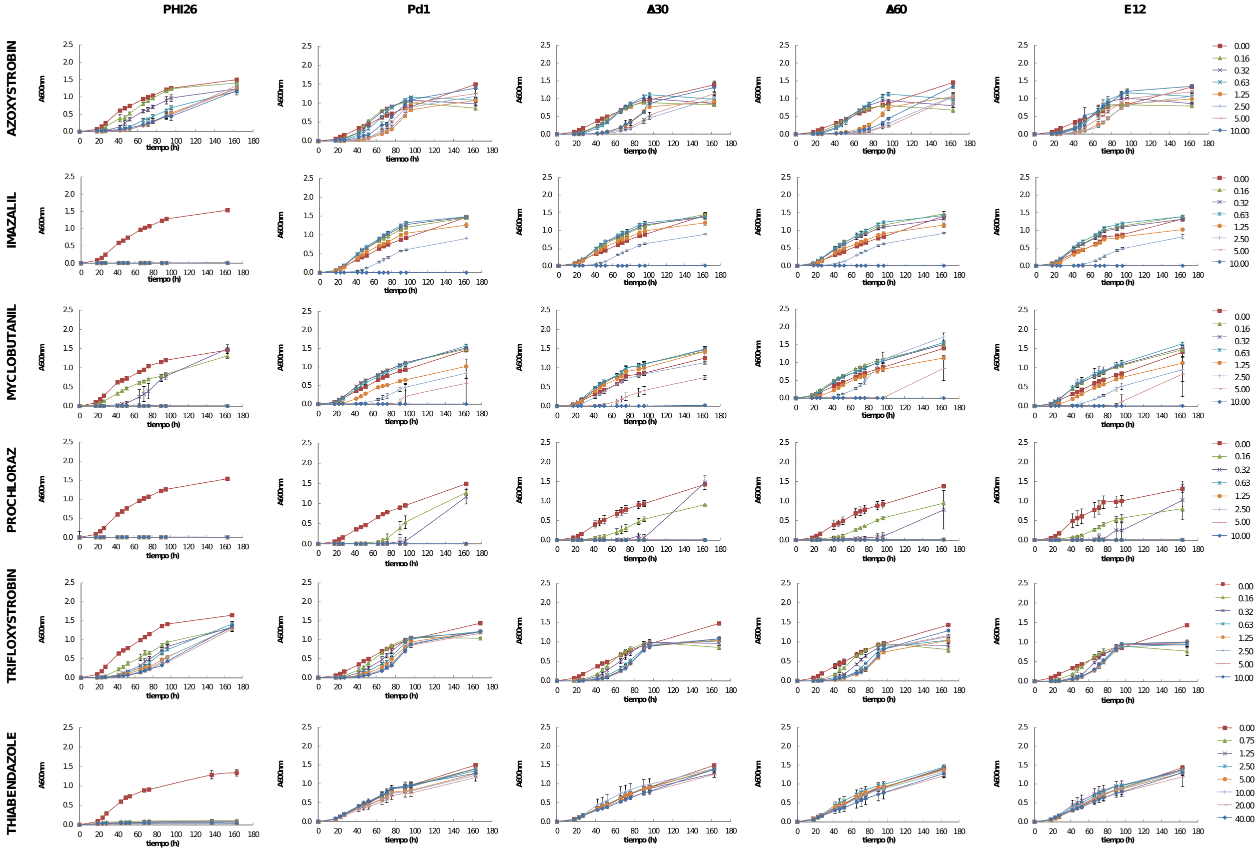

Supplementary figure 6

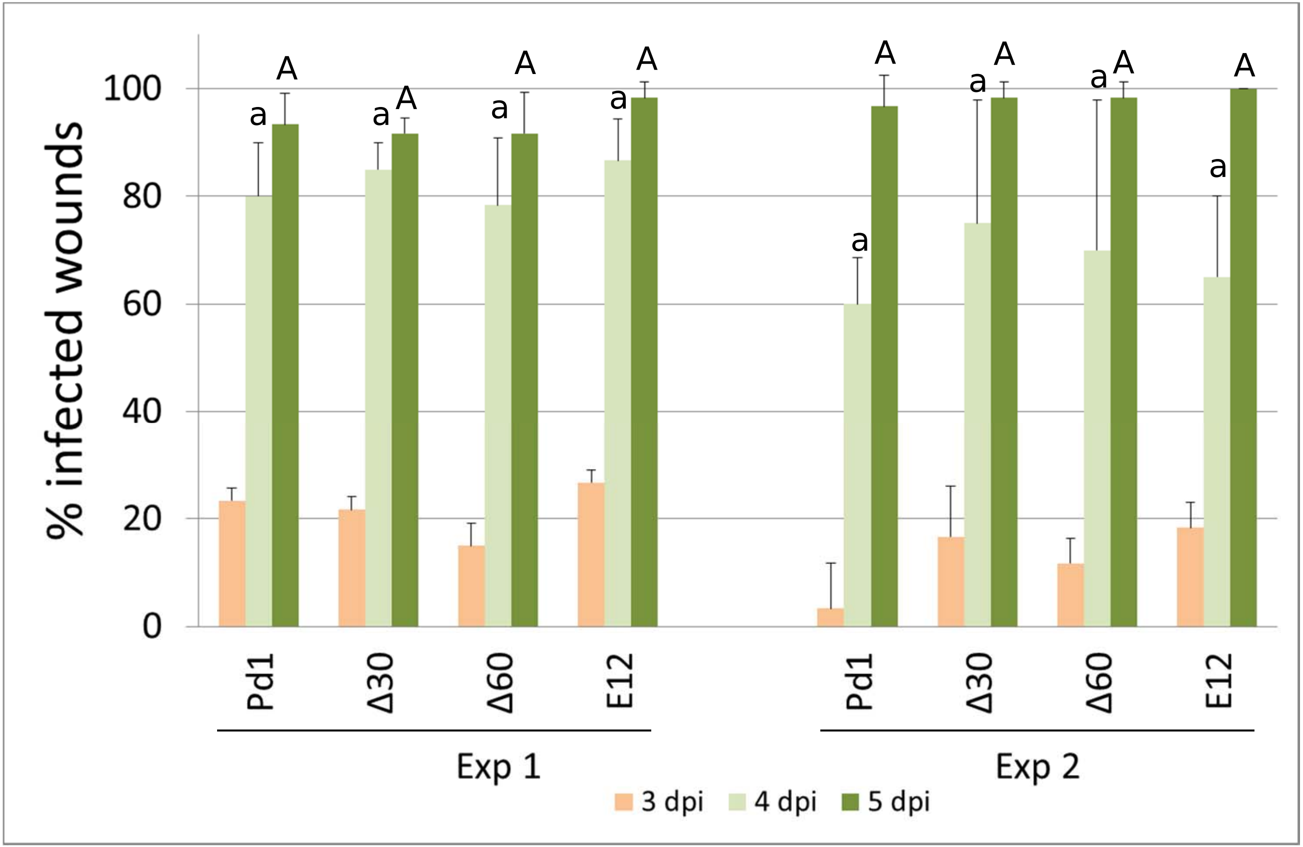

Supplementary figure 7

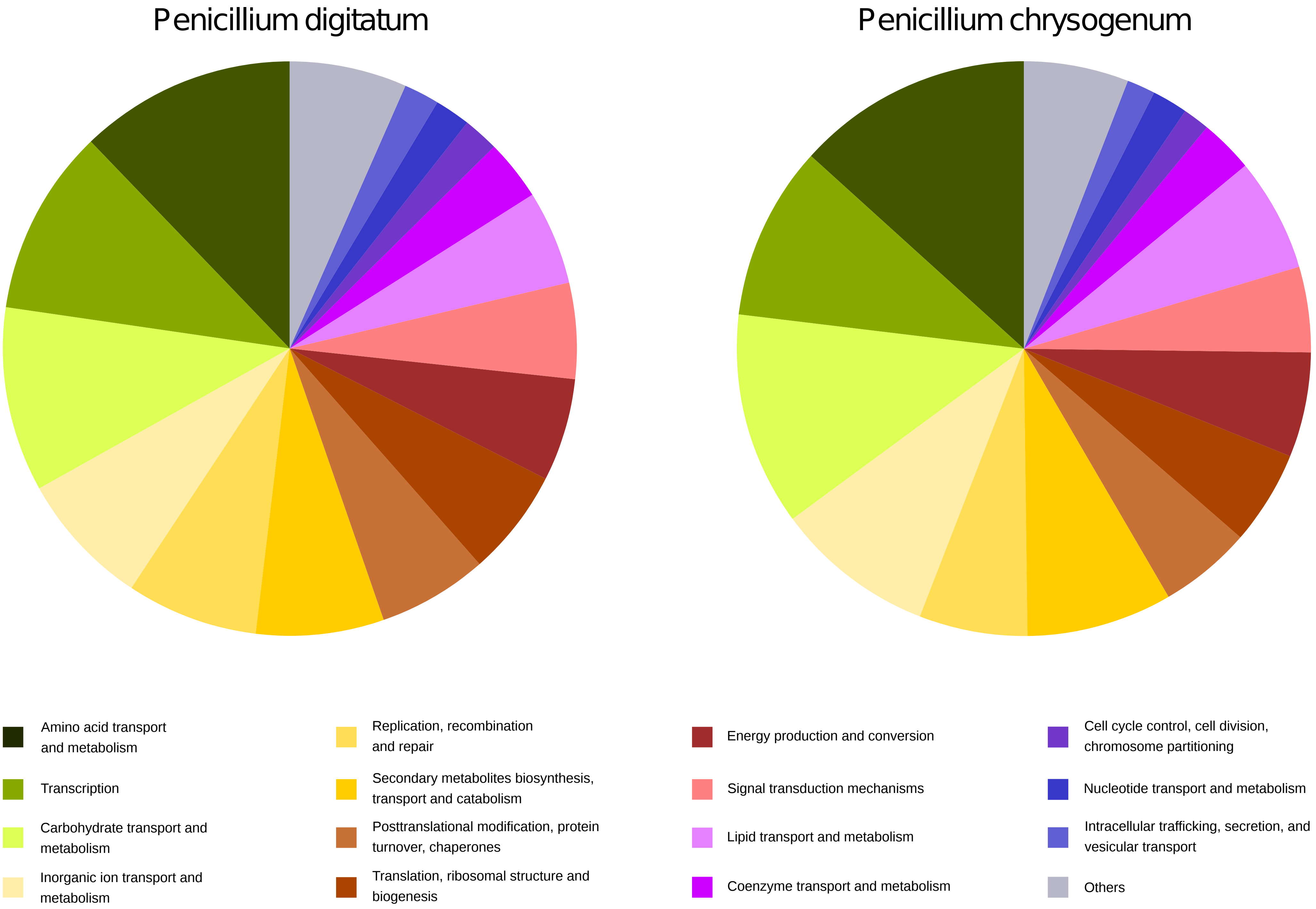

Supplementary figure 8

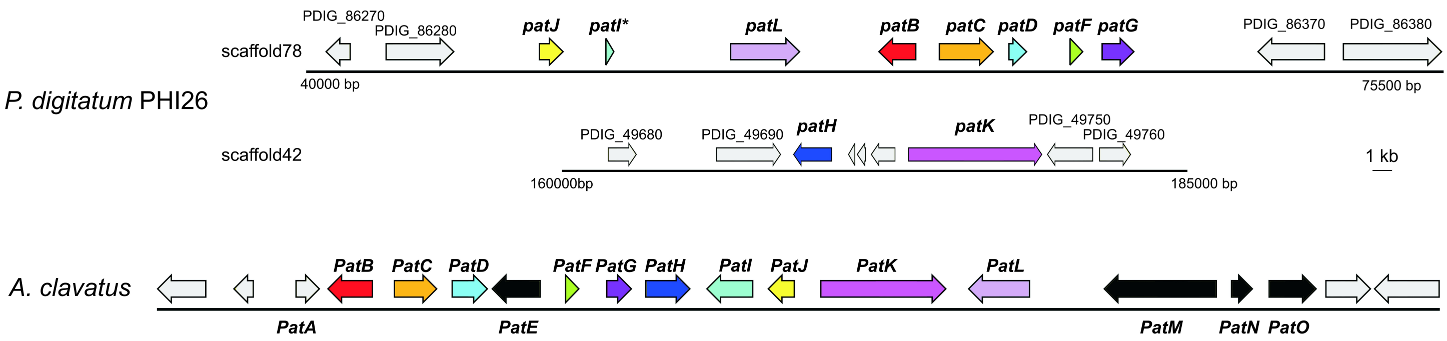

Supplementary figure 9

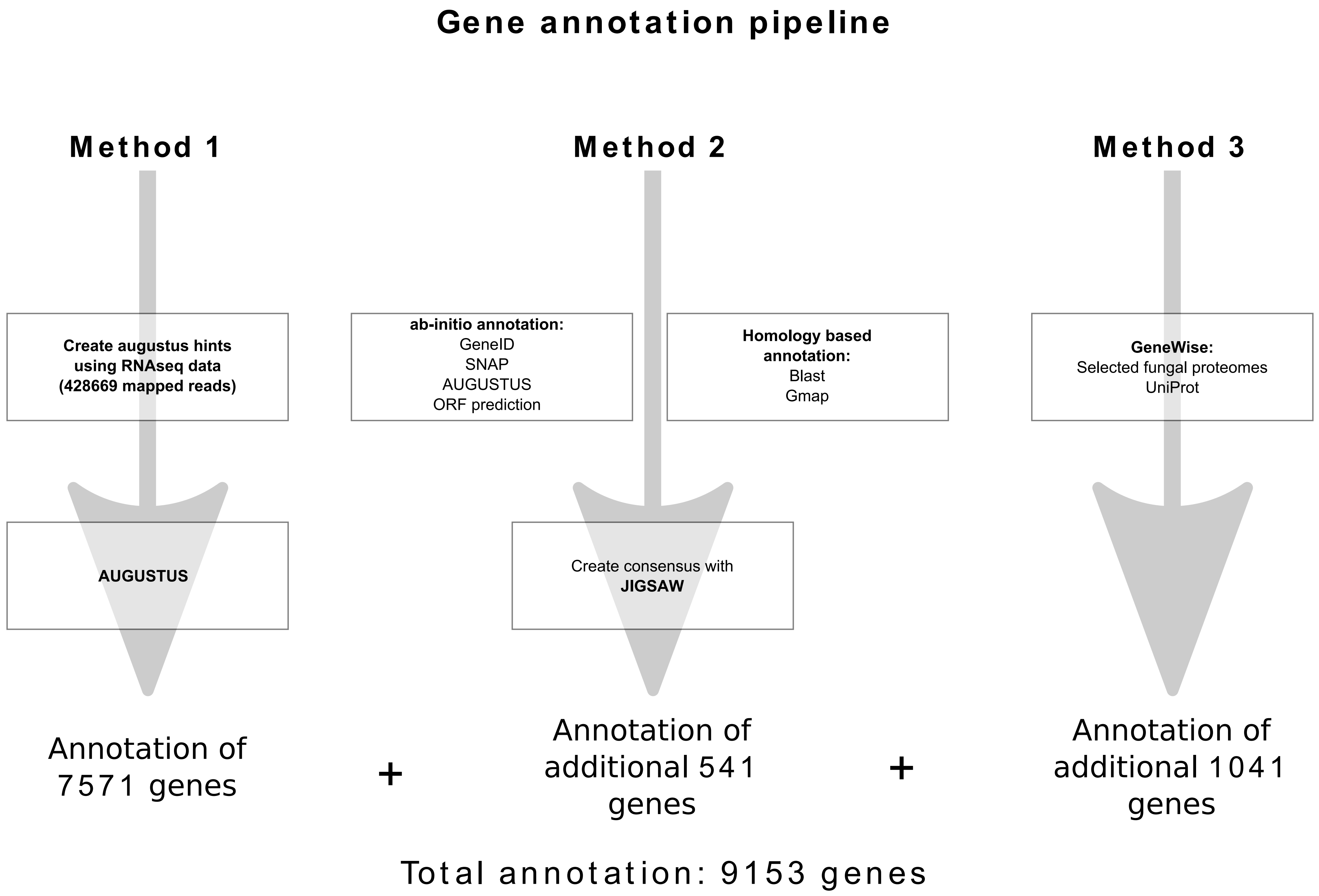

6.- Supplementary Tables

			Supplementary table 1: List of repetitive elements					
			***P. digitatum* PHI26**			***P. chrysogenum***		
			Number of elements	bp included	Percentage in genome	Number of elements	bp included	Percentage in genome
Retroelements	SINEs		6	971 bp	0.00 %	11	1385 bp	0.00 %
	Penelope		4	444 bp	0.00 %	0	0 bp	0.00 %
	LINEs	LINEs	106	42303 bp	0.16 %	92	97053 bp	0.08 %
		CRE/SLACS	0	0 bp	0.00 %	0	0 bp	0.00 %
		L2/CR1/Rex	0	0 bp	0.00 %	0	0 bp	0.00 %
		R1/LOA/Jockey	0	0 bp	0.00 %	0	0 bp	0.00 %
		R2/R4/NeSL	0	0 bp	0.00 %	0	0 bp	0.00 %
		RTE/Bov-B	0	0 bp	0.00 %	0	0 bp	0.00 %
		L1/CIN4	0	0 bp	0.00 %	0	0 bp	0.00 %
	LTR elements	BEL/Pao	0	0 bp	0.00 %	0	0 bp	0.00 %
		Ty1/Copia	38	14521 bp	0.06 %	84	36114 bp	0.11 %
		Gypsy/DIRS1	103	51283 bp	0.20 %	88	53012 bp	0.16 %
		TOTAL	253	109078 bp	0.42 %	172	89126 bp	0.28 %
Dna transposons	hobo-Activator		61	18518 bp	0.07 %	25	9044 bp	0.03 %
	Tc1-IS630-Pogo		245	102945 bp	0.40 %	168	103781 bp	0.32 %
	En-Spm		0	0 bp	0.00 %	0	0 bp	0.00 %
	MuDR-IS905		1	57 bp	0.00 %	1	113 bp	0.00 %
	PiggyBac		0	0 bp	0.00 %	0	0 bp	0.00 %
	Tourist/Harbinger		0	0 bp	0.00 %	0	0 bp	0.00 %
	Other (Mirage, P-element,Transib)		0	0 bp	0.00 %	0	0 bp	0.00 %
	TOTAL		313	126891 bp	0.49 %	199	118182 bp	0.37 %
Unclassified:			2	242 bp	0.00 %	3	309 bp	0.00 %
Small RNA:			56	6278 bp	0.02 %	68	10676 bp	0.03 %
Satellites:			2	178 bp	0.00 %	1	114 bp	0.00 %
Simple repeats:			1092	74290 bp	0.29 %	1270	66536 bp	0.21 %
Low complexity:			877	44560 bp	0.17 %	1483	81628 bp	0.25 %

Supplementary table 2: Comparison of the protein content between the two Penicillium digitatum strains			
**Proteins missing in *P. digitatum Pd1***		**Proteins missing in *P. digitatum PHI26***	
PDIG_03320	Putative protein with kinase activity	PDIP_31800	Phosphotransferase enzyme family protein
PDIG_03340	Putative protein	PDIP_31810	Uncharacterized protein
PDIG_03350	Putative protein		
PDIG_03360	Putative protein contains DUF2841 domain		
PDIG_76440	Metalloreductase transmembrane component, putative		
PDIG_17070	Putative protein		
PDIG_81290	Putative protein, low-complexity protein		
PDIG_81500	Putative protein, low-complexity protein		

Supplementary table 3: List of genes containing more than one non-synonymous substitution				
***P. digitatum* Pd1**	***P. digitatum* PHI26**	**Synonym mutations**	**Non-synonymous mutations**	**Annotation**
PDIP_19890	PDIG_50650	0	2	Putative uncharacterized protein [Ortholog: *Aspergillus fumigatus* : Q4WJQ7]
PDIP_16880	PDIG_44900	0	2	MFS transporter, putative [Ortholog: *Talaromyces stipitatus* : B8M635]
PDIP_34330	PDIG_18910	21	13	Putative uncharacterized protein [Ortholog: *Emericella nidulans* : Q5AST2]
PDIP_87550	PDIG_79170	4	12	Putative uncharacterized protein [Ortholog: *Talaromyces stipitatus* : B8LX17]
PDIP_27600	NO BRH	0	3	Putative protein
PDIP_29400	PDIG_33240	2	2	Similarity to reverse transcriptase pol - Volvox carteri [Ortholog: *Aspergillus niger* : A2QJ59]
PDIP_30470	PDIG_14950	0	2	Elongation factor 1-beta [Ortholog: *Uncinocarpus reesii* : C4JTV2]
PDIP_27160	PDIG_78750	0	2	Equisetin synthetase, putative [Ortholog: *Aspergillus clavatus* : A1CPQ3]
PDIP_36000	PDIG_07960	0	2	Ubiquitin-protein ligase Ufd4, putative [Ortholog: *Aspergillus fumigatus* : Q4WMS5]
PDIP_36620	PDIG_08600	0	2	Putative uncharacterized protein [Ortholog: *Emericella nidulans* : Q5BC08]
PDIP_41980	NO BRH	6	6	Putative protein
PDIP_52580	PDIG_33020	5	2	Nuclear migration protein (ApsA), putative [Ortholog: *Aspergillus fumigatus* : Q4WU99]
PDIP_47650	PDIG_13650	0	2	MATH and UCH domain protein, putative [Ortholog: *Aspergillus fumigatus* : Q4WE48]
PDIP_52700	NO BRH	4	3	Putative protein
PDIP_61970	PDIG_27530	0	2	Serine/threonine protein kinase (Kcc4), putative [Ortholog: *Aspergillus fumigatus* : Q4WCS9]
PDIP_62320	PDIG_27880	0	2	Rad21/Rec8 N terminal domain protein [Ortholog: *Aspergillus fumigatus* : Q4WCZ0]
PDIP_80270	PDIG_70950	0	2	Integral membrane protein [Ortholog: *Aspergillus fumigatus* : Q4WZ73]
PDIP_81390	PDIG_72120	0	2	Similar to Pc21g02960 protein (Uncharacterized protein)
PDIP_82570	PDIG_11060	0	2	Topoisomerase family protein TRF4, putative [Ortholog: *Aspergillus fumigatus* : Q4WG84]
PDIP_71770	PDIG_62390	0	2	Predicted protein [Ortholog: *Uncinocarpus reesii* : C4JER5]
PDIP_06070	PDIG_41990	0	2	ABC multidrug transporter, putative [Ortholog: *Aspergillus fumigatus* : Q4WT65]
PDIP_33880	PDIG_87860	0	2	Proteasome activator subunit 4, putative [Ortholog: *Aspergillus fumigatus* : Q4WQ22]

NO BRH: No best reciprocal hit was foung in the PHI26 strain

Supplementary table 4: Minimum inhibitory concentration values (μg/mL)					
Fungicide name	**PHI26**	**Pd1**	**Δ30**	**Δ60**	**E12**
Azoxystrobin	>10	>10	>10	>10	>10
Imazalil	<0.16	5	5	5	5
Myclobutanil	0.63	10	10	10	10
Prochloraz	<0.16	0.63	0.63	0.63	0.63
Trifloxystrobin	>10	>10	>10	>10	>10
Thiabendazole	<0.63	>40	>40	>40	>40

Supplementary table 5: List of proteomes used in the phylome			
**Taxonomy ID**	**Species name**	**Source**	**Proteome size**
5037	*Ajellomyces capsulata*	Broad Institute	9271
602072	*Aspergillus carbonarius*	JGI	11184
5057	*Aspergillus clavatus*	UniProt reference genomes	9120
5059	*Aspergillus flavus*	UniProt reference genomes	13486
5085	*Aspergillus fumigatus*	UniProt reference genomes	9630
5061	*Aspergillus niger*	UniProt reference genomes	13905
40559	*Botrytis fuckeliana*	Broad institute	16400
5501	*Coccidioides immitis*	Broad institute	9750
5016	*Cochliobolus heterostrophus*	JGI	9623
5116	*Cryphonectria parasitica*	JGI	11145
162425	*Emericella nidulans*	UniProt reference genomes	9417
5507	*Fusarium oxysporum*	Broad institute	16646
36779	*Gaeumannomyces graminis*	Broad institute	14456
5518	*Gibberella zeae*	BroadInstitute	13318
645133	*Glomerella graminicola*	Broad institute	12005
148305	*Magnaporthe grisea*	UniProt reference genomes	12686
242507	*Magnaporthe oryzae 70-15*	Broad institute	12681
54734	*Mycosphaerella graminicola*	JGI	10938
140110	*Nectria haematococca*	JGI	15678
5141	*Neurospora crassa*	UniProt reference genomes	9826
5076	*Penicillium chrysogenum*	Hyphal tip	12768
36651	*Penicillium digitatum PHI26*	Sequenced CRG	9158
441960	*Penicillium marneffei ATCC 18224*	Hyphal tip	10001
13684	*Phaeosphaeria nodorum*	Broad institute	12374
426418	*Pyrenophora tritici-repentis*	Broad Institute	12062
5180	*Sclerotinia sclerotiorum*	Broad institute	14457
441959	*Talaromyces stipitatus*	NCBI	12996
336963	*Uncinocarpus reesii*	Broad Institute	7771
526221	*Verticillium albo-atrum*	Broad Institute	10218

Supplementary table 6: Pfam domains found in missing scaffolds of P. chrysogenum				
**Pfam domain**	**Pfam domain description**	**Times found in a missing scaffold**	**Times found in whole genome**	**%**
PF05699.8	hAT family dimerisation domain	10	13	0.77
PF02892.9	BED zinc finger	2	3	0.67
PF00665.20	Integrase core domain	5	8	0.63
PF07727.8	Reverse transcriptase	5	9	0.56
PF04516.9	CP2 transcription factor	2	4	0.50
PF11034.2	Protein of unknown function (DUF2823)	2	4	0.50
PF11402.2	Antifungal protein	1	2	0.50
PF11327.2	Protein of unknown function (DUF3129)	1	2	0.50
PF11702.2	Protein of unknown function (DUF3295)	1	2	0.50
PF01329.13	Pterin 4 alpha carbinolamine dehydratase	1	2	0.50
PF10330.3	Putative Sin3 binding protein	1	2	0.50

Supplementary table 7: Proteins with homologs only in plant pathogens		
***P. digitatum* PHI26**	***P. digitatum* Pd1**	**Putative function**
PDIG_00220	NO BRH	Putative protein
PDIG_00560	PDIP_02820	Putative protein
PDIG_00610	PDIP_02870	Putative protein
PDIG_01130	PDIP_12450	*Similarity: the ORF shows also weak similarity to proteins containing PTS domain [Blast hit trembl: Aspergillus niger: A2QI06]*
PDIG_02400	PDIP_13690	*DNA mismatch repair protein pms1 [Ortholog: Verticillium albo-atrum : C9SJ63]*
PDIG_02720	PDIP_13980	*Putative uncharacterized protein [Blast hit trembl: Metarhizium robertsii: E9F3N7]*
PDIG_03050	NO BRH	*Predicted protein [Ortholog: Pyrenophora tritici-repentis : B2WJT8]*
PDIG_04460	NO BRH	Putative protein
PDIG_04520	PDIP_09180	Putative protein
PDIG_04950	PDIP_09620	Putative succinate-semialdehyde dehydrogenase C1002.12c [NADP+]
PDIG_06490	PDIP_11130	Putative protein
PDIG_07070	PDIP_11720	Putative protein
PDIG_07190	PDIP_11840	GDP-mannose-dependent alpha-mannosyltransferase
PDIG_07290	PDIP_11940	Putative protein
PDIG_07550	PDIP_81670	Putative protein
PDIG_07840	PDIP_35880	*Putative uncharacterized protein [Ortholog: Phaeosphaeria nodorum : Q0U7C4]*
PDIG_08260	PDIP_36280	Putative protein
PDIG_08680	PDIP_36700	Putative protein
PDIG_09760	NO BRH	Putative protein
PDIG_11540	PDIP_37790	Putative protein
PDIG_13490	PDIP_40620	Uncharacterized transporter
PDIG_13920	PDIP_39570	Putative protein
PDIG_15070	PDIP_30600	Putative uncharacterized protein
PDIG_15360	PDIP_30890	Putative protein
PDIG_16000	NO BRH	Putative protein
PDIG_16660	PDIP_88170	Putative protein
PDIG_17870	PDIP_55740	Putative protein
PDIG_17980	NO BRH	Protein kinase
PDIG_18580	PDIP_56410	*Putative uncharacterized protein [Ortholog: Magnaporthe grisea : A4R093]*
PDIG_18920	NO BRH	Putative protein
PDIG_20980	PDIP_32170	Putative protein
PDIG_20990	PDIP_23270	Putative protein
PDIG_21140	PDIP_23420	*Catechol O-methyltransferase [Ortholog: Pyrenophora tritici-repentis : B2WAW0]*
PDIG_21620	PDIP_23900	Putative protein
PDIG_25110	PDIP_59600	Putative protein
PDIG_26330	NO BRH	Putative protein
PDIG_26590	PDIP_61040	*Mannan endo-1,4-beta-mannosidase F [Blast hit: Aspergillus clavatus: A1C8U0]*
PDIG_26730	PDIP_61180	*3-hydroxyacyl-CoA dehydrogenase, NAD binding domain, putative [Blast hit trembl: Metarhizium acridum: E9E516]*
PDIG_29480	PDIP_63880	*Putative uncharacterized protein [Blast hit trembl: Neosartorya fischeri: A1DGZ8]*
PDIG_31970	PDIP_51550	Putative protein

PDIG_32280	PDIP_51870	Uncharacterized membrane protein
PDIG_32420	PDIP_52010	Putative protein
PDIG_34180	NO BRH	Putative protein
PDIG_34460	PDIP_54020	Putative protein
PDIG_34890	NO BRH	Putative protein
PDIG_35620	PDIP_05850	Putative protein
PDIG_36250	PDIP_05230	Putative protein
PDIG_36330	PDIP_05160	*Putative uncharacterized protein [Ortholog: Verticillium albo-atrum : C9SZ05]*
PDIG_37970	PDIP_84550	Putative protein
PDIG_38400	PDIP_85030	Putative protein
PDIG_38980	PDIP_46900	Putative protein
PDIG_39000	PDIP_46920	*Endonuclease/exonuclease/phosphatase family [Ortholog: Verticillium albo-atrum : C9SNJ1]*
PDIG_39010	NO BRH	Putative protein
PDIG_39400	PDIP_24950	Putative protein
PDIG_41340	NO BRH	Putative protein
PDIG_41360	PDIP_86260	Putative protein
PDIG_41450	PDIP_86360	*Putative uncharacterized protein [Blast hit trembl: Metarhizium robertsii: E9EUZ3]*
PDIG_42340	PDIP_40920	Putative agmatinase 1
PDIG_43120	PDIP_41690	Putative protein
PDIG_43370	PDIP_34600	Putative protein
PDIG_43910	PDIP_35140	Putative protein
PDIG_45380	PDIP_17320	Putative protein
PDIG_45730	PDIP_17670	*Putative uncharacterized protein [Ortholog: Pyrenophora tritici-repentis : B2W554]*
PDIG_46440	PDIP_18360	Putative protein
PDIG_46930	PDIP_18850	Putative protein
PDIG_47080	PDIP_19010	Putative protein
PDIG_47560	PDIP_56940	Putative protein
PDIG_47870	PDIP_57250	*Putative uncharacterized protein [Ortholog: Verticillium albo-atrum : C9SM17]*
PDIG_47980	PDIP_57360	*ABC-type Fe3+ transport system [Ortholog: Pyrenophora tritici-repentis : B2W446]*
PDIG_48830	PDIP_58210	*Serine/threonine-protein kinase tel1 [Blast hit: Neosartorya fumigata: Q4WVM7]*
PDIG_49940	PDIP_85680	Putative protein
PDIG_53670	PDIP_48890	Structure-specific endonuclease subunit slx1
PDIG_56960	PDIP_66510	Putative protein
PDIG_57010	PDIP_66560	Putative protein
PDIG_60480	PDIP_69890	40S ribosomal protein S26E
PDIG_61260	PDIP_70690	Putative protein
PDIG_63520	PDIP_72890	Putative protein
PDIG_67200	PDIP_76500	Putative protein
PDIG_67430	PDIP_76730	Putative protein
PDIG_68380	PDIP_77670	Putative protein
PDIG_68400	PDIP_77690	Putative protein
PDIG_68870	PDIP_78150	Putative protein
PDIG_70100	PDIP_79400	Putative protein

PDIG_70220	PDIP_79530	Putative protein
PDIG_77640	PDIP_04750	Putative protein
PDIG_81120	PDIP_29470	*Putative uncharacterized protein [Ortholog: Phaeosphaeria nodorum : Q0V054]*
PDIG_81140	PDIP_29490	*Decarboxylase DEC1 [Ortholog: Verticillium albo-atrum : C9SYL7]*
PDIG_81950	PDIP_30300	*Putative uncharacterized protein [Ortholog: Verticillium albo-atrum : C9SMP8]*
PDIG_82740	PDIP_86530	Tryptophan synthase
PDIG_84030	PDIP_21690	*Putative uncharacterized protein [Ortholog: Phaeosphaeria nodorum : Q0U0D9]*
PDIG_86650	PDIP_32660	Putative protein
PDIG_86700	PDIP_32710	*Predicted protein [Ortholog: Pyrenophora tritici-repentis : B2VUA5]*
PDIG_87400	PDIP_33430	Putative protein
PDIG_90260	PDIP_07190	Putative protein
PDIG_90270	PDIP_07200	Putative protein
PDIG_90740	PDIP_07660	Putative protein
PDIG_90850	PDIP_07770	Putative protein

NO BRH: No best reciprocal hit was foung in the PHI26 strain

Supplementary table 8: Putative horizontal transfer genes from prokaryotes		
***P. digitatum* PHI26**	***P. digitatum* Pd1**	**Putative function**
PDIG_50040	PDIP_19280	Lipase class 2
PDIG_21040	PDIP_23320	ADP-L-glycero-D-manno-heptose-6-epimerase
PDIG_81140	PDIP_29490	Decarboxylase DEC1
PDIG_86690	PDIP_32700	Putative thiosulfate sulfurtransferase

Supplementary table 9: Predicted secondary metabolism related genes				
**Species**	**NRPS**	**PKS**	**DMAT**	**PKS-NRPS HYBRID**
Ajellomyces capsulata	9	2	1	0
Aspergillus carbonarius	25	19	2	5
Aspergillus clavatus	18	17	3	4
Aspergillus flavus	32	24	9	3
Aspergillus fumigatus	18	15	7	1
Aspergillus niger	21	17	1	1
Botrytis fuckeliana	14	22	1	0
Coccidioides immitis	8	11	1	1
Cochliobolus heterostrophus	15	20	3	0
Cryphonectria parasitica	11	27	2	5
Emericella nidulans	21	31	6	1
Fusarium oxysporum	17	11	2	2
Gaeumannomyces graminis	8	15	0	2
Gibberella zeae	21	15	0	1
Glomerella graminicola	12	38	6	4
Magnaporthe grisea	13	24	3	5
Magnaporthe oryzae 70-15	14	26	3	5
Mycosphaerella graminicola	12	13	0	0
Nectria haematococca	12	13	1	1
Neurospora crassa	5	8	1	0
Penicillium chrysogenum	24	23	1	2
Penicillium digitatum PHI26	13	14	1	3
Penicillium marneffei ATCC 18224	19	26	2	1
Phaeosphaeria nodorum	12	18	2	1
Pyrenophora tritici-repentis	18	20	0	1
Sclerotinia sclerotiorum	10	18	1	0
Talaromyces stipitatus	21	39	1	2
Uncinocarpus reesii	12	5	3	1
Verticillium albo-atrum	13	10	0	1

NRPS	Non-Ribosomal Peptide Synthetase
PKS	Polyketide Synthase
DMAT	Dimethyl allyl tryptophan synthase

Supplementary table 10: dN / dS analysis of patulin related genes			
**Gene name**	**Synonymous**	**Non-synonymous**	**Omega**
patB	122	103	0.84
patC	190	143	0.75
patD	58	54	0.93
patF	56	37	0.66
patG	141	76	0.54
patJ	56	65	1.16
patL	259	258	1.00
**Additional analysis**			**Median omega**
Glycolysis related proteins			0.53
One-to-one orthologs			0.82

Supplementary table 11: Putative small CRPs					
**Gene name**	**Scaffolds**	**Start**	**Stop**	**Frame**	**Sequence**
PDIG_31210	Scaffold 33	91663	91908	+	MASGSCRCHCIIYTACTPLTYLVSCHCTTCRKQAGAPYQTWAIFTTDDIRWHVNPTEGQVTNEATRTLCPQCSSTIKAFLA
PDIG_68840	Scaffold 50	3576204	3576602	+	MQITSIAIILFTAMGAVANPIATASDDLDARDVQLSKYGGQCSLKHNTCTYLKGGRNVIVNCGSAANKRCKSDRHHCEYDEHHRRVDCQTPV
PDIG_70730	Scaffold 50	4111344	4111757	-	MKDSLSAVILLSILECLSRLIFSPLPESPCTTPYSPDKQHHYLRLAHRRDIHPAYGVDIYTALDAQNLPQFINSGAKNCQSHPFLMLSVEKMVGGLWLDSKTLGEIERRKDVPS SCRFCSRQSKTKCRVNDIVPHWQ
PdigORF_53727	Scaffold 1	264893	265069	-	MIVSVQHPPGCLCVTMYTAVVSSSAHTDTCTTTIFMDSSCFWFASDGKPSQSHIVQAK
PdigORF_53445	Scaffold 1	283568	283690	+	MSRLTGCESRNRRLCRCLLLLLQQLTPRRCHMVGQCYYIC
PdigORF_90395	Scaffold 11	169285	169578	-	MKGAFKTVLWHLPFTELVFSASGLSARQVRFDCAKPTQGSQDPWVGLNITKHCNLQRSGIFCTEIAKVHTYTCPQLSTWKVYCMDTLRLDYGYTCYT
PdigORF_90437	Scaffold 11	273317	273538	-	MSLLVSCSGFLCLLVFKVIPVWTEGLDIYKYEYICFPCALTLSQSLIFQSSPLFFFLLLSHDIGAGRDMALFT
PdigORF_102414	Scaffold 12	170618	170824	-	MYLSSLFFYFGTSCSAMTFCDISLGCLNRICHPPRLHQFHAKLLRKSLRLSGSLCSSPSILIKHPLVC
PdigORF_111041	Scaffold 19	469429	469515	+	MWITLGFCICHIHGASARACRMVCATKT
PdigORF_55955	Scaffold 2	321333	321491	-	MASLCSSSLERFSLLSEILLVGSTSWSLSLQVCCQRKQRTCSTVPAVDSLKE
PdigORF_56977	Scaffold 2	342361	342459	-	MSCTLSVVLFTPYLCYLFCRDCAYTCDRTFTC
PdigORF_57111	Scaffold 2	349919	350032	-	MSRVTCVTTSCPICGICLGKREIDASVFTRRKTVEDG
PdigORF_54558	Scaffold 2	552596	552787	-	MTPVRQFSSAITRVRTLWMFPRVFTSSRASSRVRYCLFAGMHGCTCTCAWDRDEQVPASSFEM
PdigORF_111713	Scaffold 20	200420	200563	+	MCLLITTLVEHLLSKSSSHALSCTDSPRCTSPCTQEHDKTTRVIMFR
PdigORF_3765	Scaffold 23	87423	87512	-	MGPPFRCKGCLTRCLSALAAVLCYPSFLV
PdigORF_3886	Scaffold 23	347544	347627	+	MARRSPLVIFLCMCHGFIFNMCRCTLE
PdigORF_50949	Scaffold 29	349466	349579	+	MCLFPGCKNIRTECALVILSCIGHLHSDDFISTKLDF
PdigORF_51191	Scaffold 29	425176	425271	-	MEFATLVEMGCLFACQSGANGPEKICGPCGP
PdigORF_52634	Scaffold 30	4444	4593	+	MLLILATAVVVISIERKACIHCSSSDIQNSPDLNDVISSCTFRVRCVLG
PdigORF_24764	Scaffold 31	39307	39435	-	MSLFLPSTCCLVLRAVHEELLCNLQLFCSVSMLFEIDSFSPM
PdigORF_24923	Scaffold 31	310273	310371	+	MGLAWCILLRCIADLAAPAICLCHAQQVDQAA
PdigORF_85491	Scaffold 32	540513	540638	-	MVLFLFTRVLYPGCCCQVFVLAQIRWCLCKRTLIIFQICWE
PdigORF_83860	Scaffold 32	915897	915977	+	MGCLRNSLCPAFLCSSHTRGERVCDQ
PdigORF_85940	Scaffold 32	1010634	1011035	-	MEKLVCVLILPLRHGLSYICMSSKKPAATWRRHQMHFYFSIPIFILSICKLTSPHDHLICSLNISNTRDCAQLAQQRGTSQLNCFANQFDVGDVEFFRQINEACCCGDKIGDEE IVVNRVAVVTAYHANRECV
PdigORF_89251	Scaffold 32	1105916	1106083	+	MHPTHHLVRPPSLASVFSTALCPQCPHPTQAVSHNCFCRRYSAWPRPVLFPTAKL
PdigORF_87691	Scaffold 32	1530898	1530984	-	MTSGCCSGALLICFSLAQSCTAGKKYLA
PdigORF_68422	Scaffold 33	689358	689639	-	MRKRALWSIFSGVNRHSSCGDRVTRVKIGHPSCLCPLGQEATFEEENDNHVESIYLCIAATFTEITKENPESGHNPSISACNAVLHSSMICPI
PdigORF_70940	Scaffold 33	722410	722553	-	MLLFLLLVNSRRGLCCTQVFGLGSNGNCARLSTTCAVDRVLLIAHFM
PdigORF_62353	Scaffold 34	199115	199201	-	MNWGIYCCAGVRRCCAASLSIIKKHANQ
PdigORF_61735	Scaffold 34	319644	319811	-	MVASSRDLWRPCSCCLTVSMFVLFWTCAQMTQHNVPRNIIMEGNHGWEPWMDIES
PdigORF_65543	Scaffold 36	11367	11477	-	MLLVVALGCSQKNDAARCCGPVYSCVQHPPISIFED
PdigORF_93050	Scaffold 38	357400	357591	+	MRRGLAFIFISAGLEEGLRQMQISQHCIAAEPFYCATICTGDCNQNEYKSRESELSESNFNRL
PdigORF_99072	Scaffold 39	79580	79693	-	MSMFCLFACAPCSFVCLSHYMQGFHAGLCPKFKIACV
PdigORF_99626	Scaffold 39	492550	492774	+	MPSVLSPVSCCFLLVPSEKIEEQVSTVYEDCLRLVAQKSPCQLGPSFFLVVFAPNGFSVCRHALCKNTMTLEWI
PdigORF_100976	Scaffold 39	762402	762527	-	MKSGVFRRLLFSCNTCCTSLDCKNVHFIGINVIFWGVSLIF
PdigORF_105377	Scaffold 41	103143	103250	-	MVFSLFCPLFFTSVYSPSPFCDQIFRICCIVKDGA
PdigORF_5325	Scaffold 42	127246	127368	-	MTGQGIGTVLCLCHAATERVSGCLSIECIEGKWSRFPAFR
PdigORF_106377	Scaffold 45	361915	361986	-	MFTCSLYLSGLFCRCCLSYLVDS
PdigORF_74990	Scaffold 5	26526	26696	+	MCYHSCLFPLACCTSVVQGVQITGPLLPKLFFLRPVSSNIYPIQTSISWSHITTTF
PdigORF_73652	Scaffold 5	365341	365490	-	MKCCTGSLAYPYLSLSLTFAWDSTSAWFTFGHCSCLKRLFGWKGASSDL
PdigORF_43334	Scaffold 50	284679	284804	+	MFLALLCLVLCLVPGRRGHDLPVCRCACVFLGSWARLVGVC
PdigORF_43247	Scaffold 50	656334	656468	+	MKNPLLILAMFIGVRCRQVYSTLPGTSTPTIPVSVESGRCGSCC
PdigORF_45973	Scaffold 50	698735	698815	-	MWITYLLVHCLPAACYRRCHYVLCIP
PdigORF_45819	Scaffold 50	789013	789126	-	MTCLHFLHTLVVCSWEVCTCVCCTDVVPRGRSSGDRH
PdigORF_33650	Scaffold 50	1354925	1355062	+	MSLVRCSASGLQGLSACIRSAPGVLGNLERSLSDPSIGYTCQDPC
PdigORF_35147	Scaffold 50	1585110	1585397	+	MTSRRPLSEHIIIGIILLNVLSRNCHASCASNASSAHISCLMDCDALLKIIIHRFRLDCMCHLLKLLCADTPRSASVIHNPTHCLSFFIRSAESL
PdigORF_44313	Scaffold 50	1702099	1702263	+	MVPRGICKRPKSSPLFFSSSPSGVHCDMVRMYNIQHTCTCRCTSSCSFLPRYIR
PdigORF_29232	Scaffold 50	3093700	3093840	-	MPPCLFAMCFRSLLFIGRNVSPGLVLRCIFCFEGCAFWPSVLLVRC

PdigORF_33373	Scaffold 50	3829551	3829811	-	MSLSTWVFCIHGVWTQNLYLHYVGCLFISNLFHECTTFCTGFPFVTSSQAAHIVISFYAGGQPASRRPLVSGARSRKSASHESRGT
PdigORF_26722	Scaffold 50	4315795	4315968	+	MVFIRFYWLISLHLLQCPCIPLYPCRNPTSTSENFIFALLYILADFQPCHSLAYYTD
PdigORF_35205	Scaffold 50	4343978	4344124	-	MFAFGCLGMCIALYYVFSYMSQCVNICLPESCVRRNSAWLTEQNICWK
PdigORF_48793	Scaffold 51	115720	115995	+	MTTSCLLSGCISLTSSQSMALRQSLHTCSFTCGCGMMDLTASQLPSSLYQGIVRNDHLLEKSHCVRFVNDGASSVSGSDTRPNQAAVFGLL
PdigORF_48067	Scaffold 51	285565	285762	+	MSKLGSWMKRGGCTYVVHPAFVVASVSVSGFPSSKNTVCCPTLPFLLCSSDHDFHRGPSSFATTL
PdigORF_49497	Scaffold 51	492002	492169	-	MKKYLARASRILLVSRLVPVLCDFSFKNSPQMSCFCSLYLLPMSCGRGNQVVFML
PdigORF_46847	Scaffold 51	514418	514753	+	MYNGMMLPLTGPKPGRFARRGVLGLLSFAGEGLLSDLGVCPVLTHLLASHERIFSFLIKFSNPSSKLCVCAVIRHFCCHLLRLCFTRCRSSTRIENRCKIQVCCVVTHLFL
PdigORF_46712	Scaffold 51	893926	894111	-	MHLTRLVVFTLFYSAVALPLQHCSTKPSVWCCQALVLQHRTKGLVLRDLSRPRYTCRPWAL
PdigORF_50378	Scaffold 51	950028	950126	+	MLKYQYHPLACLFYLTLYCWPTLCVKCVHPAS
PdigORF_96454	Scaffold 67	7985	8059	-	MTICCTLVRFLGPCMGFHFCDGSV
PdigORF_11312	Scaffold 72	809714	809923	+	MLMHVQLRIPFLLTIPFPEVYCDLTPCVLFFSCSSPSAFVFSAHFPYIVLTTPSGYHIIYYLLSVCHVL
PdigORF_10734	Scaffold 72	976703	976900	+	MSCITSHSSRAYVFLFFGGWGERRVLCHCCPSTNLPFTPISFLSFSLTPRMPLTRLFNTSAWLLS
PdigORF_10750	Scaffold 72	978902	979024	-	MHSFIYAMFNLLCELCHLSWGGHQELCSSGRTDEHHCTIH
PdigORF_11328	Scaffold 72	1122014	1122163	+	MVMDGTSSMLACFLLFSAGQPCSSCRSSPFWGWLVDGTLGTCLNLGIGV
PdigORF_16297	Scaffold 73	370633	370710	-	MQPIPFSSLCVCSSSCRAPWIGCLL
PdigORF_17085	Scaffold 74	3127	3282	+	MSQSTCVFPRKPHNRPRPYCTGFWGLLMTGVSADHGPRRKASYLGCVTCES
PdigORF_18868	Scaffold 75	196085	196348	+	MARQNPLGSDALEAAEKAENHVARGVCQLLLSLPACLACPMTKKSWLTKDPQMEIQVVAVRLALRASLDCISTWLQIATFEIVLRTP
PdigORF_19089	Scaffold 75	408748	408948	+	MSYSILFPTCILSSGFPMGKCHTILWILQPIMSLTLQLCTLPKLGTPSTSKGTILGQLQYVHCRNW
PdigORF_19449	Scaffold 75	423489	423623	-	MTGALSTTLVVMGLLGRPEVWGCKTCGPNCTPYEPCRTYRPIWN
PdigORF_20742	Scaffold 78	502882	503028	-	MDTATKSSVLCISTICLTCVNQTTSVSKMDLSCNIPLLTTVTRRNFVL
PdigORF_77644	Scaffold 8	120175	120261	-	MSIKCPKFCLFSGFWLSWPCRYCRSGSQ
PdigORF_79422	Scaffold 8	207212	207295	-	MKCNLFLCLTLQPSLDCRADCIRTYRQ
PdigORF_77787	Scaffold 8	406203	406358	+	MIYISLASVSCVVLTPFCPEPCRLVVCFTSTQARPYPNFSKGLAIPIPKIF
PdigORF_79798	Scaffold 9	49243	49416	+	MLCWVSPFMMPHLLPLCLKYCITYACLVDACILRREHFPCIQFRSGGSEKRRSLRGS
PdigORF_1017	Scaffold 90	421151	421246	+	MCIQITGCALIASGICLPLPTSLFGKRKICR

Supplementary table 12: Putative LysM proteins		
***P. digitatum* PHI26**	***P. digitatum* Pd1**	**Putative function**
PDIG_25220	PDIP_59710	Putative uncharacterized protein [Ortholog: *Emericella nidulans* : Q5AYG6]
PDIG_25240	PDIP_59730	Predicted protein [Ortholog: *Neurospora crassa* : Q7SG57]
PDIG_23650	PDIP_16060	LysM domain protein [Ortholog: *Aspergillus clavatus* : A1CNR4]
PDIG_42020	PDIP_06030	Putative protein
PDIG_20490	PDIP_00110	Putative uncharacterized protein [Ortholog: *Emericella nidulans* : Q5BA48]
PDIG_22820	PDIP_15220	LysM domain protein, putative [Ortholog: *Aspergillus fumigatus* : Q4W9R4]
PDIG_12980	PDIP_39200	LysM domain protein, putative [Ortholog: *Aspergillus fumigatus* : A4D9P3]

Supplementary table 13: Total Carbohydrate-active enzyme and associated (CAZy) modules									
**Species**	**Glycoside hydrolase**	**Glycosyltransferase**	**Polysaccharide lyase**	**Carbohydrate esterases**	**Carbohydrate-binding module**	**Enzyme modules**	**GH+PL+CE modules**	**Auxilliar modules**	**Total CAZY modules**
Penicillium digitatum PHI26	170	60	7	9	29	246	186	29	275
Penicillium digitatum Pd1	169	60	7	9	30	245	185	30	275
Penicillium chrysogenum	237	78	11	10	47	336	258	47	383
Botrytis cinerea	248	68	16	18	64	350	282	64	414

Supplementary table 14: List of putatively secreted P. digitatum proteins		
***P. digitatum* PHI26**	***P. digitatum* Pd1**	**Annotation**
PDIG_00070	PDIP_02360	*Putative uncharacterized protein [Ortholog: Aspergillus fumigatus : A4DA38]*
PDIG_00130	PDIP_02410	*Putative uncharacterized protein [Ortholog: Magnaporthe grisea : A4REJ2]*
PDIG_00170	PDIP_02450	*Putative uncharacterized protein [Ortholog: Aspergillus fumigatus : Q4WCC7]*
PDIG_00300	PDIP_02560	*Probable beta-glucosidase L [Ortholog: Emericella nidulans : Q5B9F2]*
PDIG_00330	PDIP_02590	*Putative uncharacterized protein [Ortholog: Aspergillus fumigatus : Q4WUP3]*
PDIG_00340	PDIP_02600	*Serine carboxypeptidase (CpdS), putative [Ortholog: Talaromyces stipitatus : B8MPH0]*
PDIG_00380	PDIP_02640	Putative protein
PDIG_00420	PDIP_02680	*Beta-N-acetylhexosaminidase [Ortholog: Penicillium chrysogenum : Q9URR8]*
PDIG_00460	PDIP_02720	Putative protein
PDIG_00570	PDIP_02830	Putative protein
PDIG_00580	PDIP_02840	*Glucose oxidase, putative [Ortholog: Talaromyces stipitatus : B8MDS4]*
PDIG_00610	PDIP_02870	Putative protein
PDIG_00660	PDIP_02920	*Endo-1,4-beta-xylanase [Ortholog: Penicillium chrysogenum : B6F254]*
PDIG_00670	PDIP_02930	*DUF821 domain protein [Ortholog: Aspergillus fumigatus : Q4X047]*
PDIG_00750	PDIP_03010	*Similar to Pc12g01740 protein [Blast hit trembl: Penicillium chrysogenum : B6GZ47]*
PDIG_00760	PDIP_03020	*Putative uncharacterized protein [Ortholog: Emericella nidulans : Q5AZ94]*
PDIG_00770	PDIP_03030	*Oxalate decarboxylase, putative [Ortholog: Aspergillus fumigatus : Q4X060]*
PDIG_00900	PDIP_12220	*Protease S8 tripeptidyl peptidase I, putative [Ortholog: Talaromyces stipitatus : B8MIE2]*
PDIG_01210	PDIP_12530	*Putative uncharacterized protein [Ortholog: Emericella nidulans : Q5B4T4]*
PDIG_01260	PDIP_12580	*1,3-beta-glucanosyltransferase Gel3 [Ortholog: Aspergillus clavatus : A1C6W2]*
PDIG_01380	PDIP_12700	*Similar to Pc22g18570 protein [Blast hit trembl: Penicillium chrysogenum : B6HUM9]*
PDIG_01480	PDIP_12800	*Putative uncharacterized protein [Ortholog: Emericella nidulans : Q5B4V0]*
PDIG_01560	PDIP_12880	*Whole genome shotgun sequence assembly, scaffold_9 [Blast hit trembl: Sordaria macrospora: D1Z8L2]*
PDIG_01660	PDIP_12990	*Cytochrome P450 monooxygenase, putative [Ortholog: Aspergillus fumigatus : Q4WR20]*
PDIG_01700	PDIP_13030	*Aspartyl proteinase [Ortholog: Coccidioides immitis : Q9P8Y4]*
PDIG_01760	PDIP_13090	*Aspartic-type endopeptidase ctsD [Ortholog: Aspergillus fumigatus : Q4WNV0]*
PDIG_01970	PDIP_13300	*Protein ecm33 [Ortholog: Aspergillus fumigatus : Q4WNS8]*
PDIG_02120	PDIP_13450	*Similar to Pc22g17730 protein [Blast hit trembl: Penicillium chrysogenum : B6HT95]*
PDIG_02140	PDIP_13470	*Allergen Asp f 7 [Blast hit: Neosartorya fumigata: O42799]*
PDIG_02170	PDIP_13500	*Acid sphingomyelinase, putative [Ortholog: Aspergillus fumigatus : Q4WNR0]*
PDIG_02340	PDIP_13630	Putative protein
PDIG_02650	PDIP_13910	*Similar to Pc22g17200 protein [Blast hit trembl: Penicillium chrysogenum : B6HSE4]*
PDIG_02660	PDIP_13920	*Similar to Pc22g17190 protein [Blast hit trembl: Penicillium chrysogenum : B6HSE3]*
PDIG_02780	PDIP_14040	*Putative uncharacterized protein [Ortholog: Emericella nidulans : Q5ATD7]*
PDIG_02800	PDIP_14060	*Similar to putative uncharacterized protein Pc22g17040 [Blast hit trembl: Penicillium chrysogenum : B6HSC8]*
PDIG_02840	PDIP_14100	*Protein get1 [Ortholog: Talaromyces stipitatus : B8LUE3]*
PDIG_03040	PDIP_31200	*Histidine acid phosphatase, putative [Ortholog: Talaromyces stipitatus : B8M1R5]*
PDIG_03380	PDIP_08080	*Pectin lyase B [Ortholog: Aspergillus fumigatus : Q4WGH2]*
PDIG_04310	PDIP_08970	*Similar to putative uncharacterized protein Pc20g03320 [Blast hit trembl: Penicillium chrysogenum : B6HG20]*
PDIG_04400	PDIP_09060	*Lipoyl synthase, mitochondrial [Ortholog: Aspergillus fumigatus : Q4WWP1]*
PDIG_04820	PDIP_09490	*Putative uncharacterized protein [Ortholog: Emericella nidulans : Q5B3S3]*
PDIG_04880	PDIP_09550	*Putative uncharacterized protein [Ortholog: Pyrenophora tritici-repentis : B2W8K8]*
PDIG_04960	PDIP_09630	*FAD binding domain protein [Ortholog: Aspergillus clavatus : A1CJI2]*
PDIG_04980	PDIP_09650	*Class V chitinase, putative [Ortholog: Aspergillus fumigatus : A4D9F7]*
PDIG_05040	PDIP_09710	*Polysaccharide deacetylase, putative [Ortholog: Talaromyces stipitatus : B8MMH0]*
PDIG_05050	PDIP_09720	*Putative uncharacterized protein [Ortholog: Aspergillus fumigatus : Q4WWV6]*
PDIG_05130	PDIP_09800	*Putative uncharacterized protein [Ortholog: Aspergillus clavatus : A1CJJ6]*
PDIG_05140	PDIP_09810	*Similar to Pc20g02100 protein [Blast hit trembl: Penicillium chrysogenum : B6HE84]*
PDIG_05300	PDIP_09970	*Phospholipase C PLC-C [Ortholog: Aspergillus fumigatus : Q58I93]*

PDIG_05350	PDIP_10020	*Pectin methylesterase, putative [Ortholog: Aspergillus fumigatus : Q4WWZ5]*
PDIG_05640	PDIP_10310	*Putative uncharacterized protein [Ortholog: Emericella nidulans : Q5AYZ4]*
PDIG_05650	PDIP_10320	*Similar to Pc15g00980 protein [Blast hit trembl: Penicillium chrysogenum : B6H6P2]*
PDIG_06150	PDIP_10830	*Phosphatidylglycerol/phosphatidylinositol transfer protein [Ortholog: Emericella nidulans : Q5B0Q1]*
PDIG_06190	PDIP_10870	*GPI-anchored cell wall beta-1,3-endoglucanase EglC [Ortholog: Talaromyces stipitatus : B8MGA7]*
PDIG_06500	PDIP_11140	*Glucose N-acetyltransferase 1 [Blast hit: Gibberella zeae: Q4HVS2]*
PDIG_06520	PDIP_11160	*Similar to Pc21g22790 protein [Blast hit trembl: Penicillium chrysogenum : B6HNY1]*
PDIG_06770	PDIP_11420	*Putative uncharacterized protein [Ortholog: Aspergillus fumigatus : Q4WRH4]*
PDIG_06820	PDIP_11470	*Probable glycosidase crf1 [Ortholog: Aspergillus fumigatus : Q8J0P4]*
PDIG_06910	PDIP_11560	Putative protein
PDIG_07090	PDIP_11740	*Beta-xylosidase XylA [Ortholog: Talaromyces stipitatus : B8M137]*
PDIG_07140	PDIP_11790	*Polysaccharide deacetylase (NodB), putative [Ortholog: Aspergillus clavatus : A1CB94]*
PDIG_07330	PDIP_11980	*Hydrophobin [Ortholog: Aspergillus fumigatus : Q8J0V5]*
PDIG_07340	PDIP_11990	*Putative uncharacterized protein [Ortholog: Aspergillus clavatus : A1CNA7]*
PDIG_07350	PDIP_12000	*Putative uncharacterized protein [Ortholog: Aspergillus clavatus : A1C541]*
PDIG_08100	PDIP_36120	*Cell surface spherulin 4-like protein, putative [Ortholog: Talaromyces stipitatus : B8MHZ7]*
PDIG_08540	PDIP_36560	*Phospholipase C PLC-B [Ortholog: Aspergillus fumigatus : Q58I94]*
PDIG_08570	PDIP_36590	*Similar to putative uncharacterized protein Pc22g22680 [Blast hit trembl: Penicillium chrysogenum : B6HUP0]*
PDIG_08960	PDIP_36990	Putative protein
PDIG_09080	PDIP_37110	*Putative uncharacterized protein [Ortholog: Emericella nidulans : Q5AZ57]*
PDIG_09510	PDIP_37540	Putative uncharacterized protein [Ortholog: Phaeosphaeria nodorum : Q0UPS1]
PDIG_09890	PDIP_39930	*FAD binding domain protein [Ortholog: Aspergillus clavatus : A1CN67]*
PDIG_09940	PDIP_39980	*FAD binding domain protein [Ortholog: Aspergillus clavatus : A1CN64]*
PDIG_09970	PDIP_40010	*Trypsin-like serine protease, putative [Ortholog: Aspergillus clavatus : A1CN69]*
PDIG_10120	PDIP_40160	*UPF0619 GPI-anchored membrane protein AFUA_3G00880 [Blast hit: Neosartorya fumigata: Q4WFV6]*
PDIG_10280	PDIP_40320	Putative protein
PDIG_10550	PDIP_82060	*Aspartic protease pep1 [Ortholog: Aspergillus fumigatus : P41748]*
PDIG_10750	PDIP_82260	*N-acetylglucosaminyl-phosphatidylinositol deacetylase, putative [Ortholog: Aspergillus fumigatus : Q4WVL6]*
PDIG_10860	PDIP_82370	*Alcohol dehydrogenase [Ortholog: Aspergillus fumigatus : Q4WGA6]*
PDIG_11100	PDIP_82610	*Protein kinase C substrate, putative [Ortholog: Aspergillus fumigatus : Q4WG82]*
PDIG_11270	PDIP_82770	*Acetylxylan esterase, putative [Ortholog: Talaromyces stipitatus : B8LWY3]*
PDIG_11300	PDIP_82800	*Predicted protein [Ortholog: Uncinocarpus reesii : C4K033]*
PDIG_11370	PDIP_82870	*Predicted protein [Ortholog: Emericella nidulans : Q5BB87]*
PDIG_11770	PDIP_38000	Putative protein
PDIG_12160	PDIP_38380	*Chitin deacetylase, putative [Ortholog: Aspergillus fumigatus : Q4WMC1]*
PDIG_12580	PDIP_38800	*Similar to Pc21g20300 protein [Blast hit trembl: Penicillium chrysogenum : B6HKM5]*
PDIG_12980	PDIP_39200	*LysM domain protein, putative [Ortholog: Aspergillus fumigatus : A4D9P3]*
PDIG_13000	PDIP_39220	*Extracellular protein, putative [Ortholog: Aspergillus fumigatus : Q4WM72]*
PDIG_13350	PDIP_40490	*Putative uncharacterized protein [Ortholog: Aspergillus fumigatus : Q4WTE4]*
PDIG_13460	PDIP_40590	*GPI anchored protein, putative [Ortholog: Aspergillus fumigatus : Q4WTD8]*
PDIG_13580	PDIP_47720	*Extracellular endoglucanase, putative [Ortholog: Aspergillus fumigatus : Q4WE56]*
PDIG_14130	PDIP_39780	*Putative uncharacterized protein [Ortholog: Emericella nidulans : Q5BFX1]*
PDIG_14140	PDIP_39790	*1,3-beta-glucanosyltransferase gel2 [Ortholog: Aspergillus fumigatus : P0C954]*
PDIG_14190	PDIP_32120	Putative protein
PDIG_14830	PDIP_02280	Putative protein
PDIG_15020	PDIP_30540	*1,3-beta-glucanosyltransferase Bgt1 [Ortholog: Aspergillus fumigatus : Q4WSV9]*
PDIG_15060	PDIP_30590	*Endosomal cargo receptor (P24), putative [Ortholog: Aspergillus fumigatus : Q4WSV8]*
PDIG_15140	PDIP_30660	*Endopolyphosphatase [Ortholog: Emericella nidulans : Q5BE82]*
PDIG_15240	PDIP_30760	*Putative uncharacterized protein [Ortholog: Aspergillus fumigatus : Q4WSV1]*
PDIG_15450	PDIP_30990	*Putative uncharacterized protein [Ortholog: Talaromyces stipitatus : B8M2N9]*
PDIG_15750	PDIP_31320	*Alpha-ketoglutarate-dependent 2,4-dichlorophenoxyacetate dioxygenase, putative [Blast hit trembl: Penicillium marneffei: B6QPT6]*
PDIG_15790	PDIP_31360	*GPI anchored protein, putative [Ortholog: Aspergillus fumigatus : Q4WT45]*

PDIG_16290	PDIP_87810	Putative protein
PDIG_16510	PDIP_88020	Putative protein
PDIG_16630	PDIP_88140	*Glutaminyl cyclase, putative [Ortholog: Aspergillus fumigatus : Q4WP74]*
PDIG_16690	PDIP_88200	*Putative uncharacterized protein [Ortholog: Emericella nidulans : Q5B9E7]*
PDIG_16910	-	*Protein kinase domain protein [Ortholog: Aspergillus clavatus : A1CRU3]*
PDIG_16940	-	*Similar to Pc13g00960 protein [Blast hit trembl: Penicillium chrysogenum : B6H1A2]*
PDIG_16990	-	*Putative uncharacterized protein [Blast hit trembl: Neosartorya fumigata: Q4WWA4]*
PDIG_17090	PDIP_55010	Putative protein
PDIG_17180	PDIP_55080	*Aspergillopepsin, putaitve [Ortholog: Aspergillus fumigatus : Q4WAG2]*
PDIG_17220	PDIP_55120	Putative protein
PDIG_17590	PDIP_55480	*FAD dependent oxidoreductase, putative [Ortholog: Aspergillus fumigatus : Q4WAJ8]*
PDIG_18790	PDIP_56620	Putative protein
PDIG_18840	PDIP_56670	*NIMA-interacting protein TinC [Ortholog: Aspergillus fumigatus : Q4WAU9]*
PDIG_18940	PDIP_88580	*DOC family protein [Ortholog: Aspergillus fumigatus : Q4WC03]*
PDIG_19060	PDIP_01550	*DnaJ and TPR domain protein [Ortholog: Aspergillus fumigatus : Q4WWD0]*
PDIG_19280	PDIP_01330	*60S ribosomal protein L20, putative [Ortholog: Aspergillus fumigatus : Q4WWD6]*
PDIG_19420	PDIP_01190	*Serum paraoxonase/arylesterase family protein [Ortholog: Aspergillus fumigatus : Q4WWG7]*
PDIG_20200	PDIP_00420	*Putative uncharacterized protein [Ortholog: Aspergillus fumigatus : Q4WF34]*
PDIG_20470	PDIP_00130	*Putative uncharacterized protein [Ortholog: Aspergillus fumigatus : Q4WEZ6]*
PDIG_20490	PDIP_00110	*Putative uncharacterized protein [Ortholog: Emericella nidulans : Q5BA48]*
PDIG_20600	PDIP_88430	*Putative uncharacterized protein [Ortholog: Talaromyces stipitatus : B8MBM4]*
PDIG_20640	PDIP_88450	*Laccase TilA [Ortholog: Aspergillus fumigatus : Q4WRN7]*
PDIG_20830	PDIP_23130	*Putative uncharacterized protein [Ortholog: Uncinocarpus reesii : C4JI16]*
PDIG_21060	PDIP_23340	*1,3-beta-glucanosyltransferase, putative [Ortholog: Aspergillus fumigatus : Q4WYI9]*
PDIG_21170	PDIP_23450	*Aldose 1-epimerase, putative [Ortholog: Talaromyces stipitatus : B8M6K3]*
PDIG_21230	PDIP_23510	*Short-chain oxidoreductase, putative [Ortholog: Aspergillus fumigatus : Q4WS55]*
PDIG_21430	PDIP_23710	Putative protein
PDIG_21630	PDIP_23910	Putative protein
PDIG_21930	PDIP_24210	Putative protein
PDIG_21980	PDIP_24260	*Palmitoyl-protein thioesterase [Ortholog: Aspergillus fumigatus : Q4WYS3]*
PDIG_22070	PDIP_24350	*Rhamnogalacturonan acetylesterase RgaE [Ortholog: Aspergillus fumigatus : Q4WYW6]*
PDIG_22110	PDIP_24390	*Ketol-acid reductoisomerase [Ortholog: Aspergillus fumigatus : Q4WYW4]*
PDIG_22160	PDIP_24440	*Cytochrome c oxidase subunit 7A [Ortholog: Aspergillus fumigatus : Q4WYV9]*
PDIG_22450	PDIP_14840	*Cytochrome c oxidase assembly protein cox16, mitochondrial [Ortholog: Emericella nidulans : Q5AXJ9]*
PDIG_22480	PDIP_14870	*Putative uncharacterized protein [Ortholog: Emericella nidulans : Q5AXJ6]*
PDIG_22660	PDIP_15060	*Putative uncharacterized protein [Ortholog: Emericella nidulans : Q5AX74]*
PDIG_22770	PDIP_15170	*Predicted protein [Ortholog: Uncinocarpus reesii : C4JH88]*
PDIG_22810	PDIP_15210	*Triacylglycerol lipase (LipA), putative [Ortholog: Aspergillus fumigatus : Q4W9R3]*
PDIG_22860	PDIP_15260	Putative protein
PDIG_22870	PDIP_15270	*C6 transcription factor Ctf1A, putative [Ortholog: Aspergillus fumigatus : Q4W9R8]*
PDIG_23080	PDIP_15480	*Aminopeptidase, putative [Ortholog: Talaromyces stipitatus : B8MDS6]*
PDIG_23270	PDIP_15670	*Alanine racemase family protein, putative [Ortholog: Talaromyces stipitatus : B8MDU2]*
PDIG_23410	PDIP_15820	*Putative uncharacterized protein [Ortholog: Aspergillus fumigatus : Q4W9M2]*
PDIG_23520	PDIP_15930	*Putative uncharacterized protein [Ortholog: Emericella nidulans : Q5BG37]*
PDIG_23590	PDIP_16000	*Putative uncharacterized protein [Ortholog: Aspergillus clavatus : A1C990]*
PDIG_23650	PDIP_16060	*LysM domain protein [Ortholog: Aspergillus clavatus : A1CNR4]*
PDIG_23770	PDIP_16180	Putative protein
PDIG_24340	PDIP_58820	*Alpha-1,2-mannosyltransferase, putative [Ortholog: Aspergillus fumigatus : Q4WVR9]*
PDIG_24430	PDIP_58910	*Putative uncharacterized protein [Ortholog: Magnaporthe grisea : A4RKV0]*
PDIG_24920	PDIP_59400	*Protein disulfide-isomerase tigA [Ortholog: Aspergillus niger : Q00216]*
PDIG_25190	PDIP_59680	*Putative uncharacterized protein [Ortholog: Aspergillus fumigatus : Q4WVG3]*
PDIG_25220	PDIP_59710	*Putative uncharacterized protein [Ortholog: Emericella nidulans : Q5AYG6]*

PDIG_25230	PDIP_59720	*Putative uncharacterized protein [Ortholog: Aspergillus fumigatus : Q4WA44]*
PDIG_25670	PDIP_60150	*Putative uncharacterized protein [Ortholog: Emericella nidulans : Q5BH49]*
PDIG_25740	PDIP_60220	*Putative uncharacterized protein [Ortholog: Aspergillus fumigatus : Q4WDU6]*
PDIG_26060	-	Putative protein
PDIG_26280	PDIP_60760	Putative protein
PDIG_26590	PDIP_61040	*Mannan endo-1,4-beta-mannosidase F [Blast hit: Aspergillus clavatus: A1C8U0]*
PDIG_26660	PDIP_61110	*Similar to Pc21g05400 protein [Blast hit trembl: Penicillium chrysogenum : B6HHI6]*
PDIG_26670	PDIP_61120	*Putative uncharacterized protein [Ortholog: Talaromyces stipitatus : B8MB19]*
PDIG_26830	PDIP_61280	Putative protein
PDIG_26920	PDIP_61370	*Putative uncharacterized protein [Ortholog: Aspergillus fumigatus : Q4WCN9]*
PDIG_27040	PDIP_61480	*Putative uncharacterized protein [Ortholog: Aspergillus fumigatus : Q4WCQ0]*
PDIG_27310	PDIP_61750	Putative protein
PDIG_27590	PDIP_62030	*Thioesterase family protein [Ortholog: Aspergillus fumigatus : Q4WCT8]*
PDIG_27790	PDIP_62230	*Putative uncharacterized protein [Ortholog: Talaromyces stipitatus : B8M3V3]*
PDIG_28020	PDIP_62460	*Uncharacterized protein AFUA_6G02800 [Ortholog: Aspergillus fumigatus : Q4WCX9]*
PDIG_28050	PDIP_62490	*Putative uncharacterized protein [Ortholog: Aspergillus fumigatus : Q4WCZ3]*
PDIG_28100	PDIP_62540	*Exopolygalacturonase X [Ortholog: Aspergillus niger : Q27UB3]*
PDIG_28110	PDIP_62550	*Putative uncharacterized protein [Ortholog: Emericella nidulans : Q5AU12]*
PDIG_28250	PDIP_62690	Putative protein
PDIG_28520	PDIP_62960	*Putative uncharacterized protein [Ortholog: Uncinocarpus reesii : C4JL79]*
PDIG_28590	PDIP_63030	*SCP-like extracellular protein, putative [Ortholog: Aspergillus clavatus : A1CPM7]*
PDIG_28610	PDIP_63050	*Cytochrome C1/Cyt1, putative [Ortholog: Aspergillus fumigatus : Q4WKL3]*
PDIG_28800	PDIP_63240	*Similar to putative uncharacterized protein Pc22g21980 [Blast hit trembl: Penicillium chrysogenum : B6HTC4]*
PDIG_29240	PDIP_63670	*ADP-ribosylation factor, putative [Ortholog: Talaromyces stipitatus : B8M8M7]*
PDIG_29880	PDIP_64260	*Iron-sulfur cluster assembly accessory protein Isa2, putative [Ortholog: Aspergillus fumigatus : Q4X1D3]*
PDIG_30080	PDIP_64460	*Polygalacturonase [Blast hit: Penicillium digitatum: Q9Y718]*
PDIG_30110	PDIP_64490	*60S acidic ribosomal protein P2 [Ortholog: Aspergillus fumigatus : Q9UUZ6]*
PDIG_30150	PDIP_64530	*Adhesin, putative [Ortholog: Aspergillus fumigatus : Q4X1F7]*
PDIG_30200	PDIP_64580	Putative protein
PDIG_30630	PDIP_65010	*Predicted protein [Ortholog: Pyrenophora tritici-repentis : B2W519]*
PDIG_30650	PDIP_65030	*GPI anchored cell wall protein, putative [Ortholog: Aspergillus fumigatus : Q4WFZ4]*
PDIG_30830	PDIP_65210	*Extracellular endoglucanase/cellulase, putative [Ortholog: Aspergillus fumigatus : Q4X1L7]*
PDIG_30900	PDIP_50480	*Similar to Pc21g17890 protein [Blast hit trembl: Penicillium chrysogenum : B6HND1]*
PDIG_30940	PDIP_50520	*Similar to Pc21g17930 protein [Blast hit trembl: Penicillium chrysogenum : B6HND5]*
PDIG_30990	PDIP_50570	*Putative uncharacterized protein [Ortholog: Aspergillus fumigatus : Q4WU10]*
PDIG_31210	PDIP_50790	*DUF636 domain protein [Ortholog: Aspergillus fumigatus : Q4WU46]*
PDIG_31290	PDIP_50870	*Hydrophobin, putative [Ortholog: Aspergillus fumigatus : Q4WBR8]*
PDIG_31390	PDIP_50970	*Similar to putative uncharacterized protein Pc21g18510 [Blast hit trembl: Penicillium chrysogenum : B6HHS1]*
PDIG_31520	PDIP_51100	Putative protein
PDIG_31780	PDIP_51360	*Putative uncharacterized protein [Ortholog: Emericella nidulans : Q5B445]*
PDIG_31920	PDIP_51500	*Putative uncharacterized protein [Ortholog: Aspergillus fumigatus : Q4WUK7]*
PDIG_32120	PDIP_51700	*Alpha-1,6-mannosyltransferase subunit (Och1), putative [Ortholog: Aspergillus fumigatus : Q4WUI5]*
PDIG_32140	PDIP_51720	*FAD/FMN-containing isoamyl alcohol oxidase MreA [Ortholog: Aspergillus fumigatus : Q4WD61]*
PDIG_32420	PDIP_52010	Putative protein
PDIG_32570	PDIP_52140	*Similar to Pc22g16030 protein [Blast hit trembl: Penicillium chrysogenum : B6HQP8]*
PDIG_32690	PDIP_52260	*Expansin-like protein 1 [Blast hit: Dictyostelium discoideum: Q55G31]*
PDIG_33000	PDIP_52560	*Putative uncharacterized protein [Ortholog: Aspergillus fumigatus : Q4WUA1]*
PDIG_33090	PDIP_52650	*Probable beta-glucosidase G [Ortholog: Emericella nidulans : Q5B0F4]*
PDIG_33300	PDIP_52880	*Putative uncharacterized protein [Ortholog: Aspergillus fumigatus : Q4WU78]*
PDIG_33420	PDIP_53000	*Similar to Pc22g14870 protein [Blast hit trembl: Penicillium chrysogenum : B6HVC3]*
PDIG_33560	PDIP_53150	*Beta-glucosidase, putative [Ortholog: Aspergillus fumigatus : Q4WU38]*
PDIG_33750	PDIP_53330	*Extracellular serine-threonine rich protein [Ortholog: Aspergillus fumigatus : Q4WYI0]*

PDIG_33760	PDIP_53340	*Hydrophobin, putative [Ortholog: Aspergillus fumigatus : Q4WBR8]*
PDIG_33980	PDIP_53560	*Long chronological lifespan protein 2 [Ortholog: Aspergillus fumigatus : Q4WES5]*
PDIG_34000	PDIP_53580	*Putative uncharacterized protein [Ortholog: Emericella nidulans : Q5AWG1]*
PDIG_34120	PDIP_53700	*Putative transcriptional activator [Ortholog: Emericella nidulans : Q8X1W4]*
PDIG_34290	PDIP_53850	Putative protein
PDIG_34320	PDIP_53880	*Putative uncharacterized protein [Ortholog: Emericella nidulans : Q5B0I6]*
PDIG_34340	PDIP_53900	*Endochitinase 2 [Blast hit: Coccidioides posadasii: P54197]*
PDIG_34360	PDIP_53920	*Putative uncharacterized protein [Ortholog: Aspergillus fumigatus : Q4W9T1]*
PDIG_34550	PDIP_54110	Putative protein
PDIG_34560	PDIP_54130	*Beta-1,6-glucanase Neg1, putative [Ortholog: Aspergillus fumigatus : Q4WBR2]*
PDIG_34840	PDIP_54410	*Putative uncharacterized protein [Ortholog: Aspergillus clavatus : A1CG66]*
PDIG_34910	PDIP_54470	*Nuclease PA3, putative [Ortholog: Talaromyces stipitatus : B8MCF5]*
PDIG_34980	PDIP_54540	*SUN domain protein (Uth1), putative [Ortholog: Aspergillus fumigatus : Q4WGL5]*
PDIG_36010	PDIP_05470	*Similar to Pc06g00720 protein [Blast hit trembl: Penicillium chrysogenum : B6GW15]*
PDIG_36020	PDIP_05460	*Hsp70 family chaperone Lhs1/Orp150, putative [Ortholog: Aspergillus fumigatus : Q4WRU6]*
PDIG_36090	PDIP_05390	Putative uncharacterized protein [Ortholog: Phaeosphaeria nodorum : Q0UK59]
PDIG_36170	PDIP_05310	*Ribonuclease T2, putative [Ortholog: Aspergillus fumigatus : Q4WRE3]*
PDIG_36190	PDIP_05290	Putative protein
PDIG_36250	PDIP_05230	*Similar to Pc09g00020 protein [Blast hit trembl: Penicillium chrysogenum : B6GWJ9]*
PDIG_36260	PDIP_05220	Putative protein
PDIG_36270	PDIP_05210	*Aspergillopepsin, putative [Ortholog: Talaromyces stipitatus : B8M952]*
PDIG_36290	-	*Putative uncharacterized protein [Blast hit trembl: Neosartorya fumigata: Q4WBZ1]*
PDIG_36510	PDIP_83110	Putative protein
PDIG_36540	PDIP_83140	*Purine nucleoside permease, putative [Ortholog: Aspergillus fumigatus : Q4WIS5]*
PDIG_36550	PDIP_83150	*Extracellular GDSL-like lipase/acylhydrolase, putative [Ortholog: Aspergillus fumigatus : Q4WIS4]*
PDIG_36700	PDIP_83290	Putative protein
PDIG_36940	PDIP_83530	*Similar to Pc22g25340 protein [Blast hit trembl: Penicillium chrysogenum : B6HS54]*
PDIG_37020	PDIP_83610	*Similar to putative uncharacterized protein Pc22g25460 [Blast hit trembl: Penicillium chrysogenum : B6HSI4]*
PDIG_37060	PDIP_83650	Putative protein
PDIG_37220	PDIP_83810	*Putative uncharacterized protein [Ortholog: Aspergillus clavatus : A1CDP2]*
PDIG_37480	PDIP_84070	*PDI related protein A [Ortholog: Aspergillus niger : O93914]*
PDIG_37500	PDIP_84090	*Capsular associated protein, putative [Ortholog: Aspergillus fumigatus : Q4WJP1]*
PDIG_37510	PDIP_84100	*Endo-1,3(4)-beta-glucanase, putative [Ortholog: Aspergillus fumigatus : Q4WJP2]*
PDIG_37540	PDIP_84130	*Putative uncharacterized protein [Ortholog: Aspergillus fumigatus : Q4WJP4]*
PDIG_37820	PDIP_84410	*Mitochondrial inner membrane nuclease Nuc1, putative [Ortholog: Aspergillus fumigatus : Q4X1U4]*
PDIG_37880	PDIP_84460	*Trafficking protein particle complex subunit 2/Sedlin, putative [Ortholog: Aspergillus fumigatus : Q4X1U7]*
PDIG_38140	PDIP_84720	*Carboxylesterase, putative [Ortholog: Aspergillus fumigatus : Q4X1W9]*
PDIG_38220	PDIP_84850	*Putative uncharacterized protein [Ortholog: Emericella nidulans : Q5B836]*
PDIG_38490	PDIP_85130	*DnaJ domain protein, putative [Ortholog: Aspergillus fumigatus : Q4X1Y9]*
PDIG_38650	PDIP_85290	Putative protein
PDIG_38900	PDIP_85520	Putative protein
PDIG_38960	-	*Similar to Pc13g05030 protein [Blast hit trembl: Penicillium chrysogenum : B6H2E3]*
PDIG_39000	PDIP_46920	*Endonuclease/exonuclease/phosphatase family [Ortholog: Coccidioides posadasiiVerticillium albo-atrum : C9SNJ1]*
PDIG_39120	PDIP_24690	*Cytochrome P450 alkane hydroxylase, putative [Ortholog: Aspergillus fumigatus : Q4WMW7]*
PDIG_39160	PDIP_24730	*Putative uncharacterized protein [Ortholog: Emericella nidulans : Q5B6A8]*
PDIG_39260	PDIP_24830	*Potential peptidyl-tRNA hydrolase [Ortholog: Uncinocarpus reesii : C4JRQ7]*
PDIG_39410	PDIP_24960	*Oxidoreductase, short-chain dehydrogenase/reductase family [Ortholog: Aspergillus fumigatus : Q4WKT7]*
PDIG_39740	PDIP_25280	*Predicted protein [Ortholog: Emericella nidulans : Q5B220]*
PDIG_40020	PDIP_25550	*Putative uncharacterized protein [Ortholog: Emericella nidulans : Q5BC75]*
PDIG_40580	PDIP_26130	*Putative uncharacterized protein [Ortholog: Aspergillus fumigatus : Q4WEV0]*
PDIG_40780	PDIP_85700	*Putative uncharacterized protein [Ortholog: Emericella nidulans : Q5B717]*
PDIG_41280	PDIP_86200	*Putative uncharacterized protein [Blast hit trembl: Aspergillus niger: A2QSS8]*

PDIG_41310	PDIP_86230	*Similar to Pc21g03770 protein [Blast hit trembl: Penicillium chrysogenum : B6HLI7]*
PDIG_41570	PDIP_06490	*Putative uncharacterized protein [Blast hit trembl: Schizosaccharomyces japonicus: B6JUW3]*
PDIG_41710	PDIP_06350	*Similar to Pc16g05180 protein [Blast hit trembl: Penicillium chrysogenum : B6H9H2]*
PDIG_42020	PDIP_06030	Putative protein
PDIG_42030	PDIP_06020	*Aspergillopepsin, putative [Ortholog: Aspergillus fumigatus : Q4WF96]*
PDIG_42070	PDIP_40690	*Putative uncharacterized protein [Ortholog: Emericella nidulans : Q5AQW3]*
PDIG_42110	PDIP_40730	*Fungal specific transcription factor, putative [Ortholog: Aspergillus fumigatus : Q4WZN6]*
PDIG_42380	PDIP_40960	*Putative uncharacterized protein [Ortholog: Emericella nidulans : Q5BF41]*
PDIG_42400	PDIP_40980	*GPI anchored serine-threonine rich protein [Ortholog: Aspergillus fumigatus : Q4WTF2]*
PDIG_42450	PDIP_41030	*Putative uncharacterized protein [Ortholog: Aspergillus fumigatus : Q4WBI9]*
PDIG_42710	PDIP_41290	*Transmembrane glycoprotein, putative [Ortholog: Aspergillus fumigatus : Q4WTH6]*
PDIG_43180	PDIP_34400	*Extracellular metalloprotease AFUA_1G07730 [Ortholog: Aspergillus fumigatus : Q4WJ01]*
PDIG_43440	PDIP_34670	*Putative uncharacterized protein [Ortholog: Aspergillus fumigatus : Q4WJ24]*
PDIG_43880	PDIP_35110	Putative protein
PDIG_44220	PDIP_35450	*Endo alpha-1,4 polygalactosaminidase, putative [Ortholog: Talaromyces stipitatus : B8MMA0]*
PDIG_44250	PDIP_35480	*Fungal chitosanase, putative [Ortholog: Aspergillus clavatus : A1CBX0]*
PDIG_44760	PDIP_16750	*Similar to Pc20g13710 protein [Blast hit trembl: Penicillium chrysogenum : B6HH08]*
PDIG_44860	PDIP_16850	*IgE binding protein, putative [Ortholog: Aspergillus fumigatus : Q4WKM2]*
PDIG_44930	PDIP_16910	*Putative uncharacterized protein [Ortholog: Magnaporthe grisea : A4R6V7]*
PDIG_44990	PDIP_16950	*Isoamyl alcohol oxidase, putative [Ortholog: Aspergillus fumigatus : A4D9R5]*
PDIG_45060	PDIP_17000	Putative protein
PDIG_45070	PDIP_17010	*Polygalacturonase-2 [Ortholog: Coccidioides posadasiiVerticillium albo-atrum : C9SU11]*
PDIG_45920	PDIP_17850	*Alpha-1,2-mannosyltransferase (Mnn2), putative [Ortholog: Aspergillus fumigatus : Q4WDE3]*
PDIG_45940	PDIP_17870	*Predicted protein [Ortholog: Uncinocarpus reesii : C4JFW2]*
PDIG_45990	PDIP_17920	*Putative uncharacterized protein [Ortholog: Aspergillus fumigatus : Q4W8Z4]*
PDIG_46210	PDIP_18140	*Putative uncharacterized protein [Ortholog: Aspergillus fumigatus : Q4WDG7]*
PDIG_46430	PDIP_18350	Putative protein
PDIG_47070	PDIP_19000	Putative protein
PDIG_47260	PDIP_16440	*Ergosterol biosynthesis protein Erg28, putative [Ortholog: Aspergillus fumigatus : Q4X116]*
PDIG_47500	PDIP_56880	*Protein disulfide isomerase [Ortholog: Aspergillus niger : Q5U9X2]*
PDIG_47590	PDIP_56970	*Putative uncharacterized protein [Ortholog: Emericella nidulans : Q5BDF8]*
PDIG_47620	PDIP_57000	*Triacylglycerol lipase, putative [Ortholog: Aspergillus fumigatus : Q4WCJ4]*
PDIG_47640	PDIP_57020	*Putative uncharacterized protein [Ortholog: Aspergillus fumigatus : Q4WCJ2]*
PDIG_47670	PDIP_57050	*Translocation protein (Sec66), putative [Ortholog: Aspergillus fumigatus : Q4WCI9]*
PDIG_47800	PDIP_57180	Putative protein
PDIG_47890	-	Putative protein
PDIG_47980	PDIP_57360	*ABC-type Fe3+ transport system [Ortholog: Pyrenophora tritici-repentis : B2W446]*
PDIG_48090	PDIP_57470	*Alpha-1,2-mannosyltransferase (Kre2), putative [Ortholog: Aspergillus fumigatus : Q4WV44]*
PDIG_48170	PDIP_57550	*Putative uncharacterized protein [Ortholog: Emericella nidulans : Q5B7B1]*
PDIG_48400	PDIP_57780	*Amidase family protein [Ortholog: Talaromyces stipitatus : B8MAL2]*
PDIG_48410	PDIP_57790	*Pectin lyase, putative [Ortholog: Aspergillus clavatus : A1CAZ3]*
PDIG_48420	PDIP_57800	*Sexual development protein (LsdA), putative [Ortholog: Aspergillus fumigatus : Q4WV12]*
PDIG_48480	PDIP_57850	*Putative uncharacterized protein [Ortholog: Pyrenophora tritici-repentis : B2WMN7]*
PDIG_48640	PDIP_58020	*Glycosyl hydrolase, putative [Ortholog: Aspergillus fumigatus : Q4WIT8]*
PDIG_48720	PDIP_58100	*Alpha-1,2-mannosidase, putative [Ortholog: Aspergillus fumigatus : Q4WGE1]*
PDIG_48730	PDIP_58110	*FAD dependent sulfhydryl oxidase Erv2, putative [Ortholog: Aspergillus fumigatus : Q4WGD8]*
PDIG_48980	PDIP_58360	Putative protein
PDIG_49410	PDIP_42280	*O-methyltransferase, putative [Ortholog: Aspergillus fumigatus : Q4WQW4]*
PDIG_49540	PDIP_42400	Putative protein
PDIG_49550	PDIP_42410	*Predicted protein [Ortholog: Aspergillus clavatus : A1C8F5]*
PDIG_49580	PDIP_42440	*Lipase, putative [Ortholog: Aspergillus fumigatus : Q4WGG4]*
PDIG_49610	PDIP_42470	*Extracellular lipase, putative [Ortholog: Aspergillus fumigatus : Q4WBJ7]*

PDIG_49640	PDIP_42500	*Putative uncharacterized protein [Ortholog: Emericella nidulans : Q5AS26]*
PDIG_49660	PDIP_42520	Putative protein
PDIG_49750	PDIP_42610	Putative protein
PDIG_49830	PDIP_42690	*EPD1 [Ortholog: Uncinocarpus reesii : C4JSL9]*
PDIG_49910	PDIP_19140	Putative protein
PDIG_50040	PDIP_19280	*Putative uncharacterized protein [Ortholog: Talaromyces stipitatus : B8MJY1]*
PDIG_50070	PDIP_19310	Putative protein
PDIG_50080	PDIP_19320	*Allergen Asp f 4 [Blast hit: Neosartorya fumigata: O60024]*
PDIG_50090	PDIP_19330	Putative protein
PDIG_50230	PDIP_19470	Putative protein
PDIG_50340	PDIP_19580	*Rhamnogalacturonase, putative [Ortholog: Talaromyces stipitatus : B8M540]*
PDIG_50670	PDIP_19910	Endopolygalacturonase 1 [Ortholog: Botrytis fuckeliana : Q4G496]
PDIG_50750	PDIP_19970	Putative protein
PDIG_51160	PDIP_20380	*Putative uncharacterized protein [Ortholog: Aspergillus fumigatus : Q4WHI9]*
PDIG_51550	PDIP_20750	Putative protein
PDIG_51800	PDIP_21000	Putative protein
PDIG_51850	PDIP_21050	Putative protein
PDIG_52680	-	Putative protein
PDIG_52750	PDIP_47970	*Metallo-beta-lactamase superfamily protein [Ortholog: Aspergillus clavatus : A1C597]*
PDIG_53310	PDIP_48530	*Similar to Pc21g15430 protein [Blast hit trembl: Penicillium chrysogenum : B6HJU1]*
PDIG_53520	PDIP_48740	*Class III chitinase, putative [Ortholog: Aspergillus fumigatus : Q4WEM4]*
PDIG_53840	PDIP_49060	*Putative uncharacterized protein [Ortholog: Aspergillus fumigatus : Q4WEK3]*
PDIG_53870	PDIP_49090	*Putative uncharacterized protein [Ortholog: Aspergillus clavatus : A1C510]*
PDIG_53930	PDIP_49150	*Microsomal signal peptidase subunit (Gp23), putative [Ortholog: Aspergillus fumigatus : Q4WEJ4]*
PDIG_54070	PDIP_49290	*Conidial pigment biosynthesis oxidase Abr1/brown 1 [Ortholog: Aspergillus fumigatus : Q4WZB4]*
PDIG_54160	PDIP_49380	*Putative uncharacterized protein [Ortholog: Aspergillus fumigatus : Q4WEH3]*
PDIG_54480	PDIP_49700	*Vacuolar serine protease [Ortholog: Penicillium chrysogenum : Q9HF04]*
PDIG_54540	PDIP_49760	Putative protein
PDIG_54550	-	*Similar to Pc21g10540 protein [Blast hit trembl: Penicillium chrysogenum : B6HIY9]*
PDIG_54560	PDIP_49770	Putative protein
PDIG_54580	PDIP_49790	Putative protein
PDIG_54760	PDIP_49970	*Sad1/UNC domain protein [Ortholog: Aspergillus fumigatus : Q4WTX9]*
PDIG_54960	-	*Antigenic thaumatin domain protein, putative [Ortholog: Aspergillus fumigatus : Q4WBB5]*
PDIG_54970	PDIP_50180	*Triglyceride lipase-cholesterol esterase, putative [Ortholog: Aspergillus fumigatus : Q4WUM3]*
PDIG_55000	PDIP_50210	*NADH-ubiquinone oxidoreductase 9.5 kDa subunit, putative [Ortholog: Aspergillus fumigatus : Q4WUM5]*
PDIG_55140	PDIP_50350	*Putative uncharacterized protein [Ortholog: Talaromyces stipitatus : B8MD53]*
PDIG_55370	PDIP_14580	*C6 finger domain protein, putative [Ortholog: Talaromyces stipitatus : B8MDH5]*
PDIG_55980	-	*Similar to Pc16g14670 protein [Blast hit trembl: Penicillium chrysogenum : B6HA13]*
PDIG_56000	PDIP_65550	*Putative uncharacterized protein [Ortholog: Aspergillus clavatus : A1C5M6]*
PDIG_56270	PDIP_65830	Putative protein
PDIG_56350	PDIP_65910	*Putative uncharacterized protein [Ortholog: Aspergillus fumigatus : A4D9Z1]*
PDIG_56590	PDIP_66150	*Similar to putative uncharacterized protein Pc16g13760 [Blast hit trembl: Penicillium chrysogenum : B6HAR7]*
PDIG_56860	PDIP_66420	*Mannosyl-oligosaccharide 1,2-alpha-mannosidase [Ortholog: Uncinocarpus reesii : C4JY92]*
PDIG_56880	PDIP_66440	Putative protein
PDIG_57060	PDIP_66600	*Exo-1,3-beta-D-glucanase, putative [Ortholog: Aspergillus fumigatus : Q6MY43]*
PDIG_57120	PDIP_66660	*Possible secreted cellulose-binding protein [Ortholog: Aspergillus fumigatus : Q6MY45]*
PDIG_57130	PDIP_66670	Putative protein
PDIG_57310	PDIP_66850	*Conserved fungal protein [Ortholog: Aspergillus fumigatus : Q4WS27]*
PDIG_57580	PDIP_67090	*Transcription factor spt3, putative [Ortholog: Aspergillus fumigatus : Q6MY90]*
PDIG_57670	PDIP_67180	*SUN family protein, putative [Ortholog: Aspergillus fumigatus : Q6MYA0]*
PDIG_57680	PDIP_67190	*Leucine carboxyl methyltransferase superfamily [Ortholog: Talaromyces stipitatus : B8M0T0]*
PDIG_57940	PDIP_67440	*Allergen, putative [Ortholog: Aspergillus fumigatus : Q4WF99]*

PDIG_57950	PDIP_67450	*Putative uncharacterized protein [Ortholog: Magnaporthe grisea : A4QWI6]*
PDIG_57980	PDIP_46070	Putative protein
PDIG_58090	PDIP_67580	*Catalase B [Ortholog: Emericella nidulans : P78619]*
PDIG_58170	PDIP_67660	*Cytochrome P450 alkane hydroxylase, putative [Ortholog: Aspergillus fumigatus : Q4W9T4]*
PDIG_58200	PDIP_67690	Putative uncharacterized protein [Ortholog: Phaeosphaeria nodorum : Q0UQL8]
PDIG_58250	PDIP_67740	*Similar to Pc14g00510 protein [Blast hit trembl: Penicillium chrysogenum : B6H5N4]*
PDIG_58390	-	*Uncharacterized protein YHR202W [Blast hit: Saccharomyces cerevisiae: P38887]*
PDIG_58650	PDIP_68130	*Pheromone [Ortholog: Penicillium chrysogenum : B1GVB6]*
PDIG_58830	PDIP_68290	*Extracellular triacylglycerol lipase, putative [Ortholog: Aspergillus fumigatus : Q4WNF9]*
PDIG_59000	PDIP_68450	*Beta-galactosidase, putative [Ortholog: Talaromyces stipitatus : B8MKF6]*
PDIG_59010	-	*Ankyrin repeat domain protein, putative [Ortholog: Talaromyces stipitatus : B8M9T6]*
PDIG_59070	PDIP_68510	*Similar to Pc14g01640 protein [Blast hit trembl: Penicillium chrysogenum : B6H5Z2]*
PDIG_59180	PDIP_68620	*Peptidase, putative [Ortholog: Aspergillus fumigatus : Q4WNC9]*
PDIG_59450	PDIP_68890	*Arabinogalactan endo-1,4-beta-galactosidase GalA [Ortholog: Talaromyces stipitatus : B8MTD9]*
PDIG_59490	PDIP_68930	*60S acidic ribosomal protein P1 [Ortholog: Aspergillus fumigatus : Q9HGV0]*
PDIG_59520	PDIP_68960	*Protein phophatase 2C family protein [Ortholog: Aspergillus fumigatus : Q4WJ85]*
PDIG_59540	PDIP_68980	*Alpha-1,2-mannosyltransferase (Alg11), putative [Ortholog: Aspergillus fumigatus : Q4WJ82]*
PDIG_59770	PDIP_69190	*Similar to Pc13g02730 protein [Blast hit trembl: Penicillium chrysogenum : B6H1E0]*
PDIG_59950	PDIP_69370	*Similar to Pc13g02530 protein [Blast hit trembl: Penicillium chrysogenum : B6H1K5]*
PDIG_60120	-	*Similar to Pc13g02350 protein [Blast hit trembl: Penicillium chrysogenum : B6H1H7]*
PDIG_60290	PDIP_69700	Putative protein
PDIG_60350	PDIP_69760	Putative protein
PDIG_60430	PDIP_69840	*Alpha-L-rhamnosidase B, putative [Ortholog: Aspergillus fumigatus : Q4WJF8]*
PDIG_60440	PDIP_69850	*Extracellular exo-polygalacturonase, putative [Ortholog: Aspergillus fumigatus : Q4WJF7]*
PDIG_61070	PDIP_70500	*Cellulose-binding GDSL lipase/acylhydrolase, putative [Ortholog: Talaromyces stipitatus : B8M2U4]*
PDIG_61080	PDIP_70510	*Endo-beta-1,4-glucanase B [Ortholog: Aspergillus niger : O74706]*
PDIG_61150	PDIP_70580	*Putative uncharacterized protein [Ortholog: Coccidioides posadasiiVerticillium albo-atrum : C9SF83]*
PDIG_61260	PDIP_70690	Putative protein
PDIG_61570	PDIP_71000	*Exoglucanase type C [Ortholog: Gibberella zeae : Q6XZE4]*
PDIG_61630	PDIP_71060	*Mitochondrial import inner membrane translocase subunit tim14 [Ortholog: Emericella nidulans : Q5B4H1]*
PDIG_61740	PDIP_71150	*Putative uncharacterized protein [Ortholog: Emericella nidulans : Q5B6L2]*
PDIG_61880	PDIP_71270	*Allergen Asp f 4 [Blast hit: Neosartorya fumigata: O60024]*
PDIG_61930	PDIP_71320	*FKBP-type peptidyl-prolyl isomerase, putative [Ortholog: Aspergillus clavatus : A1CES3]*
PDIG_61940	PDIP_71330	*Heme/steroid binding protein, putative [Ortholog: Aspergillus fumigatus : Q4WHX3]*
PDIG_62200	PDIP_71590	*Carboxypeptidase 5 [Ortholog: Aspergillus fumigatus : Q5VJG7]*
PDIG_62260	PDIP_71640	*Antigen 1 [Ortholog: Uncinocarpus reesii : C4JGA4]*
PDIG_62500	PDIP_71880	*Putative uncharacterized protein [Ortholog: Emericella nidulans : Q5B4L5]*
PDIG_62520	PDIP_71900	*Similar to Pc18g04210 protein [Blast hit trembl: Penicillium chrysogenum : B6HBI5]*
PDIG_62750	PDIP_72130	*Putative uncharacterized protein [Ortholog: Emericella nidulans : Q5B4M6]*
PDIG_62940	PDIP_72320	*Putative uncharacterized protein [Ortholog: Emericella nidulans : Q5BCB3]*
PDIG_63030	PDIP_72410	*Putative uncharacterized protein [Ortholog: Aspergillus fumigatus : A4D9L5]*
PDIG_63100	PDIP_72480	*Cytochrome c peroxidase Ccp1, putative [Ortholog: Aspergillus clavatus : A1CHM3]*
PDIG_63370	-	*GPI anchored cell wall protein, putative [Ortholog: Talaromyces stipitatus : B8M636]*
PDIG_63610	PDIP_72960	*Similar to Pc18g02930 protein [Blast hit trembl: Penicillium chrysogenum : B6HB62]*
PDIG_63640	PDIP_72990	*Lysophospholipase 1 [Ortholog: Aspergillus fumigatus : P0C957]*
PDIG_63800	PDIP_73150	*Similar to Pc18g02680 protein [Blast hit trembl: Penicillium chrysogenum : B6HCY9]*
PDIG_63850	PDIP_73200	*Exo-beta-1,3-glucanase (Exg1), putative [Ortholog: Talaromyces stipitatus : B8MFD7]*
PDIG_63880	PDIP_73230	*Putative uncharacterized protein [Ortholog: Magnaporthe grisea : A4REV5]*
PDIG_63950	PDIP_73300	*Putative uncharacterized protein [Ortholog: Neurospora crassa : Q7RZS2]*
PDIG_64230	PDIP_73570	*Similar to putative uncharacterized protein Pc18g01790 [Blast hit trembl: Penicillium chrysogenum : B6HCK0]*
PDIG_64290	PDIP_73630	*Exo-arabinanase [Ortholog: Penicillium chrysogenum : Q7ZA77]*
PDIG_64510	PDIP_73850	*Capsule-associated protein CAP1, putative [Ortholog: Aspergillus fumigatus : A4D9T1]*

PDIG_64640	PDIP_73980	*Flavin containing polyamine oxidase, putative [Ortholog: Aspergillus fumigatus : Q4WUU7]*
PDIG_64930	PDIP_74260	*Similar to Pc18g01380 protein [Blast hit trembl: Penicillium chrysogenum : B6HCA9]*
PDIG_65090	PDIP_74420	*Signal peptidase I [Ortholog: Aspergillus fumigatus : Q4WYF4]*
PDIG_65350	PDIP_74680	*Glutaminase, putative [Ortholog: Aspergillus fumigatus : Q4WXW3]*
PDIG_66010	PDIP_75310	*Oxidoreductase, putative [Ortholog: Talaromyces stipitatus : B8M818]*
PDIG_66050	PDIP_75350	Putative protein
PDIG_66660	PDIP_75960	*Prenylcysteine lyase, putative [Ortholog: Aspergillus fumigatus : Q4WXF5]*
PDIG_66890	PDIP_76190	*Extracellular thaumatin domain protein, putative [Ortholog: Aspergillus fumigatus : Q4WXJ1]*
PDIG_67040	PDIP_76340	*Putative uncharacterized protein [Ortholog: Talaromyces stipitatus : B8LUR9]*
PDIG_67220	PDIP_76520	*Putative uncharacterized protein [Ortholog: Talaromyces stipitatus : B8LUQ8]*
PDIG_67240	PDIP_76540	*Putative uncharacterized protein [Ortholog: Aspergillus fumigatus : Q4WL81]*
PDIG_67620	PDIP_76920	*Putative uncharacterized protein [Ortholog: Aspergillus clavatus : A1CP92]*
PDIG_67660	PDIP_76960	*Phosphate-repressible acid phosphatase [Ortholog: Penicillium chrysogenum : P37274]*
PDIG_67710	PDIP_77010	Putative protein
PDIG_67760	PDIP_77070	*Putative uncharacterized protein [Ortholog: Emericella nidulans : Q5AX32]*
PDIG_67780	PDIP_77090	*Tripeptidyl-peptidase sed2 [Ortholog: Aspergillus fumigatus : Q70J59]*
PDIG_67820	PDIP_77130	*Predicted protein [Ortholog: Emericella nidulans : Q5BCB7]*
PDIG_67880	PDIP_77180	*Similar to putative uncharacterized protein Pc12g14560 [Blast hit trembl: Penicillium chrysogenum : B6H127]*
PDIG_67970	PDIP_77270	*Putative uncharacterized protein [Ortholog: Emericella nidulans : Q5BCK7]*
PDIG_68100	PDIP_77390	*Putative uncharacterized protein [Ortholog: Emericella nidulans : Q5BAF7]*
PDIG_68540	PDIP_77830	*NPP1 domain protein, putative [Ortholog: Aspergillus fumigatus : Q4WDT6]*
PDIG_68640	PDIP_77930	*Putative uncharacterized protein [Ortholog: Aspergillus fumigatus : Q4W9C5]*
PDIG_68790	PDIP_78070	*GPI anchored protein, putative [Ortholog: Aspergillus fumigatus : Q4WA89]*
PDIG_68840	PDIP_78120	*Antifungal protein Afp [Ortholog: Aspergillus clavatus : A1CSS4]*
PDIG_68890	PDIP_78170	*Related to mixed-linked glucanase MLG1 [Ortholog: Neurospora crassa : Q9P6T0]*
PDIG_69010	PDIP_78300	*6-hydroxy-D-nicotine oxidase [Ortholog: Pyrenophora tritici-repentis : B2VT97]*
PDIG_69070	PDIP_78360	*Similar to putative uncharacterized protein Pc12g12930 [Blast hit trembl: Penicillium chrysogenum : B6GZA9]*
PDIG_69610	PDIP_78900	*Malate dehydrogenase [Ortholog: Aspergillus fumigatus : Q4X0E0]*
PDIG_69650	PDIP_78940	Predicted protein [Blast hit trembl: Ajellomyces capsulata: A6R4R3]
PDIG_70320	PDIP_79630	*RING finger domain protein, putative [Ortholog: Talaromyces stipitatus : B8MRM7]*
PDIG_70440	PDIP_79750	Putative protein
PDIG_70560	PDIP_79870	*Putative uncharacterized protein [Ortholog: Emericella nidulans : Q5AVZ6]*
PDIG_70700	PDIP_80020	*78 kDa glucose-regulated protein homolog [Ortholog: Aspergillus niger : P83616]*
PDIG_70730	PDIP_80050	*Similar to putative uncharacterized protein Pc12g10760 [Blast hit trembl: Penicillium chrysogenum : B6H0Z9]*
PDIG_70770	PDIP_80090	Putative protein
PDIG_70870	PDIP_80190	*Putative uncharacterized protein [Ortholog: Emericella nidulans : Q5AY57]*
PDIG_71110	PDIP_80430	*Similar to putative uncharacterized protein Pc21g01840 [Blast hit trembl: Penicillium chrysogenum : B6HJ80]*
PDIG_71340	PDIP_80640	Putative protein
PDIG_71440	PDIP_80740	*HET-C domain protein HetC [Ortholog: Aspergillus fumigatus : Q4WZT4]*
PDIG_71660	PDIP_80960	Putative protein
PDIG_71690	PDIP_80990	*Isoamyl alcohol oxidase, putative [Ortholog: Aspergillus fumigatus : Q4WAD8]*
PDIG_71860	PDIP_81130	*Predicted protein [Ortholog: Magnaporthe grisea : A4R1S4]*
PDIG_72240	PDIP_81510	*Endo-1,3(4)-beta-glucanase, putative [Ortholog: Aspergillus fumigatus : Q4WW10]*
PDIG_72260	PDIP_81530	*Oxidoreductase, short chain dehydrogenase/reductase family [Ortholog: Aspergillus fumigatus : Q4WW07]*
PDIG_72850	PDIP_43330	*ADP-ribosylglycohydrolase family protein [Ortholog: Aspergillus fumigatus : Q4WBA1]*
PDIG_73060	PDIP_43540	*Similar to Pc22g04150 protein [Blast hit trembl: Penicillium chrysogenum : B6HSM9]*
PDIG_73180	PDIP_43660	*Class I alpha-mannosidase 1A [Ortholog: Aspergillus fumigatus : Q4WLT8]*
PDIG_74350	PDIP_44830	*Phosphoesterase, putative [Ortholog: Aspergillus fumigatus : Q4WQG2]*
PDIG_74370	PDIP_44850	Putative protein
PDIG_74460	PDIP_44940	*Putative uncharacterized protein [Ortholog: Aspergillus fumigatus : Q4WLQ9]*
PDIG_74690	PDIP_45160	Putative protein
PDIG_75150	PDIP_45620	*Similar to Pc22g01400 protein [Blast hit trembl: Penicillium chrysogenum : B6HP38]*

PDIG_75420	PDIP_45890	*Chitinase, putative [Ortholog: Talaromyces stipitatus : B8M4H6]*
PDIG_75600	PDIP_50170	*Carboxypeptidase Y homolog A [Ortholog: Aspergillus fumigatus : Q5VJG9]*
PDIG_75780	PDIP_46250	Putative protein
PDIG_75860	PDIP_46330	Putative protein
PDIG_75930	PDIP_46400	Putative protein
PDIG_76210	PDIP_46680	*Cell wall protein PhiA [Ortholog: Aspergillus fumigatus : Q4WF87]*
PDIG_76410	PDIP_29840	*Similar to Pc13g09460 protein [Blast hit trembl: Penicillium chrysogenum : B6H428]*
PDIG_76430	PDIP_29860	*Putative uncharacterized protein [Blast hit trembl: Aspergillus terreus: Q0CGT3]*
PDIG_76480	PDIP_05060	*1,4-beta-D-glucan-cellobiohydrolyase, putative [Ortholog: Talaromyces stipitatus : B8MK69]*
PDIG_76490	PDIP_05050	Putative protein
PDIG_76620	PDIP_42760	*Putative uncharacterized protein [Ortholog: Aspergillus fumigatus : Q4WBE2]*
PDIG_76850	PDIP_03970	*Oxidoreductin [Ortholog: Aspergillus fumigatus : Q4WCA3]*
PDIG_76970	PDIP_04090	*Endo-arabinase, putative [Ortholog: Aspergillus fumigatus : Q4X046]*
PDIG_77000	PDIP_04120	*Similar to Pc12g01280 protein [Blast hit trembl: Penicillium chrysogenum : B6GYR7]*
PDIG_77100	PDIP_04220	Putative protein
PDIG_77160	PDIP_04280	*Putative uncharacterized protein [Ortholog: Emericella nidulans : Q5AXN2]*
PDIG_77170	PDIP_04290	*Probable glycosidase crf1 [Ortholog: Aspergillus fumigatus : Q8J0P4]*
PDIG_77280	PDIP_04400	*Putative uncharacterized protein [Ortholog: Magnaporthe grisea : A4RGC9]*
PDIG_77300	PDIP_04420	Putative protein
PDIG_77360	PDIP_04480	Putative protein
PDIG_77440	PDIP_04550	*Putative uncharacterized protein [Ortholog: Aspergillus niger : Q3MSB5]*
PDIG_77510	PDIP_04620	*Citrate synthase [Ortholog: Aspergillus fumigatus : A4D9B8]*
PDIG_77520	PDIP_04630	*Similar to Pc12g00660 protein [Blast hit trembl: Penicillium chrysogenum : B6GXU9]*
PDIG_77680	PDIP_04790	*Aldose 1-epimerase, putative [Ortholog: Talaromyces stipitatus : B8M6K3]*
PDIG_78300	PDIP_26720	*Similar to putative uncharacterized protein Pc13g12600 [Blast hit trembl: Penicillium chrysogenum : B6H354]*
PDIG_78510	PDIP_26930	*Putative uncharacterized protein [Ortholog: Aspergillus fumigatus : Q4WSI4]*
PDIG_78620	PDIP_27030	*Endoglucanase, putative [Ortholog: Aspergillus fumigatus : Q6MYM8]*
PDIG_78880	PDIP_27290	*Protein PRY2 [Blast hit: Saccharomyces cerevisiae: P36110]*
PDIG_79130	PDIP_27540	Putative protein
PDIG_79140	PDIP_27550	*Putative uncharacterized protein [Ortholog: Talaromyces stipitatus : B8M267]*
PDIG_79360	PDIP_27750	*Alfa-L-arabinofuranosidase [Ortholog: Penicillium chrysogenum : B5MGR1]*
PDIG_79990	PDIP_28370	*Similar to putative uncharacterized protein Pc13g11090 [Blast hit trembl: Penicillium chrysogenum : B6H3X5]*
PDIG_80010	PDIP_28390	*Putative uncharacterized protein [Ortholog: Aspergillus fumigatus : Q6MYV7]*
PDIG_80170	PDIP_28560	*Predicted protein [Ortholog: Emericella nidulans : Q5AYF8]*
PDIG_80350	PDIP_28740	*Fructosyl amino acid oxidasesarcosine oxidase, putative [Ortholog: Aspergillus fumigatus : Q4WXN4]*
PDIG_80410	PDIP_28800	*Similar to Pc13g10570 protein [Blast hit trembl: Penicillium chrysogenum : B6H3Y9]*
PDIG_80520	PDIP_28910	*Meiotic sister chromatid recombination protein Ish1/Msc1, putative [Ortholog: Aspergillus fumigatus : Q4WXS0]*
PDIG_80660	PDIP_29050	*Similar to putative uncharacterized protein Pc13g10230 [Blast hit trembl: Penicillium chrysogenum : B6H4S3]*
PDIG_81260	PDIP_29610	*Vacuolar protease A [Ortholog: Aspergillus fumigatus : O42630]*
PDIG_81290	-	Putative protein
PDIG_81380	PDIP_29720	*Hypothetical protein [Blast hit trembl: Penicillium chrysogenum : B6H439]*
PDIG_81760	PDIP_30120	Putative protein
PDIG_82430	PDIP_88730	*MOSC domain protein [Ortholog: Aspergillus fumigatus : A4D9P8]*
PDIG_82590	PDIP_86390	*Putative uncharacterized protein [Ortholog: Talaromyces stipitatus : B8LYY3]*
PDIG_82710	PDIP_86500	*1,3-beta-glucanosyltransferase gel4 [Ortholog: Aspergillus fumigatus : P0C956]*
PDIG_82970	PDIP_86760	*Putative uncharacterized protein [Ortholog: Emericella nidulans : Q5AW42]*
PDIG_83060	PDIP_86850	*Putative uncharacterized protein [Ortholog: Emericella nidulans : Q5B856]*
PDIG_83210	PDIP_87000	*Similar to putative uncharacterized protein Pc22g08820 [Blast hit trembl: Penicillium chrysogenum : B6HT41]*
PDIG_83500	PDIP_89000	*Allergen Asp f 15 [Ortholog: Aspergillus fumigatus : O60022]*
PDIG_83870	PDIP_89360	*Mitochondrial large ribosomal subunit protein L16, putative [Ortholog: Aspergillus fumigatus : Q4X0U8]*
PDIG_84720	PDIP_22380	*Similar to Pc13g05210 protein [Blast hit trembl: Penicillium chrysogenum : B6H2F9]*
PDIG_84890	PDIP_22550	Putative protein

PDIG_85230	PDIP_22880	*Putative uncharacterized protein [Ortholog: Talaromyces stipitatus : B8M3V7]*
PDIG_85650	PDIP_47400	*Similar to putative uncharacterized protein Pc20g01700 [Blast hit trembl: Penicillium chrysogenum : B6HDQ5]*
PDIG_85670	PDIP_47380	*Putative uncharacterized protein [Ortholog: Aspergillus fumigatus : Q4WMK6]*
PDIG_85800	PDIP_47270	*Esterase, putative [Ortholog: Aspergillus fumigatus : Q4WZE4]*
PDIG_85970	PDIP_47100	*Tripeptidyl-peptidase sed1 [Ortholog: Aspergillus fumigatus : Q70DX9]*
PDIG_86170	PDIP_32190	Putative protein
PDIG_86180	PDIP_32200	*Predicted protein [Ortholog: Emericella nidulans : Q5B234]*
PDIG_86320	PDIP_32340	*Carboxylesterase family protein [Ortholog: Aspergillus clavatus : A1CFK9]*
PDIG_86820	PDIP_32810	*LPS glycosyltransferase, putative [Ortholog: Aspergillus fumigatus : Q4WB10]*
PDIG_86860	PDIP_32850	*Predicted protein [Ortholog: Uncinocarpus reesii : C4JFX5]*
PDIG_86870	PDIP_32860	*Function: S. cerevisiae frataxin [Ortholog: Aspergillus niger : A2QJ83]*
PDIG_86920	PDIP_32910	*Putative uncharacterized protein [Ortholog: Aspergillus niger : A2QJ78]*
PDIG_87060	PDIP_33060	*50S ribosomal protein L24 [Ortholog: Talaromyces stipitatus : B8LWP4]*
PDIG_87110	PDIP_33120	*Function: gma12p is not the only galactosyltransferase in S. pombe [Ortholog: Aspergillus niger : A2QJ61]*
PDIG_87320	-	Putative protein
PDIG_87520	PDIP_33550	*Putative uncharacterized protein [Ortholog: Emericella nidulans : Q5AWB9]*
PDIG_87530	PDIP_33560	*Putative uncharacterized protein [Ortholog: Emericella nidulans : Q5AWB8]*
PDIG_87550	PDIP_33580	*Putative uncharacterized protein [Ortholog: Emericella nidulans : Q5BAD7]*
PDIG_87690	PDIP_33710	*Putative uncharacterized protein [Ortholog: Aspergillus fumigatus : Q4WQ16]*
PDIG_88040	PDIP_34060	*Pectate lyase [Ortholog: Emericella nidulans : Q5ATC7]*
PDIG_88470	PDIP_31750	*GPI anchor transamidase, putative [Ortholog: Aspergillus fumigatus : Q4WRT8]*
PDIG_88690	PDIP_32000	*Putative uncharacterized protein [Ortholog: Aspergillus fumigatus : Q4X0X1]*
PDIG_88850	-	*Putative uncharacterized protein [Blast hit trembl: Aspergillus clavatus: A1CCV8]*
PDIG_88980	PDIP_03250	*Putative uncharacterized protein [Ortholog: Magnaporthe grisea : A4R9J2]*
PDIG_89180	PDIP_03450	*Putative uncharacterized protein [Ortholog: Talaromyces stipitatus : B8MR87]*
PDIG_89390	PDIP_03660	*Probable beta-glucosidase btgE [Ortholog: Emericella nidulans : Q5BD29]*
PDIG_89450	PDIP_03720	*Similar to Pc12g03260 protein [Blast hit trembl: Penicillium chrysogenum : B6H0V5]*
PDIG_89690	-	*Cell wall protein, putative [Ortholog: Aspergillus clavatus : A1CJT0]*
PDIG_89980	PDIP_06900	*Endo alpha-1,4 polygalactosaminidase precusor [Ortholog: Pyrenophora tritici-repentis : B2WPP4]*
PDIG_90290	PDIP_07220	*Putative uncharacterized protein [Ortholog: Aspergillus fumigatus : Q4WIJ7]*
PDIG_90450	PDIP_07370	*Putative uncharacterized protein [Ortholog: Emericella nidulans : Q5AVK8]*
PDIG_90800	PDIP_07720	*Cellobiose dehydrogenase [Ortholog: Coccidioides posadasiiVerticillium albo-atrum : C9SCR2]*
PDIG_91100	PDIP_08020	*L-asparaginase [Ortholog: Aspergillus fumigatus : Q4WKE2]*
PDIG_91190	-	Minor spike protein H [Blast hit: Enterobacteria phage phiX174: P03646]
PDIG_91370	PDIP_88420	*Putative uncharacterized protein [Ortholog: Aspergillus fumigatus : Q4WW49]*
-	PDIP_25870	Putative protein
-	PDIP_17540	Putative protein
-	PDIP_35490	Putative protein
-	PDIP_41810	Putative protein
-	PDIP_57270	Putative protein
-	PDIP_57730	Putative protein
-	PDIP_60540	Putative protein
-	PDIP_06040	Putative protein
-	PDIP_04990	Putative protein
-	PDIP_85780	Putative protein
-	PDIP_33340	Putative protein
